# Visible‐Light‐Induced Carbon–Hydrogen Phosphonylation of Conjugated Polymers via Electron Donor–Acceptor Complex Activation

**DOI:** 10.1002/anie.4746176

**Published:** 2026-08-05

**Authors:** Tomohiro Tamano, Kohei Taniguchi, Yoichiro Eriguchi, Kosuke Sato, Shinsuke Inagi

**Affiliations:** ^1^ Department of Chemical Science and Engineering School of Materials and Chemical Technology, Institute of Science Tokyo Yokohama Japan; ^2^ Japan Ground Self‐Defense Force Tokyo Japan

**Keywords:** conjugated polymer, electron donor–acceptor complex, phosphonylation, photochemical reaction, postfunctionalization

## Abstract

Electron donor–acceptor (EDA) complexation has emerged as a sustainable strategy for visible‐light‐driven reactions. Although numerous EDA‐based transformations have been applied to small aromatic molecules, conjugated polymers (CPs) have not yet been explored as substrates for EDA‐mediated functionalization. Polymer‐based EDA complexes comprise extended π‐conjugation systems that reduce exciton‐binding energy and facilitate red‐shifted absorption, thereby enabling efficient visible‐light harvesting. These characteristics are expected to intrinsically promote photoinduced functionalization. Here, we demonstrate the photochemical phosphonylation of poly(9,9‐dioctylfluorene) (PFO) induced via the visible‐light activation of its EDA complex. Compared with a monomeric model compound, the reaction of PFO proceeded more efficiently, indicating that CPs are promising substrates for EDA‐excitation‐initiated photochemical transformations. Phosphonylation predominantly proceeded at the 4‐position of the fluorene units, thereby preserving the effective π‐conjugation length of the polymer backbone. Furthermore, density functional theory calculations revealed that the greater stability of the intermediate species and the lower excitation energy required for 4‐position substitution favored that pathway over 3‐position substitution. This study establishes a new platform for integrating CPs into EDA‐mediated photochemical reactions, offering a facile and sustainable route for the synthesis of versatile functional polymer materials.

## Graphical abstract

Electron donor–acceptor (EDA) complex activation enabled the efficient transformation of conjugated polymers. Compared with the small‐molecule model compound, the polymers reacted more efficiently, achieving quantitative conversion of PFO. This result demonstrates a new platform for incorporating conjugated polymers into EDA‐mediated photochemical reactions.

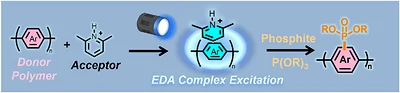

## 1 Introduction

Photochemistry has emerged as a transformative tool in organic synthesis, providing innovative pathways for chemical transformations. Over the past decade, photochemical functionalization has evolved from a niche technique into a broadly applicable strategy for forming C–C and C–heteroatom bonds under mild conditions, often at ambient temperature and with high functional‐group tolerance [1]. Among these transformations, C(sp^2^)–P bond formation has attracted considerable attention owing to its applications in medicinal chemistry [2, 3, 4], organic electronics [5, 6, 7, 8, 9], and chemical sensing [10, 11, 12].

Photoredox‐catalyzed [13, 14, 15, 16] phosphonylation reactions have been extensively investigated as milder, more cost‐effective approaches to forming C(sp^2^)–P bonds compared with conventional transition metal‐catalyzed [17, 18, 19, 20] and electrochemical methods [21, 22]. However, these strategies typically require external photocatalysts, thereby complicating efforts toward more sustainable, greener synthetic protocols.

More recently, the formation of electron donor–acceptor (EDA) complexes between substrates and additives/reactants has emerged as a sustainable alternative that eliminates the need for exogenous photocatalysts. In this protocol, a ground state EDA complex typically exhibits absorption bands in the visible region, and photoexcitation induces single‐electron transfer (SET), generating a radical ion pair (Figure 1a). Such radical intermediates have driven the formation of C–C [23, 24, 25, 26, 27], C–O [28], C–N [29, 30, 31], C–P [32], and C–S [33] bonds via the direct C–H functionalization of small organic molecules (Figure 1a). Additionally, the EDA‐mediated phosphonylation of small aromatic molecules has been performed using phosphites (P(OR)_3_) and appropriate leaving groups (Figure 1a) [34, 35, 36, 37]. However, to the best of our knowledge, EDA‐induced photochemical reactions of conjugated polymers (CPs) have not been reported. Efficient post‐phosphonylation of CPs could substantially expand the applicability of functional polymers in organic electronics and organic ion conductors.

**FIGURE 1:**
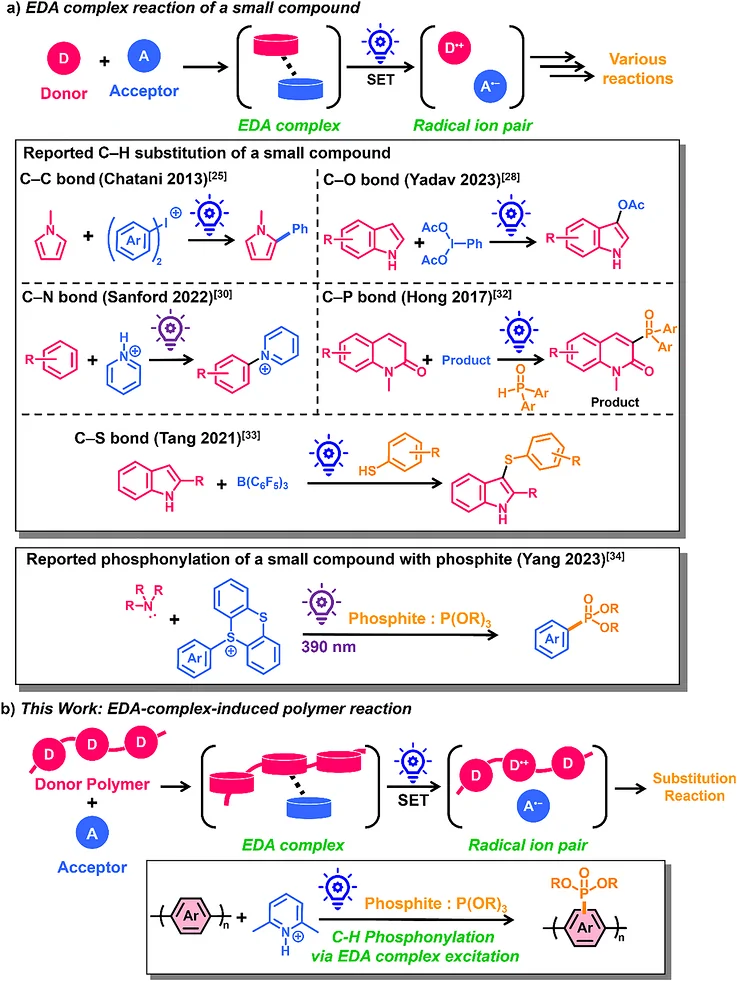
Strategy for electron donor–acceptor (EDA)‐complex‐induced photochemical reactions of (a) small aromatic molecules and (b) conjugated polymers.

In this study, we propose that EDA‐induced photochemical functionalization of CPs may impart distinct advantages over small‐molecule systems. CPs generally exhibit red‐shifted absorption relative to small aromatics owing to their extended π‐conjugated backbones. This extended conjugation can (i) broaden absorption to longer wavelengths, enabling more efficient activation under visible light, and (ii) reduce exciton‐binding energy, facilitating ion‐pair generation [38]. Guided by these considerations, we hypothesized that CPs could impart EDA‐mediated photochemical reactions with notable benefits. To test this hypothesis, we demonstrate the efficient photochemical phosphonylation of polyfluorene using a model system comprising a lutidinium acceptor and a phosphite (Figure 1b).

## 2 Results and Discussion

### 2.1 Development and Optimization of Photochemical Phosphonylation

We performed the photochemical phosphonylation of poly(9,9‐dioctylfluorene) (PFO) in dichloroethane (DCE), employing 2,6‐lutidinium perchlorate (Lut–H^+^ClO_4_^−^) as an electron acceptor and triethyl phosphite at room temperature in an argon atmosphere (Figure 2a). Upon irradiation with visible blue light‐emitting diodes (LEDs, Figure S1), phosphonate groups were incorporated into the PFO backbone, realizing an overall 80% (14% and 66% for the 3‐and 4‐positions, respectively) degree of functionalization (DOF, defined as the molar ratio of incorporated phosphonate groups to the total number of monomer units), as shown in Figures 2a and S2. The proton (^1^H) and phosphorus‐31 (^31^P) nuclear magnetic resonance (NMR) signals of the resulting polymer (**P1**) were assigned based on established electrochemical phosphonylation data [39]; the DOF was determined by comparing the integral ^1^H NMR signals of the methylene protons of the phosphonate groups with those of the octyl chains and the α‐position protons of the phosphonate groups with the others in the aromatic region (Figure S3 and Table S1).

**FIGURE 2:**
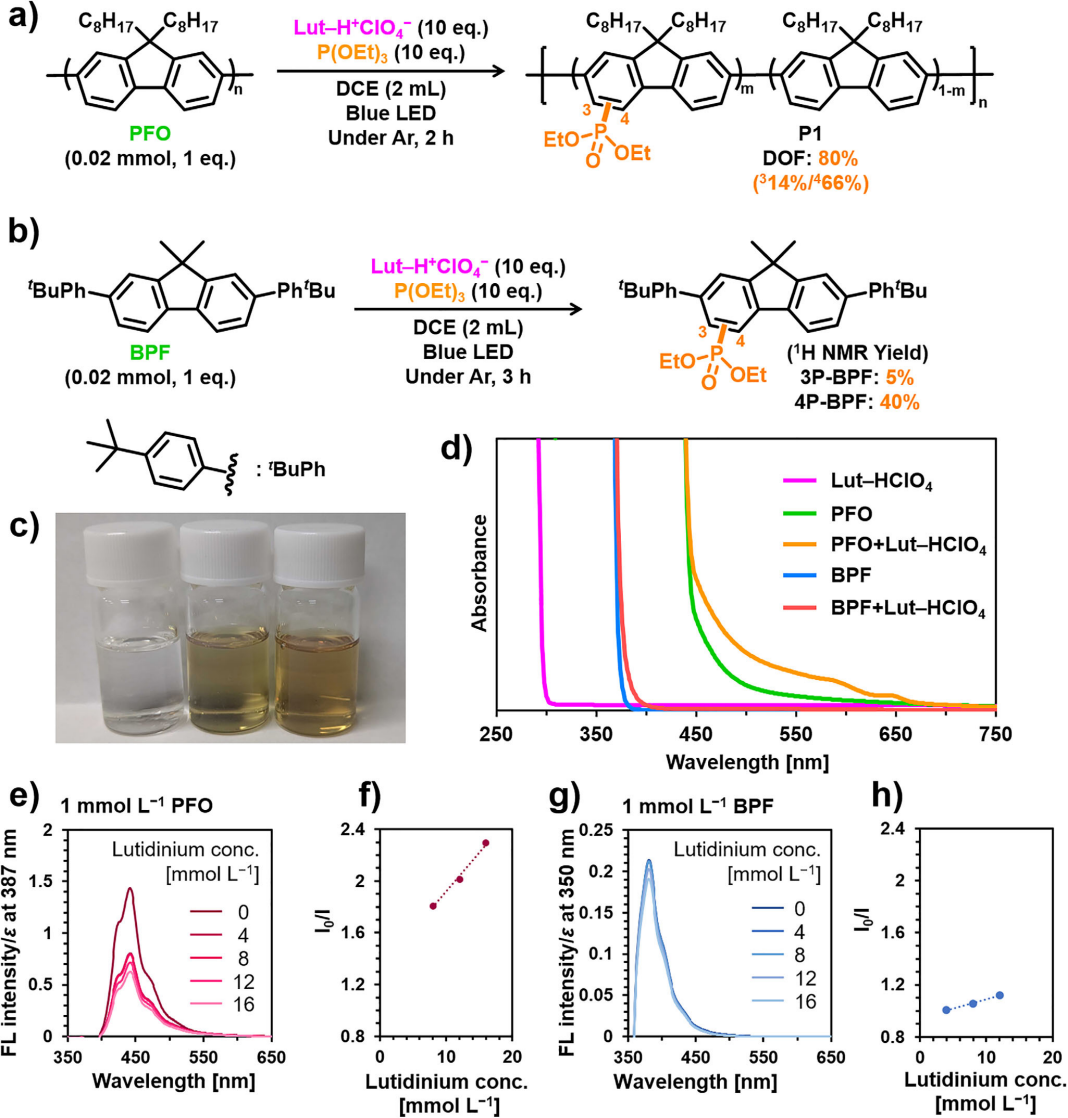
Photochemical phosphonylation of (a) poly(9,9‐dioctylfluorene) (PFO) and (b) 2,7‐bis(4‐(tert‐butyl)phenyl)‐9,9‐dimethyl‐9*H*‐fluorene (BPF). (c) Photographs of PFO (center), 2,6‐lutidinium perchlorate (Lut–H^+^ClO_4_^−^; left), and PFO with Lut–H^+^ClO_4_^−^ (right). (d) Ultraviolet–visible (UV–Vis) spectra of PFO (green line), Lut–H^+^ClO_4_^−^ (pink line), PFO + Lut–H^+^ClO_4_^−^ (orange line), BPF (blue line), and BPF + Lut–H^+^ClO_4_^−^ (red line), and the emission spectrum of the blue LED (navy dotted line). UV–Vis spectra of the reaction mixtures were measured under identical concentrations to the reaction conditions [PFO (0.02 mmol) and Lut–H^+^ClO_4_^−^ (0.2 mmol) in a dichloroethane (DCE) solution (2 mL)]. Fluorescence (FL) quenching of 1 mmol L^−1^ (e) PFO and (g) BPF upon addition of Lut–H^+^ClO_4_^−^. Stern–Volmer plots of (f) PFO and (h) BPF.

To benchmark against a small aromatic compound, we implemented the photochemical reaction using 2,7‐bis(4‐(tert‐butyl) phenyl)‐9,9‐dimethyl‐9*H*‐fluorene (BPF) as a model compound, obtaining 5% and 40% yields at the 3‐ and 4‐positions, respectively. These results indicate that, under blue LED excitation, the EDA‐complex‐induced photochemical reaction of CPs is more favorable than that of the small‐molecule systems.

Subsequently, we investigated the feasibility of forming an EDA complex between PFO and Lut–H^+^ClO_4_^−^. Upon mixing, the dark yellow PFO solution and colorless Lut^+^ClO_4_^−^ solutions turned dark brown (Figure 2c), indicating the formation of the EDA complex. To confirm this color change and further examine complex formation, we recorded the ultraviolet–visible (UV–Vis) absorption spectrum of the mixed solution (Figures 2d and S4). This spectrum (orange line) exhibited a pronounced red shift compared with the isolated spectra of individual components (red and green lines in Figure 2d). Conversely, no spectral change was observed upon comparison of a Lut–H^+^ClO_4_^−^ solution with a mixture of triethyl phosphite (P(OEt)_3_) and Lut–H^+^ClO_4_^−^ (Figure S4). These results confirm the formation of an EDA complex between PFO and 2,6‐lutidinium, with enhanced absorption overlapping the emission wavelength range of the blue LED source. Furthermore, the EDA complex formed between BPF and 2,6‐lutidinium exhibited a weak absorption band in the blue LED region (Figures 2d and S4), indicating that the polymeric π‐conjugation contributes to the formation of the visible‐light‐active EDA complex. In addition, the temperature of the reaction mixture was up to 45°C after 3 h of irradiation, implying that the heat did not significantly affect the reaction progress.

To gain further insight into the SET behavior of the EDA complex, we performed the Stern–Volmer analyses for PFO and BPF upon the addition of Lut–H^+^ClO_4_^−^ (Figures 2e–h and S5). The results reveal that the fluorescence (FL) intensity of 1 mmol L^−1^ PFO in DCE decreased with the increasing Lut–H^+^ClO_4_^−^ concentration (Figures 2e and S5) and that the Stern–Volmer plot exhibited a linear relationship (Figure 2f). Although BPF exhibited a similar trend in the presence of 2,6‐lutidinium, its EDA‐induced quenching was less efficient than that of PFO (Figure 2g,h). This quenching in PFO relative to BPF was further validated by comparing FL intensities normalized to their respective molar absorption coefficients (*ε*), alongside the observed decreases in FL quantum yield (*Φ*) (Figure S5). These observations indicate that the SET from PFO to 2,6‐lutidinium occurs under visible‐light irradiation and that polymeric substrates were more favorable for efficient EDA‐based excitation than monomeric compounds.

Next, we optimized the reaction conditions (Table 1), revealing that DCE was the most efficient solvent, whereas 1,4‐dioxane and toluene yielded lower DOF (Entries 1–3). Additionally, BF_4_^−^ was found to be unsuitable for this reaction (Entry 4). Further analysis revealed that 10 equivalents of Lut–H^+^ClO_4_^−^and phosphite yielded the optimal results (Entries 5 and 6). Time‐course studies revealed that the DOF reached 100% within only 3 h (Entry 7), and prolonging the reaction beyond this period did not exert any influence on the DOF. The isolated yield (88%) was calculated from the weight of the resulting polymers, considering the DOF. Notably, a tunable DOF was achieved within the initial 3 h (Table S2, Figures S6 and S7). Furthermore, the reaction was repeated three times to verify its reproducibility, yielding highly consistent results (Figure S6). These data confirm that the functionalization is highly reproducible and proceeds with minimal material loss. In addtion, although the DOF decreased to 65% under ambient conditions, the phosphonylation was still achieved (Table 1, Entry 8). Control experiments also confirmed the criticality of Lut–H^+^ClO_4_^−^ and blue light to the reaction (Entries 9 and 10), supporting the hypothesis that the transformation proceeds via photoexcitation of the EDA complex, as described above. Furthermore, initiating the reaction under sunlight for one week yielded a DOF of 16% (Figure S8). Compared with standard light irradiation, the DOF decreased, substantially extending the required reaction time, probably because of the lower light intensity. Nevertheless, these results demonstrate that sunlight can, in principle, activate the EDA complex and promote this reaction. In addition, we performed a larger‐scale phosphonylation (a 5‐fold scale‐up), which yielded a sample with a 99% DOF after 3 h (Table 1, Entry 11). The effects of PFO molecular weight were also investigated using a low‐molecular‐weight PFO (LW‐PFO; *M*_n_ = 8600 g mol^−1^, *M*_w_ = 14 200 g mol^−1^, *Đ* = 1.7) and a high‐molecular‐weight PFO (HW‐PFO; *M*_n_ = 54 700 g mol^−1^, *M*_w_ = 198 000 g mol^−1^, *Đ* = 3.6). Under the condition of Entry 7, the LW‐PFO achieved a DOF of 95%, demonstrating an almost quantitative reaction. Conversely, the reaction with HW‐PFO proved difficult due to its poor solubility in DCE. These results indicate that while the photochemical reaction is highly effective for polymers, substrate solubility remains a primary limiting factor.

**TABLE 1:** Optimization of reaction conditions for the EDA‐assisted photochemical phosphonylation of PFO.

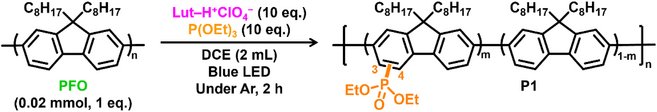				
**Entry**	**Deviation from the above**	**DOF (%)^a^**		
		**3‐Position**	**4‐Position**	**Total**
1^b^	None	11	64	75
2	1,4‐Dioxane instead of DCE	0	24	24
3	Toluene instead of DCE	0	19	19
4	Lut–H^+^BF_4_^−^ instead of Lut–H^+^ClO_4_^−^	8	30	38
5	Lut–H^+^ClO_4_^−^ and P(OEt)_3_ (5 eq.)	9	41	50
6	Lut–H^+^ClO_4_^−^ and P(OEt)_3_ (15 eq.)	20	60	80
7^b^	3 h instead of 2 h	23	73	96
8	Under air and 4 h	13	52	65
9	Without Lut–H^+^ClO_4_^−^	0	5	5
10	Without light	0	0	0
11	5‐fold scale‐up, same concentration, 3 h	23	77	99

^a^ Degree of functionalization (DOF) was determined by proton nuclear magnetic resonance (^1^H NMR).

^b^ The reaction was conducted three times, and the average is shown.

### 2.2 Scope of Trialkyl Phosphites and Precursor Polymers

We investigated trialkyl phosphite reagents and precursor polymers (Scheme 1). First, the reactivity of trialkyl phosphites was evaluated. P(OR)_3_ with longer linear alkyl chains, such as tri‐n‐butyl (**P2**) and tri‐n‐hexyl (**P3**), exhibited compatibility for the reaction, yielding DOFs of 81% and 74%, respectively. Branched alkyl chains, including triisopropyl (**P4**) and tri(2‐ethylhexyl) (**P5**), also participated in the reaction, with DOFs of 100% and 69%, respectively. These results indicate that a broad range of trialkyl phosphites can be applied in the photochemical phosphonylation.

**SCHEME 1:**
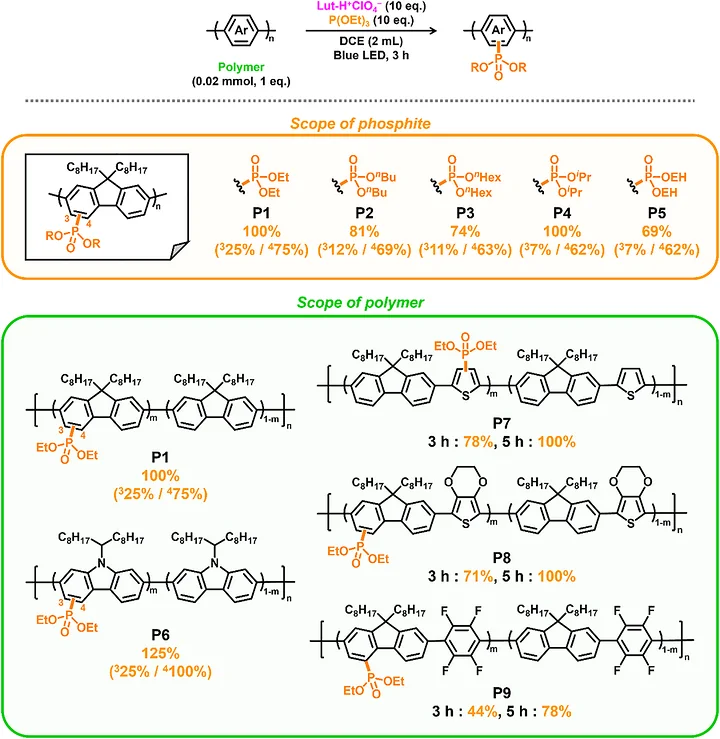
Scope of trialkyl phosphites and precursor polymers for the EDA‐induced photochemical phosphonylation of CPs.

Next, we explored the photochemical phosphonylation of poly(9‐(9‐heptadecyl)carbazole) (**Pre6**), fluorene–thiophene alternating copolymer (**Pre7**, Figure S9), fluorene‐3,4‐ethylenedioxythiophene alternating copolymer (**Pre8**), and fluorene‐tetrafluorophenylene alternating copolymer (**Pre9**), yielding the corresponding phosphonate‐containing polymers (**P6**–**P9**), respectively. For **P6**, the DOF increased relative to **P1**, likely because of the electron‐rich nature of the carbazole rings. The reactions of **Pre7** and **Pre8** proceeded efficiently, with DOF of 78% and 71% for **P7** and **P8**, respectively, after 3 h. Extending the reaction time to 5 h allowed both reactions to reach quantitative conversion. Although the DOF for **P9** decreased, the photochemical phosphonylation was completed, with selective functionalization occurring at the 4‐position of the fluorene unit. We observed that certain precursor polymers exhibited absorption bands around 470 nm, overlapping with the emission range of the blue LED source (Figure 3). For such polymer substrates, direct photoexcitation of the polymer backbone is also expected to contribute to the reaction.

**FIGURE 3:**
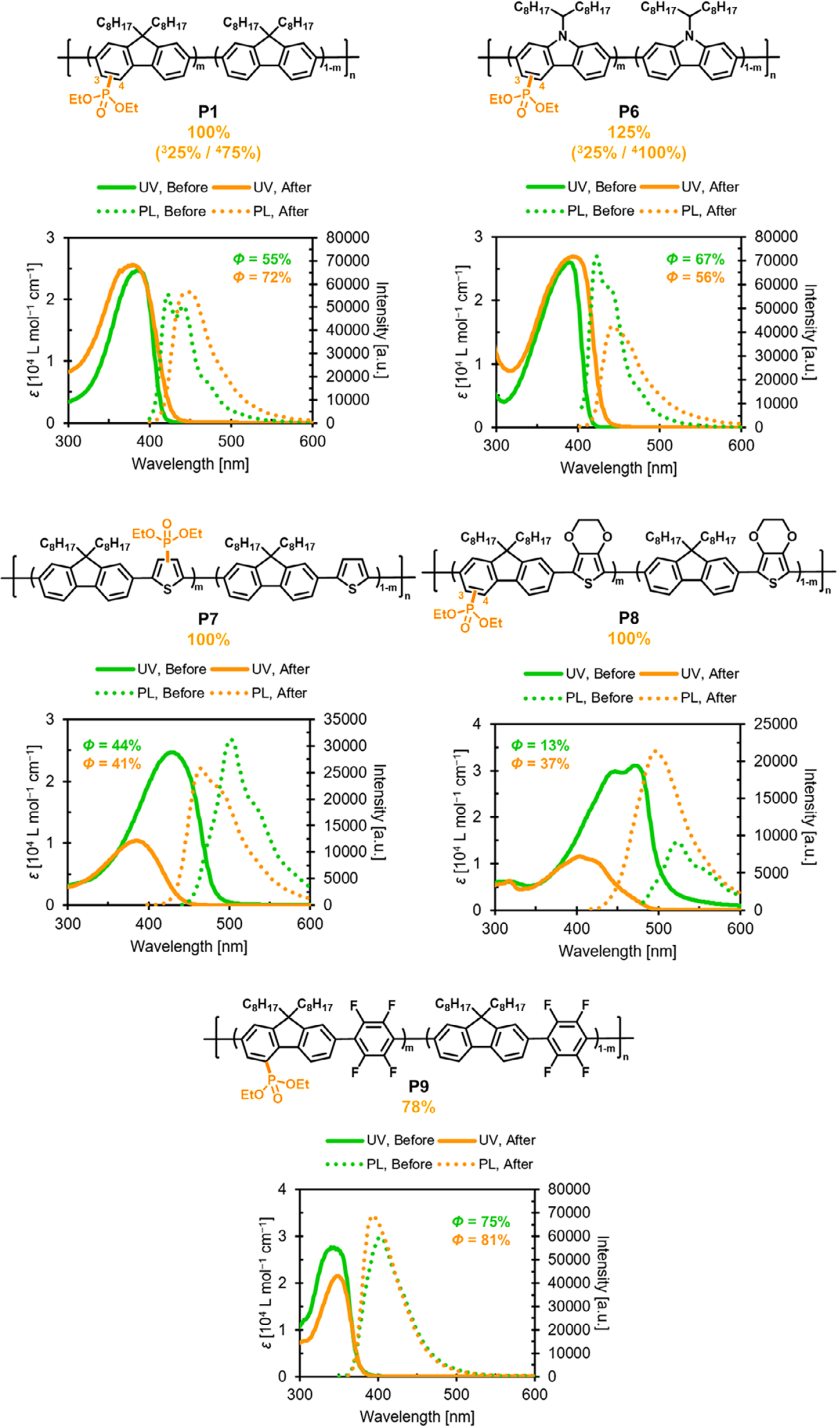
UV–Vis absorption and FL spectra of the polymers before (light‐green line) and after (orange line) phosphonylation (**P1**, **P6**, **P7**, **P8**, and **P9**). Spectra measured in a 1.0 × 10^−4^ mol L^−1^ DCE solution.

### 2.3 Optical Properties of Phosphonylated Polymers

The optical properties of the phosphonylated polymers were systematically investigated. Figure 3 presents the UV–Vis absorption spectra of the precursor and phosphonylated polymers in solution. The phosphonylation reaction induced two distinct types of spectroscopic changes, depending on the structure of the substrate. For **P6** and **P7**, the absorption and FL spectra exhibited a blue shift and the molar absorption coefficient decreased, attributed to the introduction of phosphonate groups. These groups induced the twisting of the polymer backbone, thereby reducing the effective π‐conjugation length, consistent with previous reports [21, 22]. Conversely, the absorption spectra for **P1**, **P5**, and **P8** remained almost unchanged or only slightly redshifted.

To elucidate the origin of these divergent spectroscopic behaviors, density functional theory (DFT) calculations were performed using 9,9‐diethyl‐2‐phenylfluorene (PF) and its phosphonylated derivatives at the 3‐ and 4‐positions (3P‐PF and 4P‐PF, respectively) as model compounds. The DFT‐calculated dihedral angles for 3P‐PF, 4P‐PF, and PF were 63.0°, 37.7°, and 40.9°, respectively (Figure S10), indicating that phosphonylation at the 4‐position extends the effective π‐conjugation length, whereas substitution at the 3‐position considerably twists the polymer backbone and shortens the conjugation length. The coexistence of these opposing electronic effects likely accounts for the minimal changes observed in the experimental absorption spectra. Time‐dependent DFT (TD‐DFT) calculations further revealed that the absorption maximum for 4P‐PF was redshifted relative to PF, whereas that of 3P–PF was blueshifted relative to PF (Figures S11–S13). Furthermore, the phosphonylated CPs maintain steady or increased quantum yields (Figure 3). Although our DFT calculations showed a decrease in oscillator strength for both 3‐ and 4‐position substitutions—due to LUMO localization on the phosphonate (Figures S11–S13)—this is offset by steric effects. According to literature [40], bulky side chains create steric hindrance that restricts intramolecular free rotation, effectively suppressing non‐radiative decay and improving quantum efficiency. The bulky diethyl phosphonate groups act similarly in our system.

### 2.4 DFT‐Based Investigation of the Reaction Mechanism and Selectivity of the Phosphonylated Polymers

Regarding the reaction mechanism, the substitution pattern of the phosphonate groups exhibited intriguing trends: the 4‐position of the fluorene rings (^4^DOF/^3^DOF = 75/25) was favorable, showing reversed selectivity compared with the typical nucleophilic scenario [41] and the electrochemical phosphonylation case (^4^DOF/^3^DOF = 28/63) [39]. To investigate this difference, DFT calculations were performed using 9,9‐dimethyl‐2‐phenylfluorene (PF‐M) as a model compound.

Based on the EDA‐complex formation and SET behavior revealed by the Stern–Volmer plots (Figure 2e,f), together with previously reported electrooxidation‐initiated phosphonylation mechanisms [39], we implemented a comparable photochemical reaction pathway: initial photochemical oxidation of PFO followed by the nucleophilic addition of phosphite, subsequent phosphonate formation, a second photochemical oxidation, and final deprotonation to yield phosphonylated PFO (Figure 4a).

**FIGURE 4:**
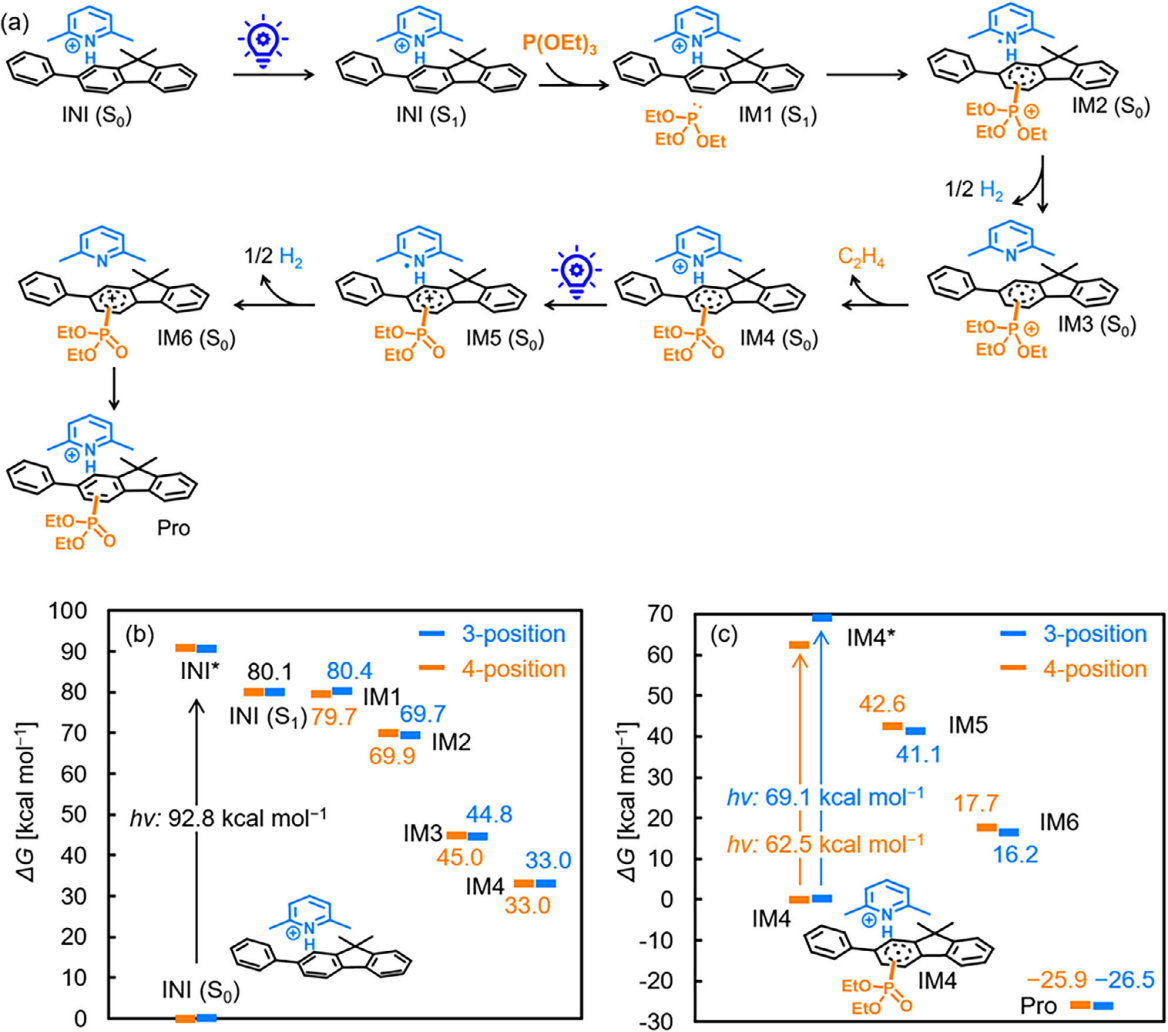
(a) Reaction mechanism of visible‐light‐induced C–H phosphonylation of PF‐M. (b, c) Relative Gibbs free energies (Δ*G*) of the phosphonylation pathways for the 3‐position (blue) and the 4‐position (orange) substitutions.

All calculations were performed at the *ω*B97XD/6‐31G+(d,p) level of theory, and TD‐DFT was performed to compute the S_1_ excited state. Before constructing the energy diagrams, the optimal position of 2,6‐lutidinium relative to PF‐M was determined (Table S3). Based on the calculated stabilization energy of the PF‐M/2,6‐lutidinium‐complex state, the position at which the 2,6‐lutidinium ring interacted with the benzene ring of fluorene, with the H–N^+^ moiety facing the PF‐M alkyl chain, was employed for subsequent mechanistic analyses, as shown in the chemical structures displayed in Figures 4b,c, as well as Complex D in Table S3. Figure 4b presents the calculated Gibbs free energy (Δ*G*) profiles from the initial state (INI) to intermediate 4 (IM4), and Figure 4c shows the profiles from IM4 to the product. A comparison of the energy diagrams for the 4‐position and 3‐position reactions indicates that phosphonylation at the 4‐position is more favorable. From the first singlet excitation state (INI (S_1_)) to intermediate 1 (IM1), the 4‐position reaction exhibited a downhill sequence with a difference of −0.4 kcal mol^−1^, whereas the 3‐position reaction requires an uphill sequence of +0.3 kcal mol^−1^ (Figure 4b). Although this energy difference is not significant, it aligns with the experimentally observed weak selectivity (^4^DOF/^3^DOF = 66/14). Furthermore, the excitation energy of IM4 in the 4‐position reaction (62.5 kcal mol^−1^, 2.7 eV) was lower than that in the 3‐position reaction (69.1 kcal mol^−1^, 3.0 eV), indicating a difference in the light‐absorption efficiency during the second SET step (Figure 4c). Overall, these computational results identified the 4‐position substitution as the preferred pathway while highlighting the potential for competitive 3‐position substitution.

## 3 Conclusion

In summary, we have demonstrated the photochemical phosphonylation of PFO driven by the visible‐light‐induced excitation of its EDA complex. Compared with the small‐molecule model compound, the reaction of CPs proceeded more efficiently under blue light irradiation, achieving quantitative PFO conversion. As hypothesized, CPs are particularly well‐suited for photochemical functionalization: their extended conjugation facilitates efficient donor–acceptor SET and broadens the photo‐absorption spectrum to longer wavelengths. Moreover, the photochemical reaction of PFO exhibited compatibility with a range of trialkyl phosphite reagents, including tributyl phosphite, trihexyl phosphite, triisopropyl phosphite, and tris(2‐ethylhexyl) phosphite. Further, this reaction pathway was applied to various CPs (**Pre6**–**Pre9**), highlighting the versatility of the EDA‐complex excitation strategy. Phosphonylation proceeded predominantly at the 4‐position, in clear contrast to conventional and electrochemical phosphonylation pathways. Subsequent DFT calculations attributed this site selectivity to the relative stability of the IM1 state and the lower excitation energy required for the second SET step in the 4‐position substitution pathway. Furthermore, the 4‐position phosphonylation preserved the effective π‐conjugation length and yielded longer wavelengths, imparting FL properties.

These findings establish photochemical phosphonylation driven by EDA‐complex activation as a robust strategy for selectively functionalizing the 4‐position in fluorene units while preserving the intrinsic optoelectronic properties of the polymers. Such preservation renders these functionalized polymers promising candidates for active‐layer materials in organic photovoltaics [42, 43], organic transistors [5], and OLED applications. We anticipate that this approach will advance the scope of materials chemistry and expose novel avenues for the post‐synthetic functionalization of CPs with a broader range of nucleophiles and radical‐trapping reagents.

## Author Contributions

**Tomohiro Tamano**: investigation, conceptualization, writing – original draft. **Kohei Taniguchi**: writing – original draft, conceptualization, funding acquisition, investigation. **Yoichiro Eriguchi**: investigation, validation. **Kosuke Sato**: writing – review and editing, investigation. **Shinsuke Inagi**: conceptualization, funding acquisition, writing – review and editing, supervision.

## Conflicts of Interest

The authors declare no conflicts of interest.

## Supporting information



The authors have cited additional references within the Supporting Information [44, 45, 46, 47, 48, 49, 50, 51, 52, 53, 54, 55].**Supporting File**: anie73961‐sup‐0001‐SuppMat.pdf.

## Data Availability

The data that support the findings of this study are available from the corresponding author upon reasonable request.
