# Supplementary material for: Visible‐Light‐Induced Carbon–Hydrogen Phosphonylation of Conjugated Polymers via Electron Donor–Acceptor Complex Activation
Source: Angew Chem Int Ed Engl. 2026 Aug 5;65(40):e4746176. doi: 10.1002/anie.4746176 (PMC13618260; doi:10.1002/anie.4746176)
Supplement: Supplementary file 1 [file ANIE-65-e4746176-s001.pdf]

Supporting Information

Visible-Light-Induced Carbon–Hydrogen Phosphonylation of Conjugated Polymers via Electron Donor–Acceptor Complex Activation

*Tomohiro Tamano[1†] Kohei Taniguchi,[1†] Yoichiro Eriguchi[1,2†] Kosuke Sato,[1] Shinsuke Inagi[1]**

[1] Department of Chemical Science and Engineering, School of Materials and Chemical Technology, Institute of Science Tokyo, 4259 Nagatsuta-cho, Midori-ku, Yokohama 226-8502, Japan

[2] Japan Ground Self-Defense Force, Ichigaya Garrison, 5-1 Ichigaya Honmura-cho, Shinjuku-ku, Tokyo, Japan

[†] These authors contributed equally.

Content

1. **Material** 2
2. **General** 2
3. **Synthesis** 4
4. **General procedures for photochemical phosphonylation of PFO** 12
5. **The study of NMR and IR spectra of PFO-Phos(Et)** 13
6. **UV-vis absorption spectra and Stern–Volmer analysis** 15
7. **The relationship between the reaction time and DOF** 17
8. **Sunlight reaction of PFO** 19
10. **Calculation of dihedral angles by DFT calculation** 21
11. **TD DFT calculation** 22
12. **DFT simulation about stable structure of EDA complex** 24
13. **DFT calculation** 25
14. **NMR spectra** 48

1. Material

2,5-Dibromothiophene, 9,9-dioctyl-2,5-dibromofluorene, triethyl phosphite ($P(OEt)_3$), tributyl phosphite ($P(O^nBu)_3$), trihexyl phosphite ($P(O^nHex)_3$), tris(2-ethylhexyl) phosphite ($P(OEH)_3$), triisopropyl phosphite ($P(O^iPr)_3$), anhydrous dichloroethane (DCE), 2,7-dibromo-9-(9-heptadecyl)carbazole, 9-(9-heptadecanyl)-2,7-bis(4,4,5,5-tetramethyl-1,3,2-dioxa-borolan-2-yl)carbazole, 2,5-dibromothiophene, 1,8-diazabicyclo[5.4.0]-7-undecene (DBU), 4-tert-butylbenzeneboronic acid, 9,9-dimethyl-2,7-dibromo fluorene, tetrakis(triphenylphosphine)palladium(0) ($Pd(PPh_3)_4$), and 2,6-lutidine were purchased from TCI (Tokyo Chemical Industry Co., Ltd.). Perchloric acid, tetrafluoroboric acid, potassium carbonate (K_2CO_3), potassium phosphonate (K_3PO_4), anhydrous acetonitrile (MeCN), hydrogen chloride (HCl), ethylenediaminetetraacetic acid tetrasodium salt (EDTA–4Na), anhydrous toluene and anhydrous dioxane were purchased from FUJIFILM Wako Pure Chemical Corporation. Triethyl phosphite ($P(OEt)_3$), 9,9-dioctylfluorene-2,7-diboronic acid ester, and palladium(II) chloride ($PdCl_2$) were purchased from Sigma-Aldrich. Acetone and methanol (MeOH) were purchased from Godo. Chloroform-d_1 ($CDCl_3$), hexane, and sodium sulfonate (Na_2SO_4) were purchased from Kanto Chemical. $CHCl_3$ was purchased from Shinetsu.

2. General

NMR analysis

All NMR spectra were recorded on a Bruker model AVANCE III HD-500 spectrometer (500.13 MHz for ^{1}H, 125.76 MHz for ^{13}C, and 200.44 MHz for ^{31}P) spectrometer using $CDCl_3$ as deuterated solvents at room temperature. The chemical shifts for ^{1}H and ^{13}C NMR spectra are given in δ (ppm) upfield from internal tetramethylsilane (^{1}H NMR: 0 ppm) and $CDCl_3$ (^{13}C NMR: 77.16 ppm), respectively.

Gel permeation chromatography (GPC)

GPC analyses were performed on a Tosoh HLC-8420GPC equipped with Tosoh TSK-Gel G2500H_{HR} columns using chloroform as an eluent. The narrow disperse linear polystyrene standards were used for calibration.

UV–vis absorption spectra

UV–vis absorption spectra were obtained on a Shimadzu UV-1900 spectrophotometer. Spectra in Figure S5 were obtained from a 1.0×10^{-4} mol L^{-1} polymer/chloroform solution.

Fluorescence (FL) spectra

Fluorescence (FL) spectra were recorded using a Shimadzu RF-6000 spectrophotometer. For Figure 2, the excitation wavelengths were set to 387 nm for PFO and 350 nm for BPF. For Figure S5, the excitation wavelengths were set to 388 nm (PFO), 378 nm (P1), 391 nm (P5), 394 nm (phosphonylated P5), 430 nm (P6), 394 nm (phosphonylated P6), 472 nm (P7), 404 nm (phosphonylated P7), 343 nm (P8), and 349 nm (phosphonylated P8).

Computational simulation

Density functional theory (DFT) calculations were performed using Gaussian 16 software[44] at the ωB97XD and B3LYP levels of theory using the 6-31G+(d,p) and 6-311G++(d,p) basis set for optimization and frequency.

3. Synthesis

Synthesis of lutidinium perchlorate Lut–H^{+}ClO$_4^-$

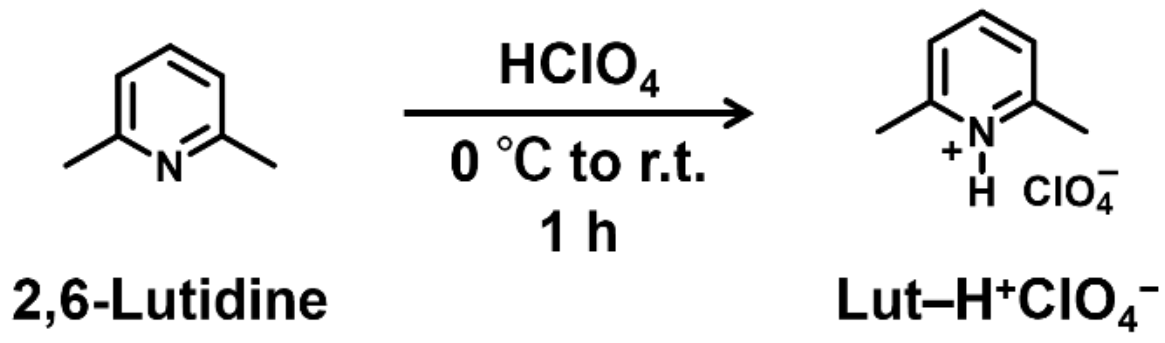

Lut–H^{+}ClO$_4^-$ was synthesized according to the literature.[45] 2,6-lutidine (0.10 mol, 10.7g) was loaded into a 100 mL two-neck flask, and then the flask was cooled to 0 °C. Perchloric acid was then added slowly to the flask. The resulting mixture was stirred at room temperature for 60 min. The reaction mixture was filtered, and the obtained solid was washed with ethyl acetate three times. The product was then dried under vacuum and obtained as a white solid (20.4g, 98 mmol, 98%). ^{1}H NMR (500.13 MHz, CDCl$_3$, ppm): δ = 8.22 (t, J = 7.9 Hz, 1H), 7.55 (d, J = 7.9 Hz, 2H), 2.92 (s, 6H).

Synthesis of lutidinium tetrafluoroborate Lut–H^{+}BF$_4^-$

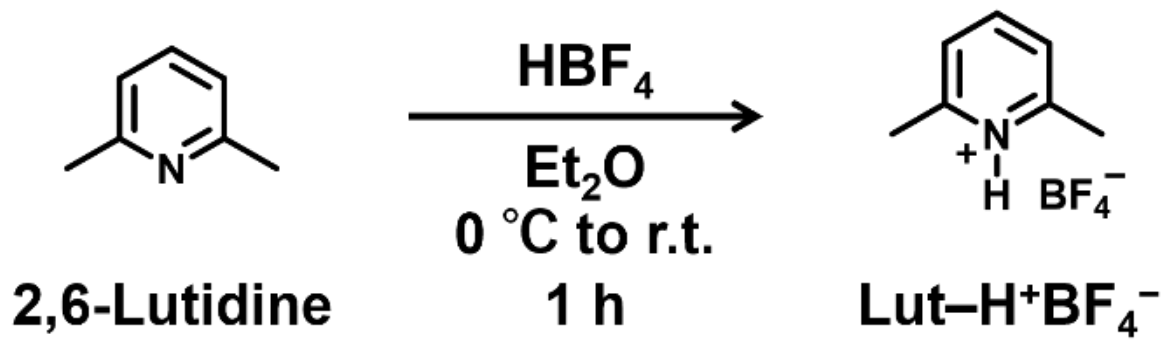

Lut–H^{+}BF$_4^-$ was synthesized according to the literature.[46] 2,6-lutidine (33 mmol, 3.5g) and diethyl ether were loaded into 50 mL two-neck flask, and then the flask was cooled to 0 °C. After that, tetrafluoroboric acid was added slowly to the flask. The mixture was stirred at room temperature for 60 min. The reaction mixture was filtered, and collected solid was washed by ethyl acetate three times, and then the product was dried in vacuum and obtained as a white solid (3.2g, 17 mmol, 56%). ^{1}H NMR (500.13 MHz, CDCl$_3$, ppm): δ = 8.21 (t, J = 7.9 Hz, 1H), 7.55 (d, J = 7.9 Hz, 2H), 2.88 (s, 6H).

Synthesis of poly(9,9-dioctylfluorene) PFO

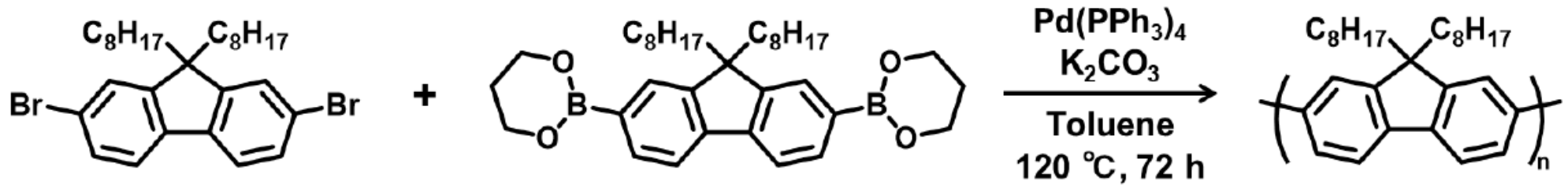

PFO was synthesized according to the literature.[22,47] 9,9-Dioctylfluorene-2,7-diboronic acid bis(1,3-propanediol) ester (3.27 g, 5.85 mmol), 2,7-dibromo-9,9-dioctylfluorene (3.21 g, 5.85 mmol), and $Pd(PPh_3)_4$ (155.48 mg, 0.13 mmol) were loaded into a Schlenk flask. The flask was vacuumed for 30 min and replaced with argon, and then anhydrous toluene (30 mL), and degassed 2M K_2CO_3 aq. (18 mL) were added. The mixture was stirred under reflux for 72 h. The whole mixture was diluted with $CHCl_3$, and the chloroform solution was poured into a large amount of 0.1 M EDTA·4Na aq. The mixture was stirred overnight and extracted with $CHCl_3$ three times. The organic phase was dried with Na_2SO_4 and filtered through a plug of a celite to remove insoluble materials and Na_2SO_4. The resultant solution was concentrated under reduced pressure and precipitated into MeOH. The precipitation was washed overnight in a Soxhlet apparatus using MeOH and acetone to remove oligomers and catalyst residues. After that, the residual solid was extracted with chloroform in a Soxhlet apparatus. The solvent was evaporated, and the dark yellow solid was obtained (3.50 g, 77% yield). ^{1}H NMR (500.13 MHz, $CDCl_3$, ppm): δ = 7.83 (br, Ar*H*), 7.68 (br, Ar*H*), 2.12 (br, $\mathit{CH_2}(CH_2)_5CH_2CH_3$), 1.20–1.14 (br, $CH_2(\mathit{CH_2})_5CH_2CH_3$), 0.82 (br, $CH_2(CH_2)_5\mathit{CH_2CH_3}$). ^{13}C NMR (125.76 MHz, $CDCl_3$, ppm): 151.9, 140.6, 140.1, 126.3, 121.6, 120.1, 55.5, 40,5, 31.9, 30.2, 29.4, 29.4, 24.0, 22.8, 14.2. M_n = 14900 g mol^{-1}, M_w = 47000 g mol^{-1}, $Đ$ = 3.2 (GPC relative to polystyrene standards).

PFO with high molecular weight (M_n = 54700 g mol^{-1}, M_w = 198000 g mol^{-1}, $Đ$ = 3.6) was also prepared via Suzuki–Miyaura coupling polymerization. The reagents and solvent loading were changed as follows: 9,9-dioctylfluorene-2,7-diboronic acid bis(1,3-propanediol) ester (837.6 mg, 1.5 mmol), 2,7-dibromo-9,9-dioctylfluorene (822.3 mg, 1.5 mmol), $Pd(PPh_3)_4$ (43.6 mg, 0.038 mmol), toluene (30 mL), and 2 M K_2CO_3 aq. (20 mL). Another key modification was the purification procedure. In the Soxhlet extraction process, the crude was sequentially washed with MeOH, acetone, and hexane. After that, the residual solid was extracted with chloroform in a Soxhlet apparatus. After the evaporation of the solvent, a dark yellow solid was obtained (114.2 mg, 10% yield).

PFO with low molecular weight (M_n = 8600 g mol^{-1}, M_w = 14002 g mol^{-1}, $Đ$ = 1.7) was also prepared via Suzuki–Miyaura coupling polymerization. The reagents and solvent loading were changed as follows: 9,9-dioctylfluorene-2,7-diboronic acid bis(1,3-propanediol) ester (838.9 mg, 1.5 mmol), 2,7-dibromo-9,9-dioctylfluorene (823.9 mg, 1.5

mmol), $Pd(PPh_3)_4$ (43.4 mg, 0.038 mmol), THF (15 mL), and K_2CO_3 aq. (10 mL). Another key modification was the purification procedure. In the Soxhlet extraction process, the crude was sequentially washed with MeOH, acetone. After that, the residual solid was extracted with Hexane in a Soxhlet apparatus. After the evaporation of the solvent, a yellow solid was obtained (200.2 mg, 17% yield).

*Synthesis of poly(9-(9-heptadecyl)dioctylcarbazole) PCD (**Pre6**)*

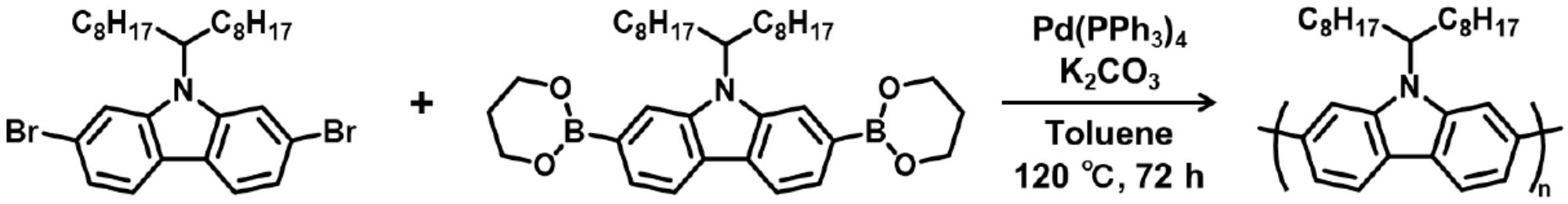

PCD was synthesized according to the literature.[48] 2,7-Dibromo-9-(9-heptadecyl)carbazole (563.5 mg, 1.00 mmol), 9-(9-heptadecanyl)-2,7-bis(4,4,5,5-tetramethyl-1,3,2-dioxaborolan-2-yl)carbazole (657.6 mg, 1.00 mmol), $Pd(PPh_3)_4$ (26.4 mg, 0.023 mmol) were loaded into a Schlenk flask. The flask was vacuumed for 30 min and replaced with Ar gas, and then anhydrous toluene (10 mL), and degassed 2M K_2CO_3 aq. (6 mL) were added. The mixture was stirred under reflux for 72 h. The whole mixture was diluted with $CHCl_3$, and the chloroform solution was poured into a large amount of 0.1 M EDTA·4Na aq. The mixture was stirred overnight and extracted with $CHCl_3$ three times. The organic phase was dried with Na_2SO_4 and filtered through a plug of a celite to remove insoluble materials and Na_2SO_4. The resultant solution was concentrated under reduced pressure and precipitated into MeOH. The precipitation was washed overnight in a Soxhlet apparatus using MeOH and acetone to remove oligomers and catalyst residues. After that, the residual solid was extracted with chloroform in a Soxhlet apparatus. The solvent was evaporated, and the dark gray solid was obtained (609.3 mg, 76%). ^{1}H NMR (500.13 MHz, $CDCl_3$, ppm): δ = 8.25 (br, Ar*H*), 7.93 (br, Ar*H*), 7.75 (br, Ar*H*), 7.63 (br, Ar*H*), 4.77 (br, $NC\mathit{H}((CH_2)_7CH_3)_2$), 2.46 (br, $NCH((C\mathit{H}_2(CH_2)_6CH_3)_2$), 2.06 (br, $NCH((C\mathit{H}_2(CH_2)_6CH_3)_2$), 1.32–1.18 (br, $NCH((CH_2(C\mathit{H}_2)_6CH_3)_2$), 0.81 (t, $NCH((CH_2(CH_2)_6C\mathit{H}_3)_2$). ^{13}C NMR (125.76 MHz, $CDCl_3$, ppm): 143.1, 140.4, 139.9, 122.9, 121.5, 121.5, 120.6, 120.3, 119.1, 110.8, 108.0, 56.5, 31.8, 29.6, 29.4, 29.3, 27.0, 22.6, 14.1. $M_n = 20300$ g mol^{-1}, $M_w = 70800$ g mol^{-1}, $Đ = 3.5$ (GPC relative to polystyrene standards).

*Synthesis of fluorene-thiophene alternating copolymer (**Pre7**)*

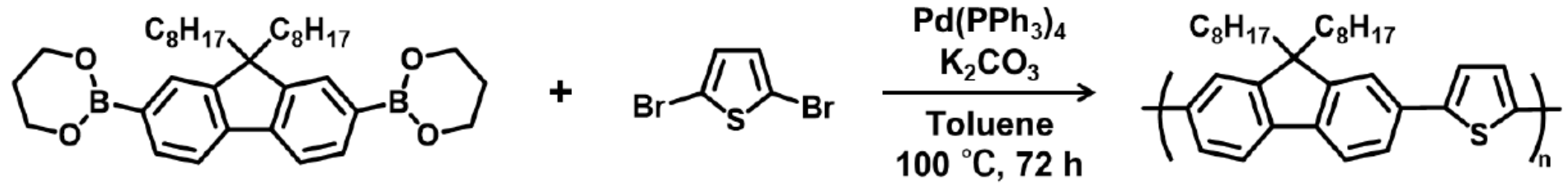

P(Fl-Th) was synthesized according to the literature.[22,49] 9,9-Dioctylfluorene-2,7-diboronic acid bis(1,3-propanediol) ester (555.7 mg, 1.00 mmol) and $Pd(PPh_3)_4$ (11.6 mg, 0.010 mmol) were loaded into a Schlenk flask. The flask was vacuumed for 30 min and replaced with argon, and then anhydrous toluene (5 mL), 2,5-dibromo thiophene (239.7 mg,0.99 mmol), and degassed 2M K_2CO_3 aq. (3 mL) were added. The mixture was stirred under reflux for 72 h. The whole mixture was diluted with $CHCl_3$, and the chloroform solution was poured into a large amount of 0.1 M EDTA·4Na aq. The mixture was stirred overnight and extracted with $CHCl_3$ three times. The organic phase was dried with Na_2SO_4 and filtered through a plug of celite to remove insoluble materials and Na_2SO_4. The resultant solution was concentrated under reduced pressure and precipitated into MeOH. The precipitation was washed overnight in a Soxhlet apparatus using acetone to remove oligomers and catalyst residues. After that, the residual solid was extracted with chloroform in a Soxhlet apparatus. The solvent was evaporated, and the yellow solid was obtained (349.1 mg, 74%). ^{1}H NMR (500.13 MHz, $CDCl_3$, ppm): δ = 7.62 (br, Ar*H*), 7.42 (br, Ar*H*), 2.08 (br, $\mathit{CH_2}(CH_2)_5CH_2CH_3$), 1.19–1.10 (br, $CH_2(\mathit{CH_2})_5CH_2CH_3$), 0.89 (br, $CH_2(CH_2)_5\mathit{CH_2CH_3}$). ^{13}C NMR (125.76 MHz, $CDCl_3$, ppm): 151.9, 144.3, 140.4, 133.4, 124.8, 124.1, 120.3, 120.0, 55.5, 40,6, 32.0, 30.2, 29.4, 23.9, 22.8, 14.2. M_n = 4600 g mol^{-1}, M_w = 17700 g mol^{-1}, $Đ$ = 3.9 (GPC relative to polystyrene standards).

*Synthesis of poly((9,9-dioctylfluorene)-(ethylenedioxythiophene)) P(Fl-EDOT) (**Pre8**)*

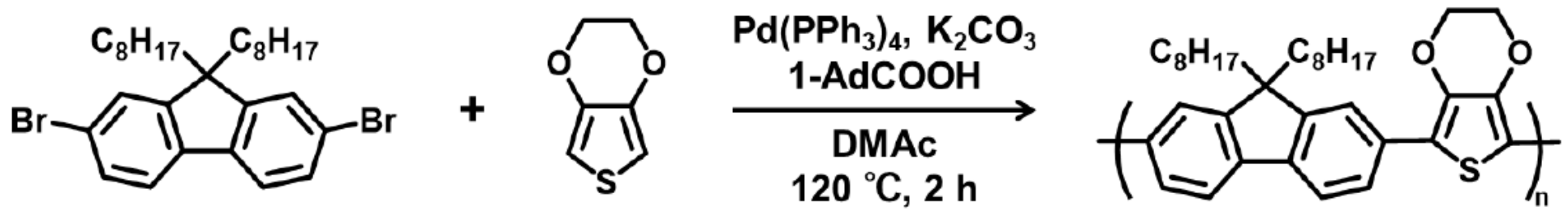

P(Fl-EDOT) was synthesized according to the literature.[22,49] 2,7-Dibromo-9,9-dioctylfluorene (507 mg, 0.92 mmol), EDOT (129 mg, 0.92 mmol), $PdCl_2$ (3 mg, 17.0 μmol), 1-AdCOOH (48 mg, 0.25 mmol), and K_2CO_3 (382 mg, 2.76 mmol) were loaded into a Schlenk flask. The flask was vacuumed for 30 min, and then replaced with Ar gas. Anhydrous DMAc (3 mL) was added into the flask, and then the mixture was stirred under reflux for 2 h. After the reaction, the whole mixture was diluted with $CHCl_3$, and chloroform solution was poured into a large amount of 0.1 M EDTA·4Na aq. The mixture was stirred overnight and extracted with $CHCl_3$ three times. The organic phase was washed with 1M HCl aq. three times and H_2O, brine, and then the organic phase was dried with Na_2SO_4 and filtered through a plug of celite to remove insoluble material and Na_2SO_4. The resultant solution was concentrated under reduced pressure and precipitated into MeOH. The precipitation was collected with the filtration through a Soxhlet thimble, extracted with MeOH, and $CHCl_3$. The $CHCl_3$ solution was concentrated under reduced pressure, and precipitated with MeOH, and the yellow solid was obtained (329 mg, 67%). ^{1}H NMR (500.13 MHz, $CDCl_3$, ppm): δ = 7.83 (br, Ar*H*), 7.70 (br, Ar*H*), 4.45 (br, O*(CH₂)₂*O), 2.06 (br, $\mathit{CH_2}(CH_2)_5CH_2CH_3$), 1.21–1.11 (br, $CH_2(\mathit{CH_2})_5CH_2CH_3$), 0.81 (br, $CH_2(CH_2)_5\mathit{CH_2CH_3}$). ^{13}C NMR (125.76 MHz, $CDCl_3$, ppm): 151.6, 139.6, 138.8, 131.9, 125.2, 120.5, 119.9, 116.2, 64.8 55.4, 40,6, 32.0, 30.2, 29.4, 23.9, 22.8, 14.2. M_n = 82400 g mol^{-1}, M_w = 474800 g mol^{-1}, PDI = 5.8 (GPC relative to polystyrene standards).

*Synthesis of poly((9,9-dioctylfluorene)-(ethylenedioxythiophene)) P(Fl-F4) (**Pre9**)*

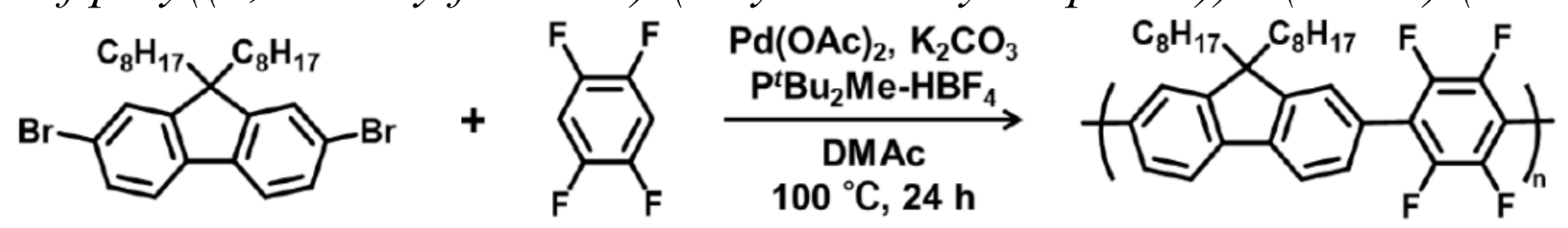

P(Fl-F4) was synthesized according to the literature.[49,50] 2,7-Dibromo-9,9-dioctylfluorene (548 mg, 2.0 mmol), $Pd(OAc)_2$ (11.2 mg, 50 μmol), $P^tBu_2Me-HBF_4$ (24.8 mg, 0.10 mmol), and K_2CO_3 (276 mg, 4.0 mmol) were loaded into a Schlenk flask. The flask was vacuumed for 30 min, and then replaced with Ar gas. After that, 1,2,4,5-tetrafluorobenzene (0.112 mL, 150.1 mg, 1.0 mmol) and anhydrous DMAc (1.0 mL) was added into the flask, and then the mixture was stirred under reflux at 100°C for 24 h. After the reaction, the whole mixture was poured into a large amount of 0.1 M EDTA·4Na aq. The mixture was stirred overnight and extracted with $CHCl_3$ three times. The organic phase was washed with a saturated aqueous solution of NaCl, and then the organic phase was dried with Na_2SO_4 and filtered through a plug of celite to remove insoluble material and Na_2SO_4. The resultant solution was concentrated under reduced pressure and precipitated into MeOH, and the gray solid was obtained (480 mg, 90%). ^{1}H NMR (500.13 MHz, $CDCl_3$, ppm): δ = 7.92–7.91 (br, Ar*H*), 7.57 (br, Ar*H*), 2.05 (br, $\mathit{CH_2}(CH_2)_5CH_2CH_3$), 1.23–1.12 (br, $CH_2(\mathit{CH_2})_5CH_2CH_3$), 0.84–0.81 (br, $CH_2(CH_2)_5\mathit{CH_2CH_3}$). ^{19}F NMR (470.59 MHz, $CDCl_3$, ppm): −144.12 (Ar-F). M_n = 43100 g mol^{-1}, M_w = 284300 g mol^{-1}, PDI = 6.6 (GPC relative to polystyrene standards).

Synthesis of 2,7-bis(4-(tert-butyl)phenyl)-9,9-dimethyl-9Hfluorene (BPF)

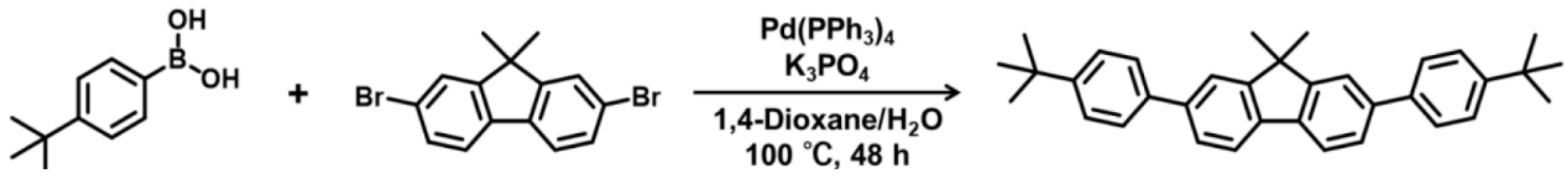

BPF was synthesized according to the literature.[51,52] 9, 9-Dimethyl-2, 7-dibromofluoren (2.11 g, 6.0 mmol), potassium phosphate (2.55 g, 48 mmol), 4-tert-butylbenzeneboronic acid (2.22 g, 12.5 mmol), terakis(triphenylphosphine)palladium (550 mg, 0.23 mmol), were loaded into a 100mL two-neck flask. The flask was vacuumed for 30 min, and then replaced with Ar gas. Anhydrous dioxane (30 mL) and distilled water (6 mL) were added into the flask, and then the mixture was stirred under reflux for 48 h. After the reaction, the mixture was diluted with $CHCl_3$, and the chloroform solution was washed with brine three times. The organic solution was dried with Na_2SO_4 and filtered through a plug of a celite to remove insoluble materials and Na_2SO_4. The resultant solution was concentrated under reduced pressure, and then, the residual solid was washed with toluene till the solid became white (1.15 g, 42%). ^{1}H NMR (500.13 MHz, $CDCl_3$, ppm): δ = 7.77 (d, J = 7.85 Hz, 2H), 7.64 (d, J = 1.25 Hz, 2H), 7.61 (m, 4H), 7.57 (dd, J = 7.85 Hz, J = 1.65 Hz, 2H), 7.49 (m, 4H), 1.56 (s, 6H), 1.38 (s, 18H). ^{13}C NMR (125.76 MHz, $CDCl_3$, ppm): 154.6, 150.3 140.3, 138.9, 138.1 127.0, 126.2, 125.9, 121.5, 120.4, 47.1, 34.7, 31.5, 27.5.

4. General procedures for photochemical phosphonylation of PFO

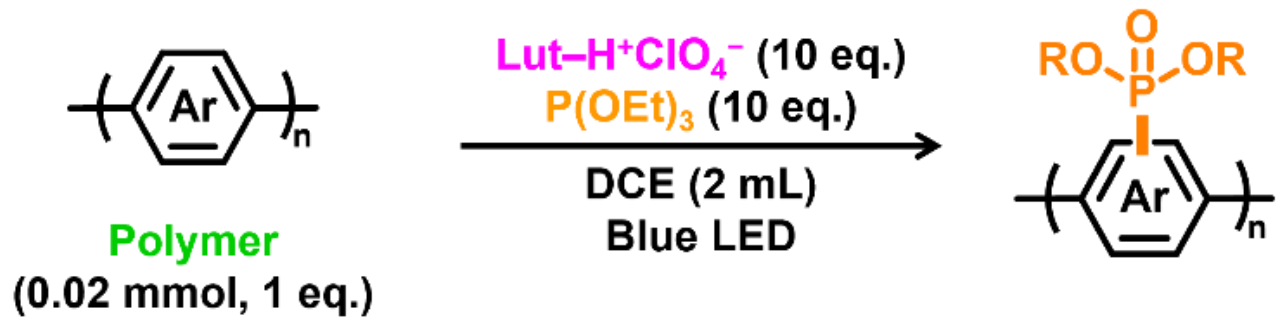

Polymer (0.02 mmol monomer-unit, 1.0 eq.), Lut–$H^+ClO_4^-$(0.2 mmol, 10 eq.) were added to a 4 mL Mighty Vial (manufactured by Maruemu Corporation), and then, anhydrous DCE (2.0 mL) was loaded, and the mixture was stirred until the polymer was perfectly dissolved. After that, $P(OR)_3$ (0.2 mmol, 10 eq.) was added. The mixture was bubbled with Ar for approximately 5 min with cooling at 0 °C. Two blue LEDs (Kessil A160 WE Tuna Blue) were set to maximum wavelength and intensity, and irradiation was carried out. A fan (Sanwa Supply VEHFAN1) was used for cooling during the process. To block light and dissipate heat, the entire apparatus was covered with an absorbent roll (As One Corporation, liquid absorbent roll for water, oil, and solvents, GAR75). Upon completion of the reaction, the mixture was concentrated and dried under vacuum at 60 °C. The crude solid was then washed with methanol until the complete removal of $P(OEt)_3$ and lutidinium salts was confirmed by NMR spectroscopy.

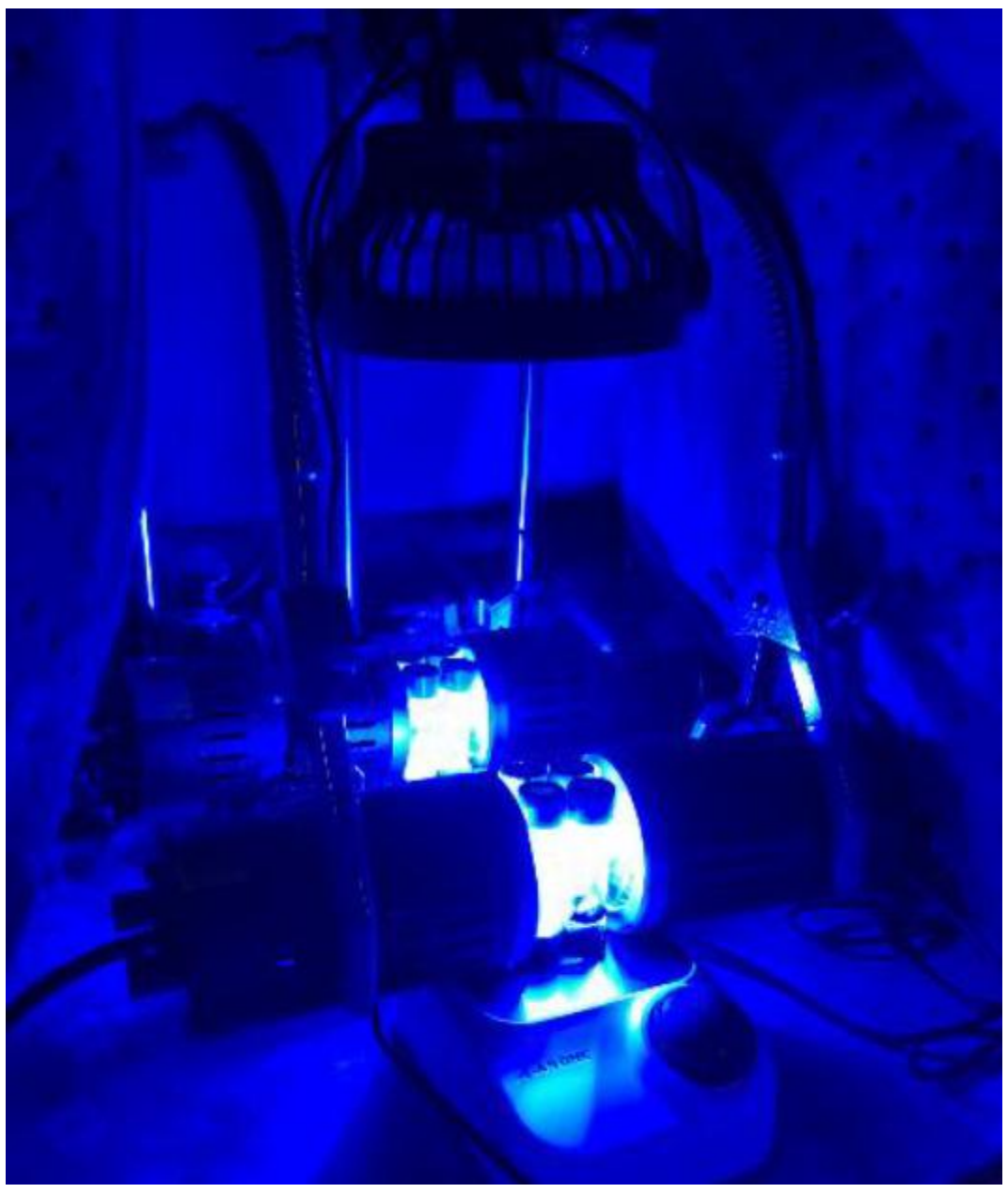

Figure S1 . Setup for light irradiation experiment.

5. **The study of NMR and IR spectra of PFO-Phos(Et)**

According to the reference,[39] the phosphonylation site can be selectively identified by ^1H NMR spectra. For example, in a model fluorene compound, the α-methylene resonance of the phosphonate at the 4-position of the fluorene ring appears at 3.8–4.0 ppm, whereas that at the 3-position appears at 4.0–4.2 ppm (Figure S2). In addition, the 4-position phosphonylated product exhibits new aromatic signals at 8.2 and 8.6 ppm, while the 3-position product shows a signal at 8.4 ppm.

Similarly, in this work, the corresponding broad signals of 3- and 4-position phosphonylation were obtained (Figure S2). The ratio of phosphonylation at each position was determined by comparing the integral of α-methylene signals of phosphonate.

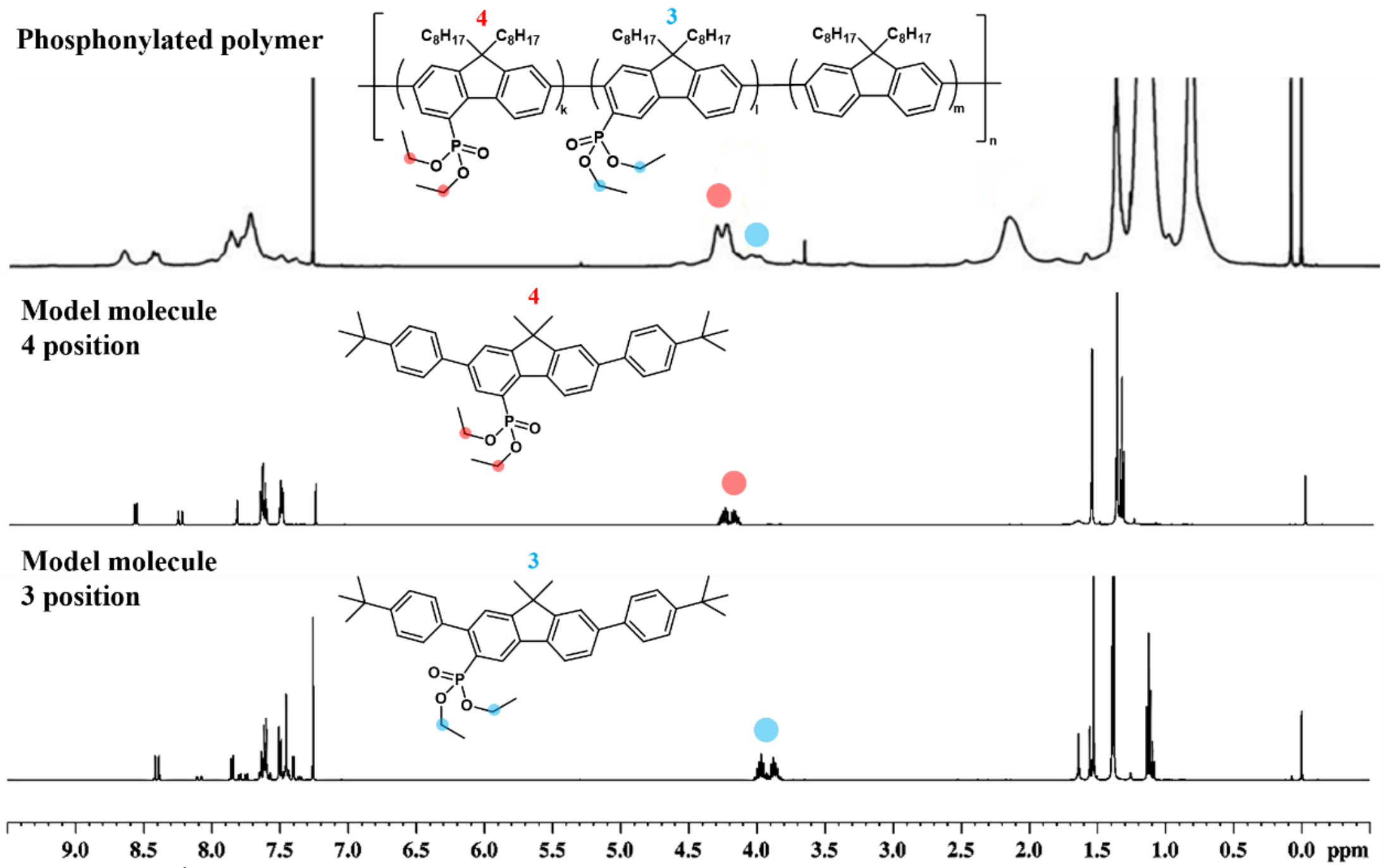

Figure S2. ^1H NMR spectra of phosphonylated polymer and model molecules.

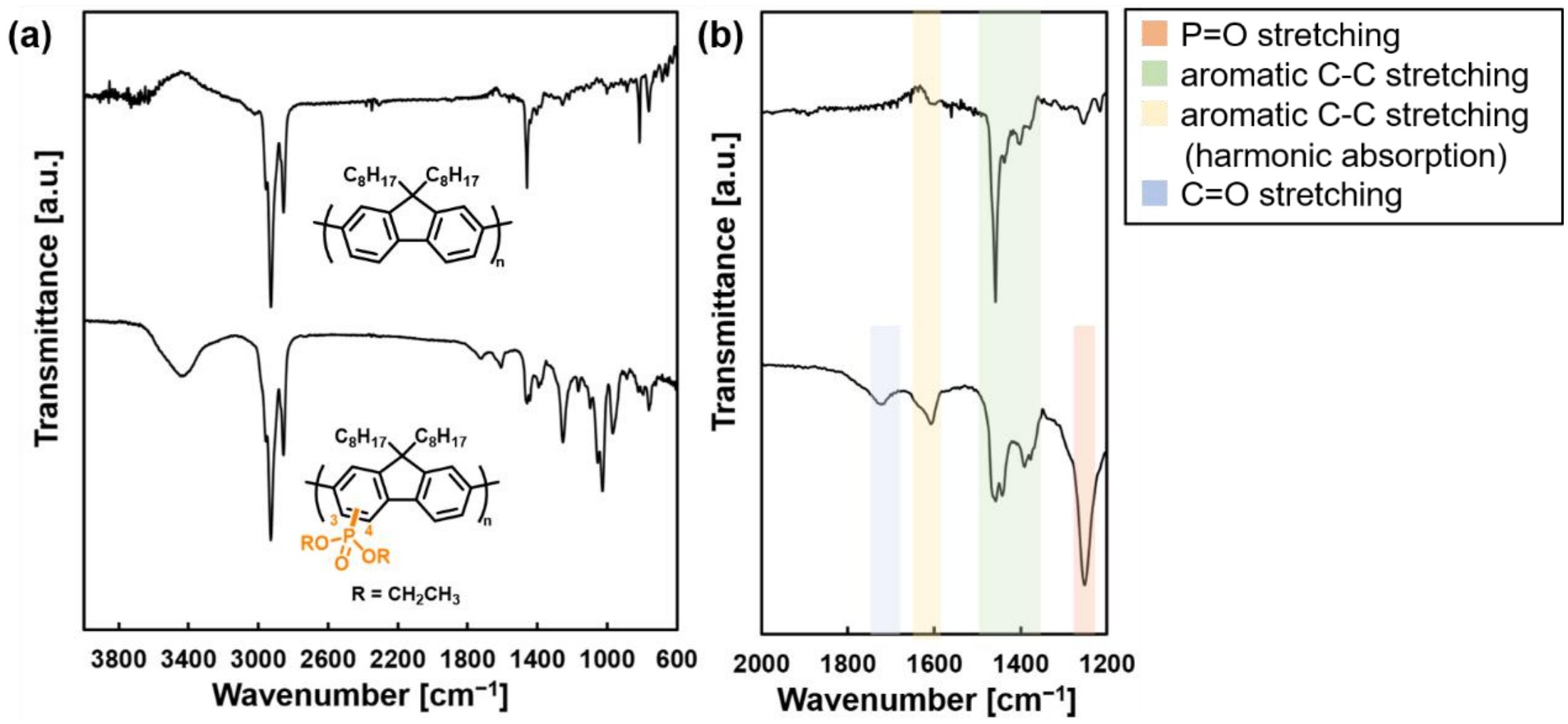

Figure S3. FT-IR spectra of PFO and phosphonylated PFO. (a) Whole wavenumber range and (b) magnified in range of 1200–2000 cm^{-1}.

Table S1 Comparison of DOF calculation method.

	DOF calculated from alkyl-CH_2 [%]	DOF based on aromatic H [%]
PFO Sample (1 h)	22	18
PFO Sample (2 h)	75	67
PFO Sample (3 h)	96	96
PFO Sample (5 h)	98	96

To investigate the degradation, the IR spectra of reactant PFO and PFO-Phos(Et) (DOF = 100%) were measured (Figure S3). The absorption band derived from C=O stretching was observed in the spectrum of PFO-Phos(Et). Since its absorption strength was relatively low (11% of P=O peak), the calculated DOF should not be significantly affected by such degradation. To support this, DOF was calculated only based on aromatic proton signals at 8.65 ppm for 4-substitutuion, at 8.53 ppm for 3-substituion, and the others (7.3–8.2 ppm). The results and comparison with previous method are summarized in Table S1. The difference of the DOF values is not large.

6. UV-vis absorption spectra and Stern–Volmer analysis

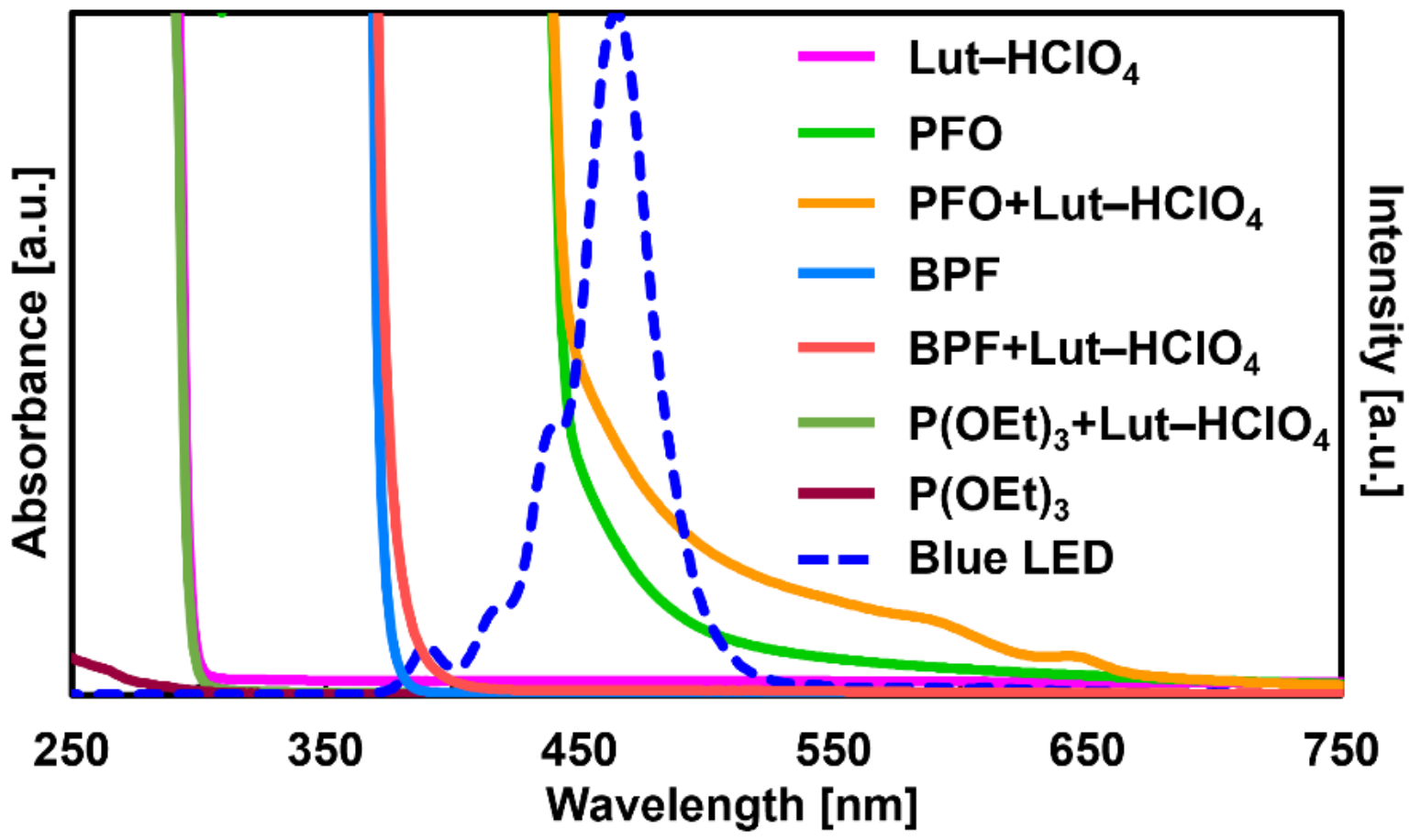

Figure S4.UV-vis spectra of PFO (green line), Lut–$H^+ClO_4^-$ (pink line), PFO+Lut–$H^+ClO_4^-$ (orange line), BPF (blue line), BPF+Lut–$H^+ClO_4^-$ (red line), $P(OEt)_3$ (brown line), and $P(OEt)_3$+Lut–$H^+ClO_4^-$ (light green line). UV-vis spectra of the reaction mixture were measured under identical concentration to the reaction conditions [PFO (0.02 mmol), BPF (0.02 mmol), $P(OEt)_3$ (0.2 mmol), and Lut–$H^+ClO_4^-$ (0.2 mmol) in DCE (2 mL) solution]. Emission spectrum of the used blue light LED (navy dot line).

For PFO, the molar absorption coefficient (ε) was determined to be 2.7 at 387 nm, whereas for BPF, it was 40.2 at 350 nm (the 350 nm excitation for BPF was selected due to the lower limit of our spectrometer). As shown in Figure S5, the quenching efficiency observed for PFO is significantly higher than that for BPF (Figure S4 ef). Furthermore, we investigated the changes in fluorescence quantum yield (Φ). Upon the addition of 12 mmol L^{-1} lutidinium, the quantum yield of PFO decreased substantially from 0.39 to 0.18. In contrast, the quantum yield of BPF only exhibited a minor decrease from 0.76 to 0.66 under identical conditions. This pronounced degree of quenching in PFO supports enhanced reactivity to the conjugated polymer system.

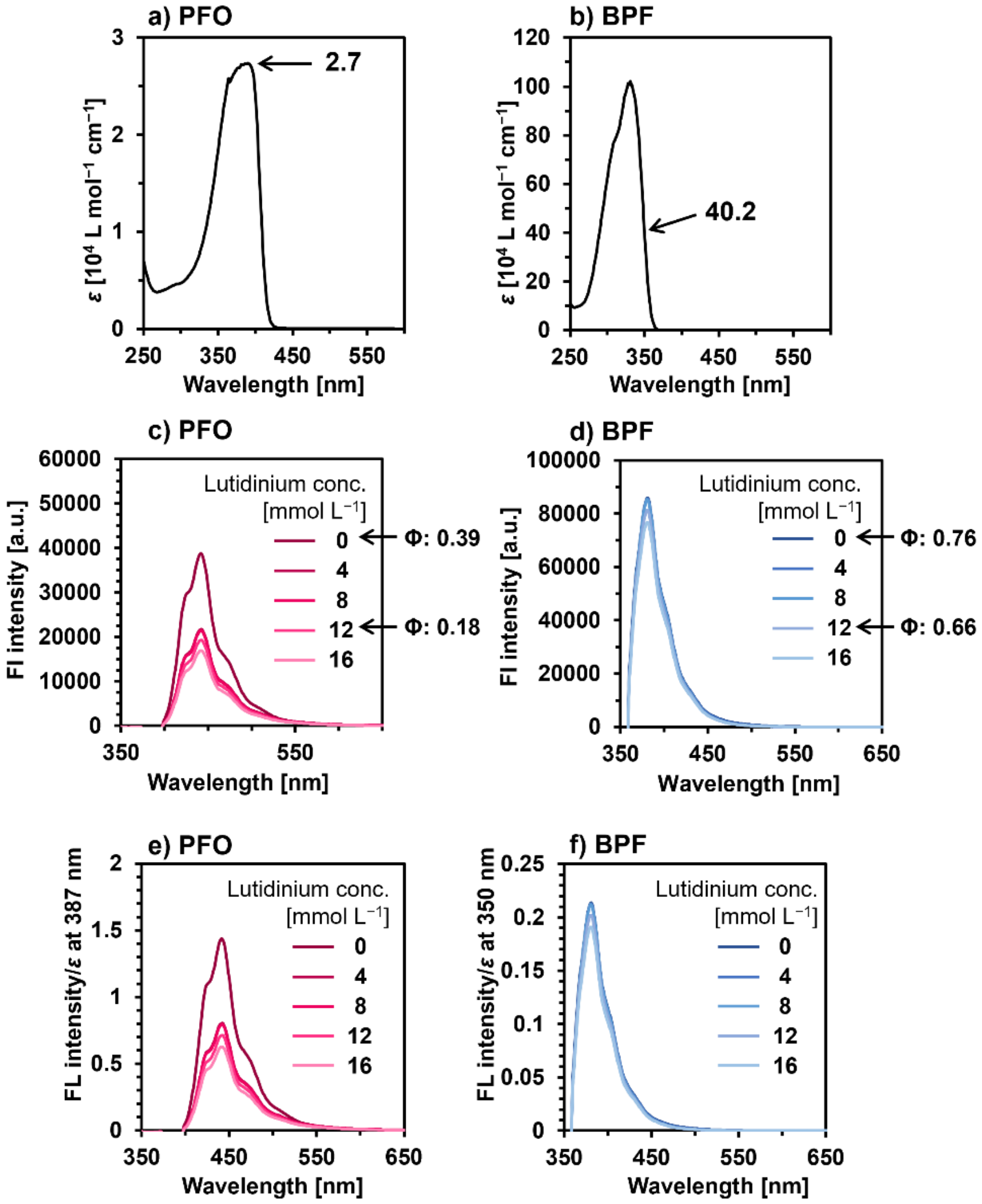

Figure S5. UV-Vis absorption spectra of (a) 1.0×10^{-4} mol L^{-1} PFO and (b) 2.5×10^{-6} mol L^{-1} BPF measured in DCE. Fluorescence spectra of 1.0×10^{-3} mol L^{-1} (c) PFO and (d) BPF measured in DCE. Fluorescence intensity was normalized to the molar absorbance coefficient at (e) 387 nm of the PFO spectrum and (f) 350 nm of the BPF spectrum.

7. The relationship between the reaction time and DOF

To optimize the reaction time, we conducted the reaction in triplicate with several reaction times. DOF gradually increased with the reaction time and reached a maximum at 3 hours (Table S2, Figure S6 and S7). On the other hand, the DOF was identical after 3 hours. These results indicate that DOF is controllable through reaction time up to 3 hours. Positional selectivity was unchanged with respect to the reaction time.

Interestingly, the reaction rate remained steady until nearly quantitative conversion. Despite the polymeric nature, the reaction proceeded smoothly to completion within 3 hours, highlighting the high efficiency and promise of this method for CP functionalization.

Table S2. DOF of the C−H phosphonylation versus reaction time.

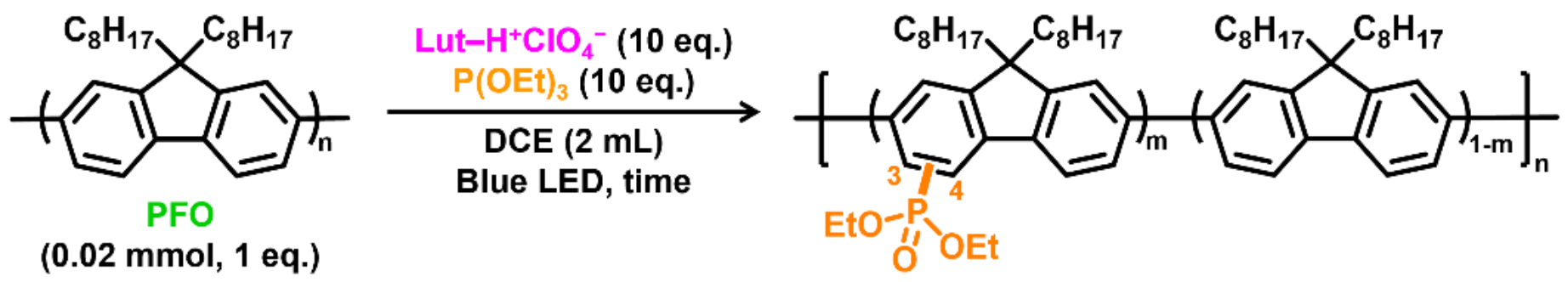

Time	DOF(%)[a]			Selectivity (4-Phos)
	3	4	3 + 4	
1 h	3	19	22	87%
2 h	11	64	75	85%
3 h	24	72	96	75%
4 h	23	77	98	78%
5 h	24	75	99	75%

[a] Degree of functionalization was determined by ^{1}H NMR.

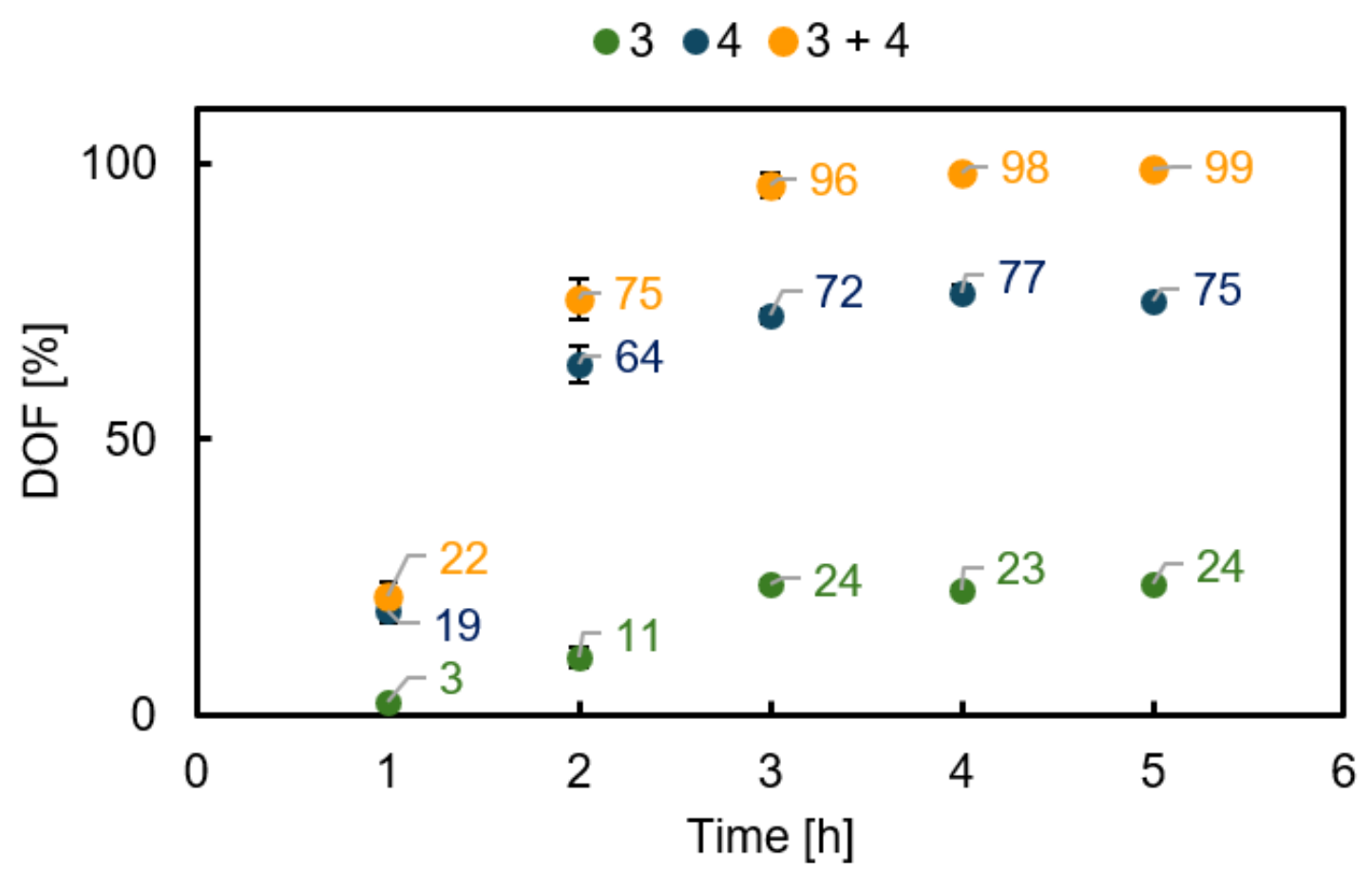

Figure S6. DOF as a function of reaction time for the photochemical phosphonylation of PFO. Data represent the average of three independent reactions with error bars indicating standard deviation.

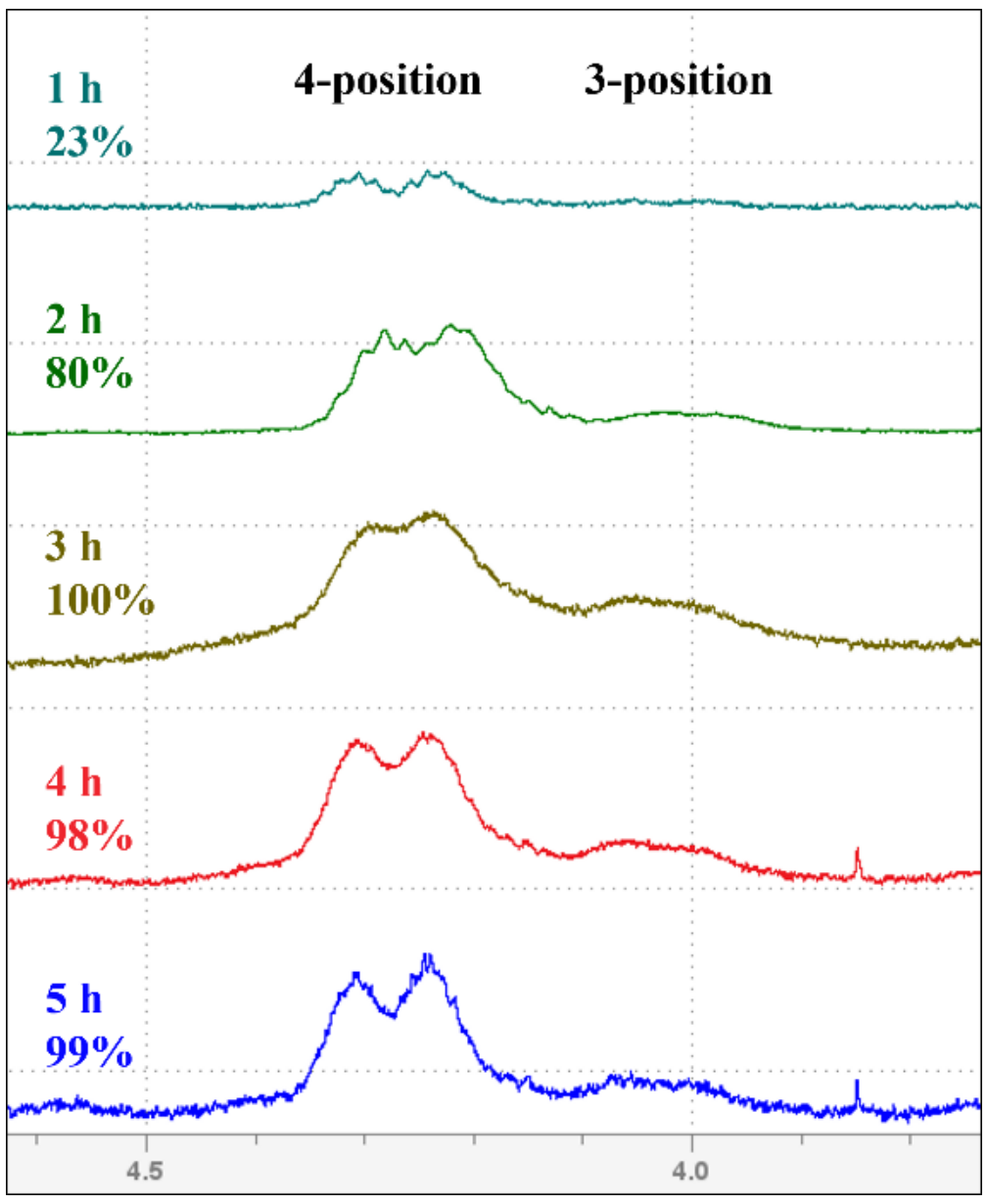

Figure S7. Temporal variation of NMR spectra (derived from a phosphonyl group)

8. Sunlight reaction of PFO

The reaction under sunlight was conducted under the condition shown in Figure S8, providing a DOF of 16%. Compared with the standard light-irradiation condition, the DOF decreased and the reaction time was prolonged, probably because of the lower intensity of sunlight. Nevertheless, this result demonstrates that sunlight can potentially drive EDA-complex excitation-induced polymer reaction.

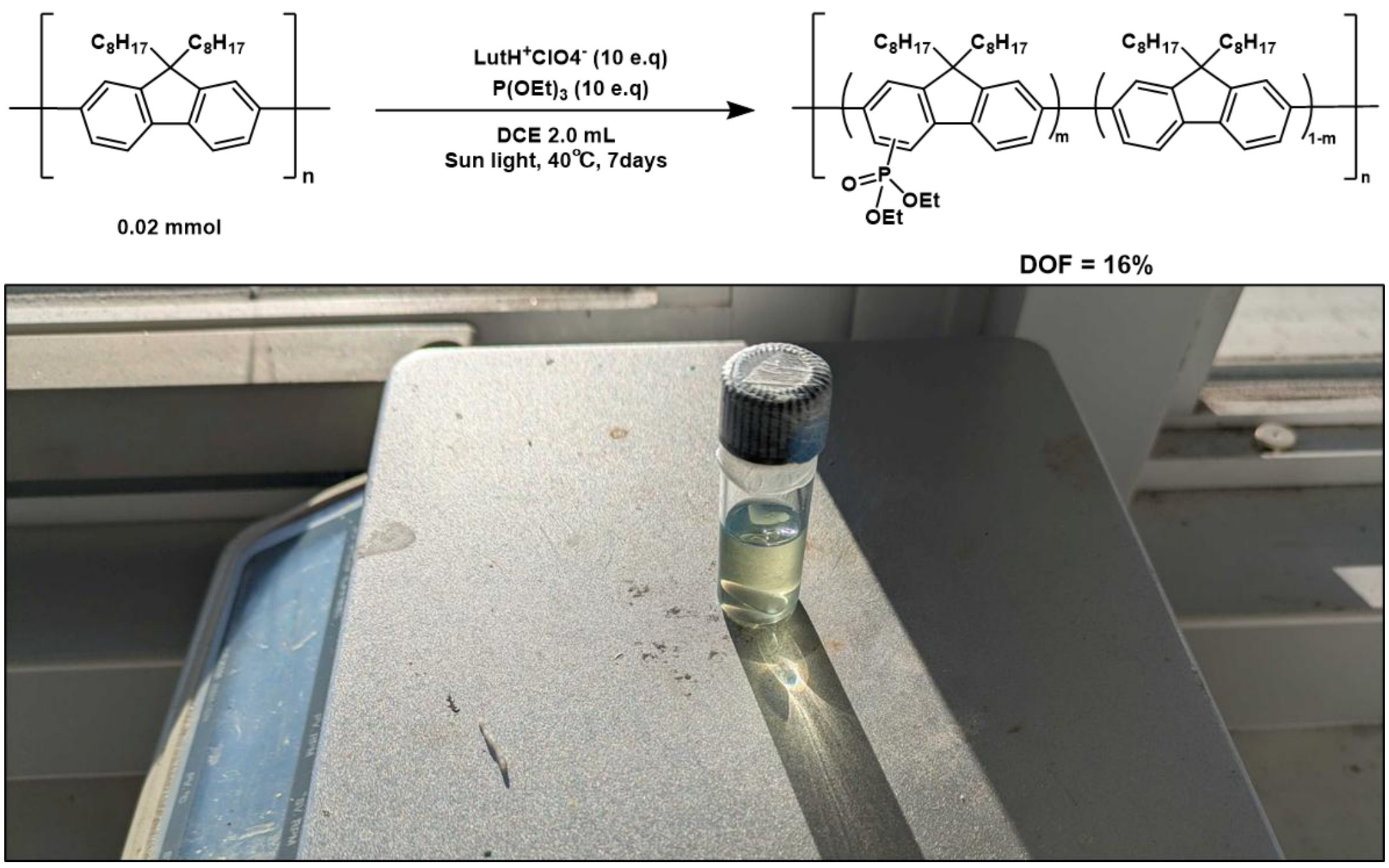

Figure S8. Photographs of photochemical reaction of PFO under sun light(DOF: 16%).

9. Investigation of NMR spectra of P(Fl-Th) and P(Fl-Th-Phos(Et))

Figure S9 shows the proton signals of P(Fl-Th) and P(Fl-Th-Phos(Et)). The proton signal of thiophene rings almost disappeared, indicating that phosphonate was introduced at thiophene, and the aromatic proton signal of phosphonylated thiophene shifted toward downfield. For example, the chemical shift of aromatic proton in diethyl (2,5-dimethylphenyl) phosphonate (δ = 7.75 ppm ($CDCl_3$))[15],[53] is located downfield, compared to that in xylene (δ = 7.06 ppm ($CDCl_3$)).[54] Moreover, the ^{31}P NMR spectrum of P(Fl-Th-Phos(Et)) is shown in Figure S40. The signal observed around 13.5 ppm is close to the chemical shift of diethyl(thiophene-3-yl)phosphonate (δ = 13.6 ppm).[55]

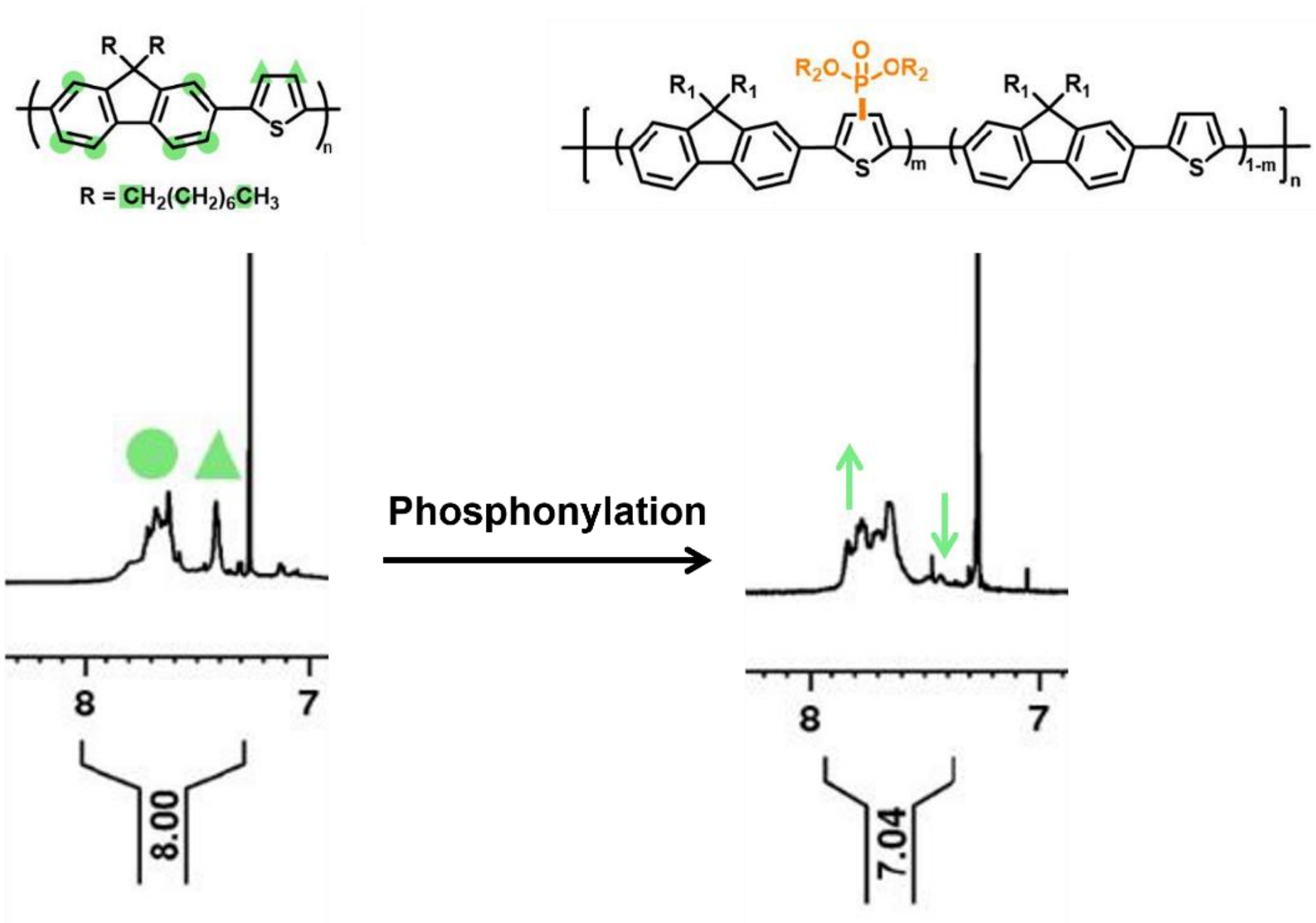

Figure S9. ^1H NMR of P(Fl-Th) and P(Fl-Th-Phos(Et)) (DOF: 100%) enlarged the aromatic region.

10. Calculation of dihedral angles by DFT calculation

To evaluate the structural effects of the phosphonate moieties, DFT calculation was conducted (Figure S10). Phenyl-fluorene (PF), 4-position phosphonylated phenyl fluorene (4P-PF), and 3-position phenyl fluorene (3P-PF) were used as models for PFO, 4-phosphonylated PFO, and 3-phosphonylated PFO, respectively. The calculated dihedral angles between the adjacent benzene and fluorene units were 40.9° in PF, 37.7° in 4P-PF, and 63.0° in 3P-PF (Figure S7). These results indicate that the introduction of diethyl phosphonate at the 4-position of the fluorene ring has no impact for the main chains twist.

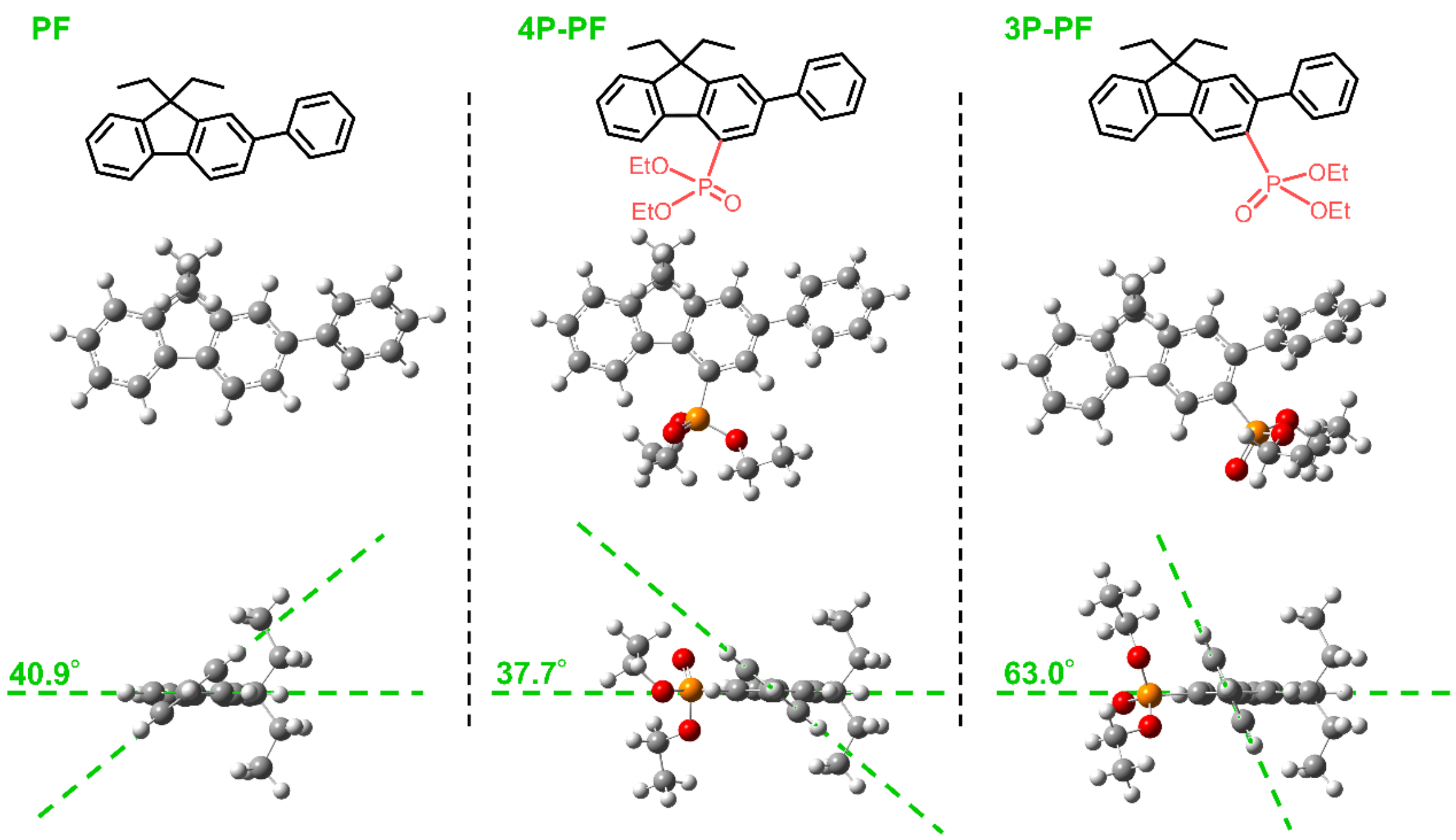

Figure S10. Molecular structures (upper), front view (middle), and side view (lower) of the PF, 4P-PF, and 3P-PF. DFT calculation was performed at the PCM-ωB97XD/6-311G+(d,p)//PCM-ωB97XD/6-31G+(d,p) level of theory using Gaussian 16.

11. TD DFT calculation

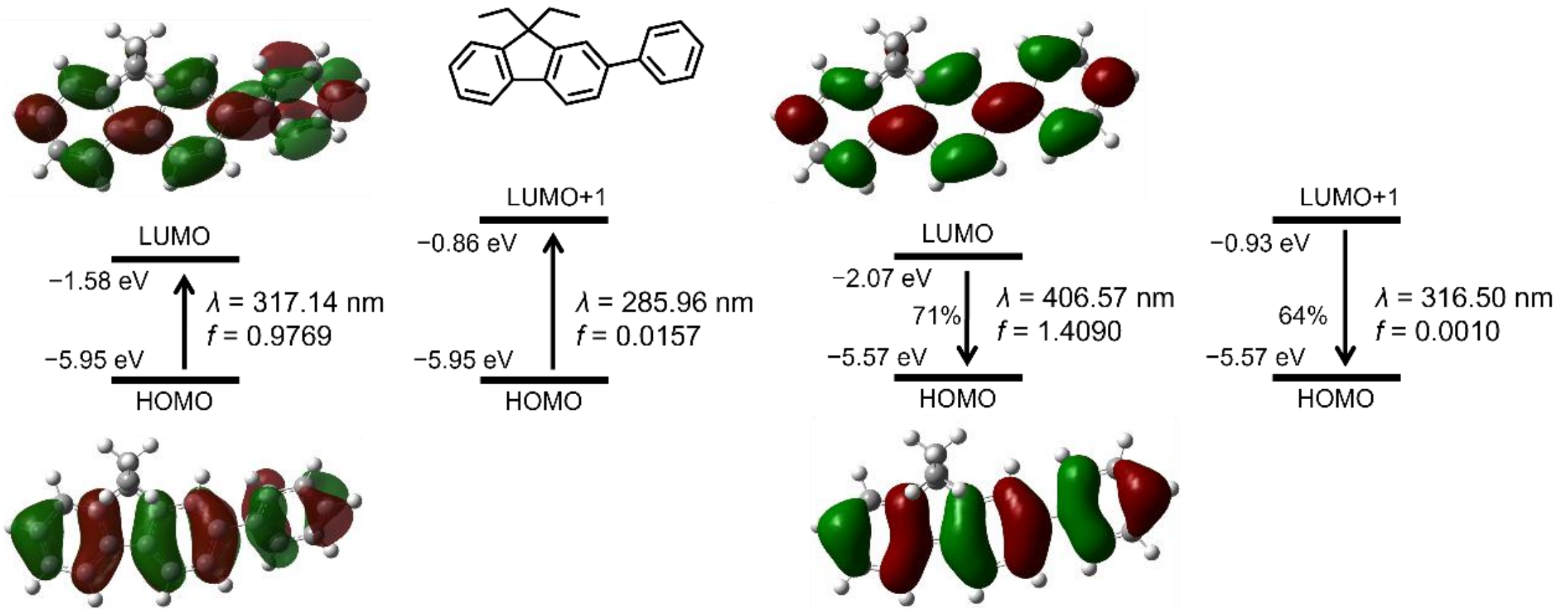

Figure S11. Orbital correlation and composition of the $S_0 \rightarrow S_1$ (left side) and the $S_1 \rightarrow S_0$ (right side) transition for PF calculated at B3LYP/6-311G+(d, p) level basis set. Molecular orbitals of HOMO and LUMO are described (iso value = 0.02). λ and f mean wavelength and oscillator strength, respectively.

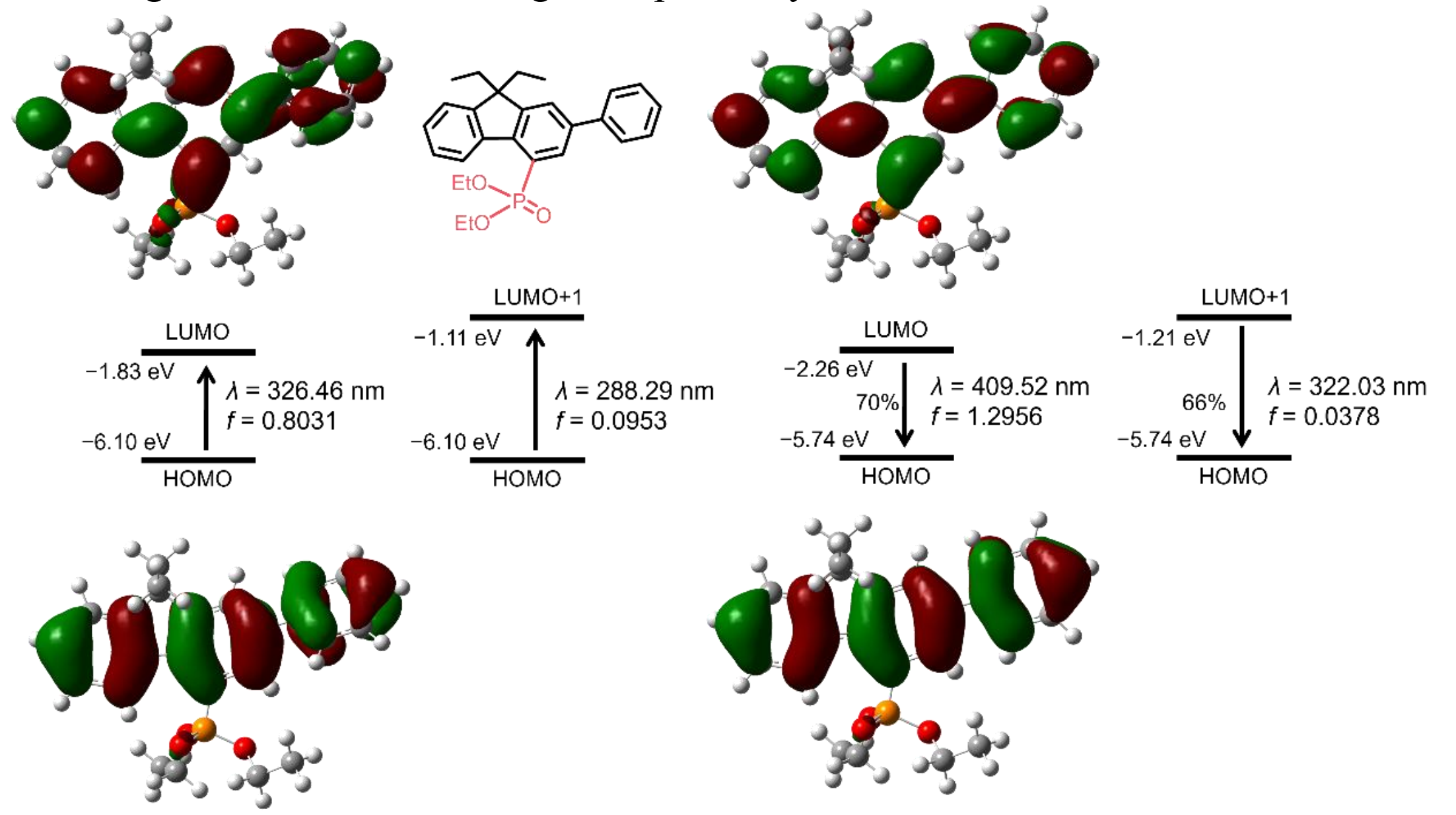

Figure S12. Orbital correlation and composition of the $S_0 \rightarrow S_1$ (left side) and the $S_1 \rightarrow S_0$ (right side) transition for 4P-PF calculated at B3LYP/6-311G+(d, p) level basis set. Molecular orbitals of HOMO and LUMO are described (iso value = 0.02). λ and f mean wavelength and oscillator strength, respectively.

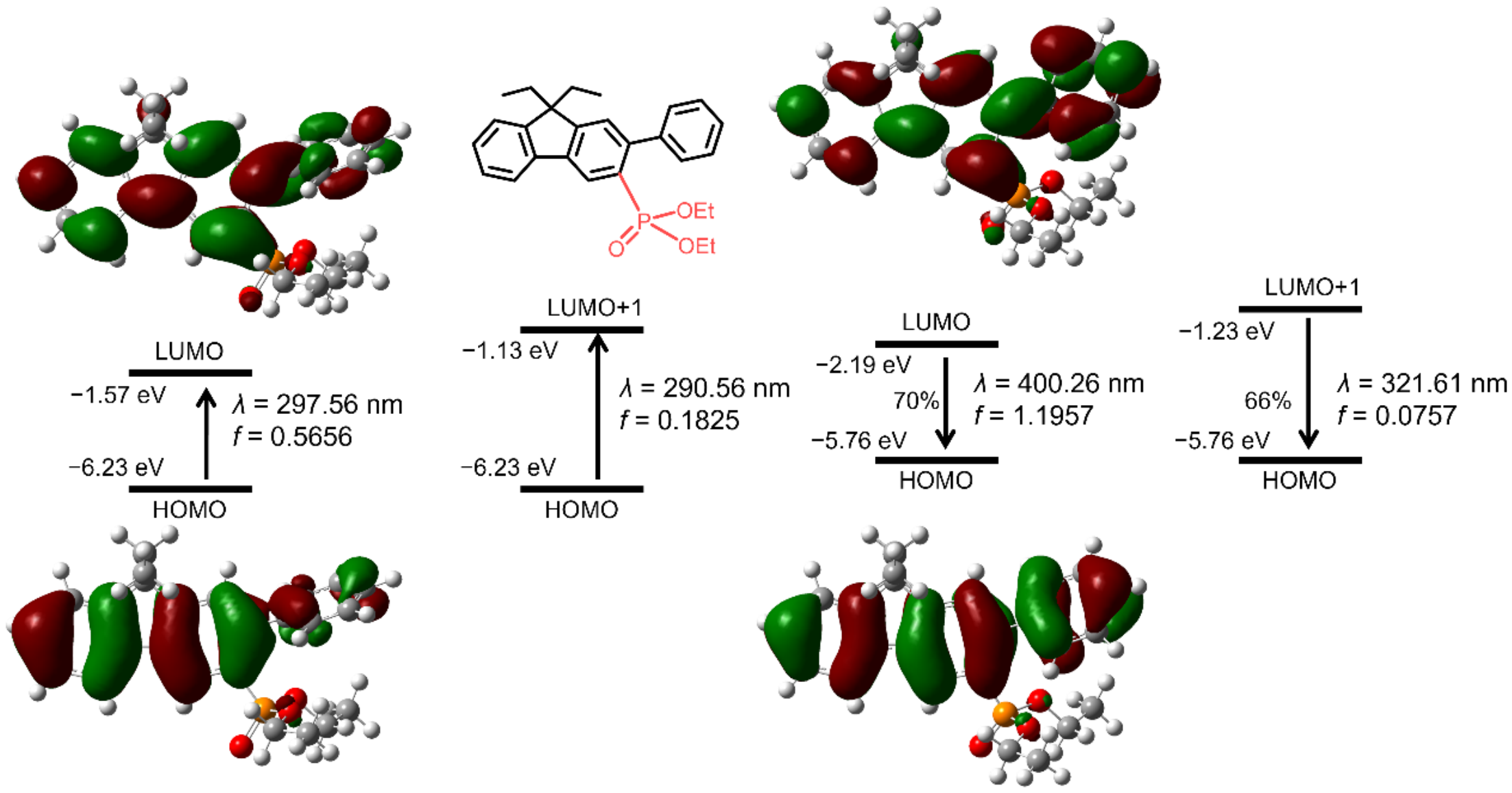

Figure S13. Orbital correlation and composition of the $S_0 \rightarrow S_1$ (left side) and the $S_1 \rightarrow S_0$ (right side) transition for 3P-PF calculated at B3LYP/6-311G+(d, p) level basis set. Molecular orbitals of HOMO and LUMO are described (iso value = 0.02). λ and f mean wavelength and oscillator strength, respectively.

12. DFT simulation about stable structure of EDA complex

Table S3. Summary for the optimization of the EDA complex position.

	A 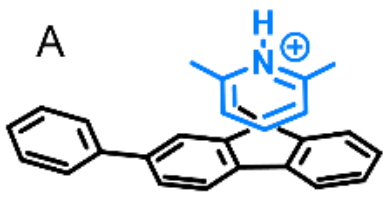	B 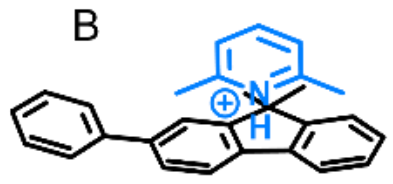	C 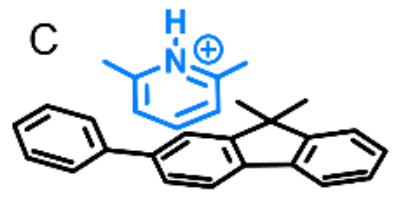	D 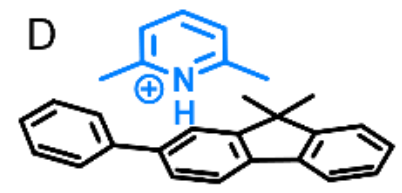
Relative Energy (S_0) [kcal mol^{-1}]	0.00	0.29	−0.16	−0.75
Oscillator Strength	0.06	0.01	0.01	0.05
Excitation Energy [kcal mol^{-1}]	90.87	89.78	92.95	92.79
Optimized chemical structure	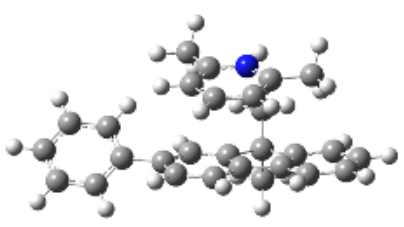	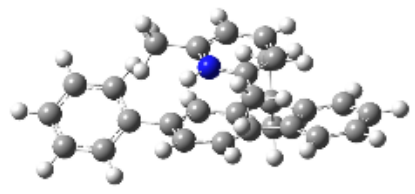	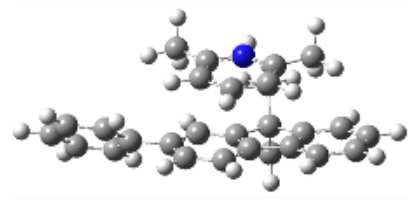	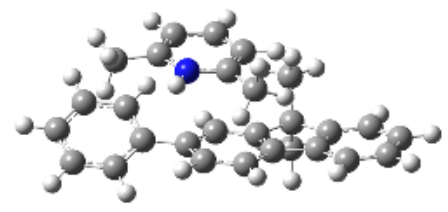

13. DFT calculation

Table S4. Cartesian coordinates of the optimized structure for PFO model (**S$_0$**).

Symbol	X	Y	Z	Symbol	X	Y	Z
C	-4.64501	1.76239	-0.334643	H	-0.715945	-1.211597	-2.490358
C	-5.06218	0.455653	-0.07361	H	-1.581178	-2.684626	-2.953834
C	-4.125926	-0.5617	0.125099	H	-2.484463	-1.238551	-2.47878
C	-2.774667	-0.255748	0.059313	C	-1.561553	-1.813709	1.65329
C	-2.357399	1.056924	-0.202992	H	-0.673666	-2.45484	1.725341
C	-3.287288	2.073432	-0.401235	H	-2.430483	-2.478392	1.741442
H	-5.384655	2.542305	-0.486851	C	-1.563888	-0.809957	2.804177
H	-6.122934	0.229713	-0.024891	H	-1.550643	-1.330896	3.765937
H	-4.456595	-1.577093	0.328216	H	-0.686444	-0.157488	2.761956
H	-2.966484	3.091089	-0.604439	H	-2.455614	-0.176361	2.775247
C	-1.583493	-1.184558	0.240354	C	1.378643	1.845267	-0.367641
C	-0.425692	-0.2189	0.040053	H	2.090863	2.644515	-0.550355
C	0.932055	-0.485925	0.089598	C	5.216926	-1.12248	-0.627771
C	1.855055	0.550338	-0.115561	H	3.191118	-1.542763	-1.208594
C	0.014034	2.118228	-0.417112	C	6.074897	-0.241644	0.027447
C	-0.887968	1.079134	-0.212477	H	6.213429	1.587181	1.155745
H	1.289532	-1.489159	0.309242	H	5.613555	-2.009633	-1.111992
H	-0.331998	3.127243	-0.621712	H	7.141023	-0.442342	0.063718
C	3.314528	0.27836	-0.066768	C	-1.573009	-2.311784	-0.818605
C	4.186998	1.155138	0.589585	H	-0.683794	-2.931428	-0.64497
C	3.850046	-0.863775	-0.674863	H	-2.440383	-2.958483	-0.634106
C	5.554386	0.898759	0.635616	C	-1.589589	-1.832129	-2.268413
H	3.786334	2.033729	1.087				

Table S5. Cartesian coordinates of the optimized structure for PFO model (S$_1$)

Symbol	X	Y	Z	Symbol	X	Y	Z
C	-4.61155	1.834274	-0.09308	H	-0.726274	-0.873982	-2.628327
C	-5.047586	0.498905	-0.01272	H	-1.60891	-2.253512	-3.299553
C	-4.121385	-0.55364	0.044273	H	-2.49677	-0.881883	-2.617813
C	-2.77257	-0.26425	0.019905	C	-1.581652	-2.035495	1.388316
C	-2.321162	1.08994	-0.062516	H	-0.699376	-2.688514	1.374746
C	-3.264306	2.147594	-0.118588	H	-2.458598	-2.695683	1.387395
H	-5.34932	2.629838	-0.135556	C	-1.574741	-1.187754	2.658142

H	-6.111287	0.283495	0.005618	H	-1.567497	-1.826356	3.546044
H	-4.467237	-1.582169	0.107217	H	-0.689946	-0.544596	2.697983
H	-2.934475	3.18004	-0.180298	H	-2.460447	-0.546458	2.709948
C	-1.593673	-1.223155	0.072305	C	1.36515	1.893505	-0.112224
C	-0.424019	-0.2501	0.004525	H	2.064248	2.717858	-0.184763
C	0.908878	-0.527764	0.015398	C	5.163799	-1.296392	-0.159988
C	1.878411	0.541406	-0.03732	H	3.12855	-1.876919	-0.334801
C	0.028078	2.176562	-0.134305	C	6.083963	-0.256192	0.011034
C	-0.908545	1.102548	-0.070546	H	6.309841	1.868496	0.308573
H	1.236931	-1.557782	0.099379	H	5.51775	-2.314861	-0.289678
H	-0.31507	3.203662	-0.207738	H	7.149775	-0.459789	0.023657
C	3.286209	0.279556	-0.021873	C	-1.595954	-2.192697	-1.132439
C	4.252923	1.31988	0.148232	H	-0.711417	-2.83747	-1.049091
C	3.80432	-1.045393	-0.173591	H	-2.470513	-2.849314	-1.03821
C	5.60831	1.051934	0.166081	C	-1.607882	-1.509514	-2.497825
H	3.929954	2.343357	0.298072				

Table S6. Cartesian coordinates of the optimized structure for 3P-PFO model (S_0)

Symbol	X	Y	Z	Symbol	X	Y	Z
C	-5.183372	-2.352707	-0.760422	H	-3.843765	-0.209729	2.688509
C	-5.996854	-1.273872	-0.407375	C	-3.502112	2.486747	-0.608757
C	-5.433833	-0.050613	-0.036749	H	-2.857689	3.322285	-0.306874
C	-4.052644	0.077357	-0.024049	H	-4.534873	2.815124	-0.436065
C	-3.238577	-1.007475	-0.378123	C	-3.298569	2.190293	-2.093022
C	-3.794687	-2.228058	-0.748535	H	-3.522321	3.076421	-2.693909
H	-5.636831	-3.296289	-1.047343	H	-2.265751	1.894151	-2.301079
H	-7.076375	-1.387837	-0.422182	H	-3.953215	1.380458	-2.428801
H	-6.072743	0.7854	0.235989	C	0.574051	-0.605616	-0.327183
H	-3.163679	-3.06758	-1.025047	P	2.02898	-1.618449	-0.675923
C	-3.217316	1.296773	0.337643	O	1.695935	-2.89747	-1.349459
C	-1.8111	0.755544	0.141282	O	3.001331	-0.601796	-1.420851
C	-0.603794	1.410634	0.315804	O	2.812738	-1.832461	0.726107
C	0.60786	0.74225	0.094092	C	2.350997	-2.858764	1.618713
C	-0.650775	-1.260004	-0.512244	H	1.319012	-2.633618	1.918047
C	-1.836448	-0.580284	-0.271023	H	2.356077	-3.817428	1.091697
H	-0.577614	2.448693	0.637805	C	4.409669	-0.852799	-1.575975

H	-0.64707	-2.287807	-0.862842	H	4.540982	-1.699248	-2.257902
C	1.86267	1.526346	0.294397	H	4.82938	-1.112201	-0.599331
C	2.771051	1.214725	1.309125	C	5.038633	0.410472	-2.121359
C	3.250637	3.416047	-0.325296	H	6.114578	0.260042	-2.253015
C	3.911951	1.989756	1.499061	H	4.602169	0.673287	-3.088547
H	2.588178	0.354559	1.942856	H	4.880373	1.241207	-1.428418
C	4.156424	3.092429	0.682941	C	3.269404	-2.876104	2.82208
H	3.433177	4.271208	-0.968792	H	2.9471	-3.650687	3.524212
H	4.609158	1.733217	2.290881	H	4.297428	-3.089544	2.517446
H	5.046078	3.696357	0.832597	H	3.252971	-1.912457	3.339057
C	-3.454107	1.739441	1.800747	C	2.111477	2.638909	-0.515485
H	-2.813393	2.60756	2.00124	H	1.413388	2.882795	-1.311108
H	-4.489489	2.093731	1.882333	H	-2.158591	0.314575	2.816258
C	-3.197125	0.658376	2.848397	H	-3.392433	1.04509	3.852702

Table S7. Cartesian coordinates of the optimized structure for 4P-PFO model (S_0)

Symbol	X	Y	Z	Symbol	X	Y	Z
C	-4.521226	-0.273761	-0.656149	C	-1.079369	-3.596762	1.189379
C	-4.747219	-1.647781	-0.564391	H	-0.112983	-4.113138	1.255467
C	-3.676544	-2.523737	-0.387025	H	-1.842544	-4.381253	1.109411
C	-2.392908	-2.007562	-0.286771	C	-1.314551	-2.779255	2.457826
C	-2.164499	-0.624329	-0.350792	H	-1.29108	-3.426056	3.339662
C	-3.233513	0.24928	-0.555789	H	-0.54689	-2.009483	2.583188
H	-5.357205	0.399232	-0.820203	H	-2.287401	-2.279217	2.431001
H	-5.757738	-2.036468	-0.645428	C	1.459641	0.628552	0.03903
H	-3.847053	-3.595916	-0.33414	C	4.134793	-1.651898	1.118808
H	-3.07309	1.313284	-0.678779	H	3.495049	-2.295067	1.716396
C	-1.098953	-2.779417	-0.124173	H	2.063535	1.522225	0.144913
C	-0.089501	-1.648268	-0.083167	P	-0.627634	2.420732	-0.153695
C	1.282293	-1.767801	0.055288	O	0.604882	3.423222	0.123017
C	2.081904	-0.621714	0.108726	O	-1.43639	2.422339	1.22912
C	0.077674	0.764045	-0.115057	O	-1.420037	2.794352	-1.35453
C	-0.711276	-0.395094	-0.193981	C	-2.459556	3.40157	1.484003
H	1.74242	-2.751702	0.106787	H	-1.983978	4.285009	1.923093
C	3.556145	-0.718905	0.250221	H	-2.927595	3.687448	0.536241
C	4.39975	0.121597	-0.486462	C	1.072037	4.319717	-0.904244

C	5.517798	-1.742827	1.247056	H	1.431358	5.200381	-0.367126
C	5.782669	0.032876	-0.357455	H	0.228524	4.605957	-1.537951
H	3.96729	0.834747	-1.182716	C	2.180599	3.690038	-1.726753
C	6.347107	-0.900351	0.509684	H	2.538957	4.408627	-2.470381
H	5.948067	-2.467223	1.93172	H	3.024921	3.403211	-1.092675
H	6.420788	0.687951	-0.942574	H	1.818344	2.804256	-2.257412
H	7.425579	-0.97053	0.610137	C	-3.467049	2.783501	2.430172
C	-0.848723	-3.727695	-1.320483	H	-4.256521	3.506682	2.65661
H	0.106622	-4.24174	-1.153496	H	-3.920499	1.897717	1.976908
H	-1.623379	-4.504745	-1.30312	H	-2.986525	2.487092	3.36618
C	-0.834308	-3.046908	-2.687373	H	-0.654177	-3.781004	-3.477968
H	-0.046572	-2.289524	-2.74528	H	-1.788649	-2.553699	-2.89465

Table S8. Cartesian coordinates of the optimized structure for Complex A.

Symbol	X	Y	Z	Symbol	X	Y	Z
C	-4.800309	-2.097074	0.948403	H	-0.536121	-1.977637	-3.53318
C	-5.150696	-2.227902	-0.397802	H	-2.246254	-2.414654	-3.660232
C	-4.224427	-1.944558	-1.406125	C	0.954475	-0.363295	1.244437
C	-2.946356	-1.535193	-1.049313	H	1.604112	-0.175211	2.093832
C	-2.595491	-1.410185	0.304095	C	0.837666	-4.106382	0.017657
C	-3.517332	-1.686682	1.310856	C	1.276545	-3.739129	1.275669
H	-5.532001	-2.321092	1.718133	C	-0.92162	-4.207952	2.168207
H	-6.152166	-2.551726	-0.662955	C	-1.350551	-4.578116	0.90455
H	-4.508102	-2.043048	-2.450418	H	-1.228382	-3.200965	-2.451141
H	-3.245662	-1.590761	2.358034	H	-2.360282	0.958814	-2.016301
C	-1.783837	-1.148287	-1.953362	C	0.393483	-3.794038	2.350842
C	-0.68678	-0.895575	-0.927301	H	0.732759	-3.501405	3.338243
C	0.638802	-0.55648	-1.146455	H	2.296989	-3.401872	1.403671
C	1.482825	-0.299701	-0.053544	H	-1.619601	-4.242975	2.994688
C	-0.375816	-0.706362	1.469616	N	-0.448458	-4.516245	-0.101613
C	-1.189787	-0.991669	0.378516	H	-0.76002	-4.812743	-1.021645
H	1.023796	-0.46394	-2.159155	C	-2.730248	-5.036447	0.566635
H	-0.75745	-0.76962	2.48443	H	-3.349668	-5.05463	1.462263
C	2.915412	0.031589	-0.268918	H	-3.189445	-4.352921	-0.155412
C	3.555271	1.005225	0.509732	H	-2.708494	-6.039844	0.131284
C	3.660466	-0.627868	-1.255575	C	1.673147	-4.083541	-1.219921

C	4.900059	1.307068	0.310029	H	1.276859	-3.349359	-1.928277
H	2.991771	1.546571	1.263951	H	2.69857	-3.809486	-0.974327
C	5.004946	-0.32592	-1.45745	H	1.677904	-5.063242	-1.706142
H	3.189185	-1.39509	-1.862661	C	-2.104787	0.155154	-2.7127
C	5.630809	0.642302	-0.673893	H	-1.241086	0.471726	-3.305682
H	5.375183	2.070012	0.918775	H	-2.949891	0.001577	-3.39086
H	5.564996	-0.852447	-2.223962	C	-1.424774	-2.252122	-2.956297
H	6.678521	0.878555	-0.830045				

Table S9. Cartesian coordinates of the optimized structure for Complex B.

Symbol	X	Y	Z	Symbol	X	Y	Z
C	-4.902549	-2.194657	0.550044	H	-0.325745	-1.9089	-3.588818
C	-5.15305	-2.292223	-0.821692	H	-2.006995	-2.412168	-3.822303
C	-4.164483	-1.960563	-1.752325	C	0.794679	-0.427186	1.308652
C	-2.924001	-1.536901	-1.293747	H	1.384554	-0.276119	2.20824
C	-2.675285	-1.44251	0.083402	C	0.361627	-4.171381	-0.133372
C	-3.658825	-1.766962	1.014032	C	1.160694	-3.834858	0.946812
H	-5.682544	-2.454966	1.258712	C	-0.720966	-4.080808	2.423065
H	-6.125944	-2.627771	-1.167019	C	-1.523624	-4.435305	1.353982
H	-4.369991	-2.036155	-2.816658	H	-1.033963	-3.150328	-2.539774
H	-3.461641	-1.697536	2.080281	H	-2.320278	0.976271	-2.210648
C	-1.702972	-1.117099	-2.101393	C	-0.978698	-4.478524	0.074362
C	-0.693496	-0.859484	-0.991004	H	-1.610266	-4.746162	-0.765792
C	0.639609	-0.500481	-1.104345	H	-2.569419	-4.655602	1.52388
C	1.405979	-0.287927	0.052834	H	0.796631	-4.192605	-1.124497
C	-0.546276	-0.784133	1.427073	N	0.581129	-3.810358	2.168458
C	-1.284915	-1.012825	0.2711	H	1.167195	-3.549196	2.956388
H	1.091546	-0.364714	-2.083467	C	-1.192137	-3.957204	3.835128
H	-0.99654	-0.892463	2.409992	H	-1.090284	-2.924202	4.18147
C	2.845404	0.067287	-0.04853	H	-2.24041	-4.244196	3.907299
C	3.421587	0.996574	0.827888	H	-0.606971	-4.599269	4.499144
C	3.662086	-0.52769	-1.019649	C	2.618854	-3.524446	0.861866
C	4.773802	1.317907	0.738574	H	2.923037	-3.43484	-0.180302
H	2.803042	1.490122	1.571726	H	2.8506	-2.585143	1.370629
C	5.013778	-0.20606	-1.111194	H	3.201895	-4.324696	1.327924
H	3.241422	-1.262679	-1.699908	C	-1.988377	0.179987	-2.883102

C	5.575761	0.717372	-0.230843	H	-1.086153	0.51819	-3.40234
H	5.198855	2.046387	1.421958	H	-2.769725	0.0089	-3.630193
H	5.629709	-0.682856	-1.867327	C	-1.238362	-2.216179	-3.06854
H	6.629213	0.968659	-0.301218				

Table S10. Cartesian coordinates of the optimized structure for Complex C.

Symbol	X	Y	Z	Symbol	X	Y	Z
C	-4.130476	-2.276115	1.036518	H	-0.119302	-2.056724	-3.642838
C	-4.542943	-2.496343	-0.280199	H	-1.780143	-2.672057	-3.63861
C	-3.70647	-2.173495	-1.35262	C	1.457301	-0.045912	0.917494
C	-2.453472	-1.635916	-1.089473	H	2.124115	0.264506	1.715982
C	-2.041161	-1.417935	0.234268	C	1.873813	-3.577294	0.63638
C	-2.874029	-1.733056	1.304913	C	2.059191	-3.326795	1.984875
H	-4.793421	-2.532016	1.856985	C	-0.207523	-4.044399	2.420222
H	-5.523089	-2.921462	-0.47166	C	-0.377077	-4.310501	1.072096
H	-4.036702	-2.346594	-2.373196	H	-0.627488	-3.240146	-2.42505
H	-2.552836	-1.567198	2.329245	H	-2.15188	0.817946	-2.286772
C	-1.374225	-1.212343	-2.076639	C	1.014649	-3.557896	2.874644
C	-0.261663	-0.78621	-1.128701	H	1.153195	-3.354356	3.930599
C	1.010839	-0.337603	-1.441685	H	3.011607	-2.943025	2.326687
C	1.893343	0.033452	-0.413402	H	-1.028407	-4.227149	3.101769
C	0.179754	-0.500076	1.237345	N	0.668962	-4.058743	0.25118
C	-0.675079	-0.879858	0.208466	H	0.542688	-4.254232	-0.73746
H	1.338001	-0.293341	-2.477456	C	-1.616059	-4.87618	0.461192
H	-0.13463	-0.554032	2.275399	H	-2.404291	-4.946767	1.209201
C	3.272148	0.486743	-0.733154	H	-1.973918	-4.239169	-0.353259
C	4.355784	0.111366	0.072355	H	-1.418018	-5.875181	0.061184
C	3.522489	1.291788	-1.852501	C	2.901986	-3.367323	-0.425518
C	5.650232	0.524246	-0.232742	H	2.515609	-2.700665	-1.201885
H	4.188946	-0.521206	0.939365	H	3.799002	-2.918704	-0.000697
C	4.816643	1.70432	-2.159704	H	3.172965	-4.320836	-0.888636
H	2.69596	1.617107	-2.477346	C	-1.847001	-0.016186	-2.925073
C	5.88632	1.3212	-1.351818	H	-1.041431	0.326371	-3.582138
H	6.47598	0.217023	0.401479	H	-2.698474	-0.304996	-3.549027
H	4.987887	2.333636	-3.027457	C	-0.947917	-2.365073	-2.998136
H	6.894897	1.642861	-1.590984				

Table S11. Cartesian coordinates of the optimized structure for Complex D and INI (S_0).

Symbol	X	Y	Z	Symbol	X	Y	Z
C	-5.762733	-1.794354	-0.138996	H	-0.563292	-2.236326	-3.390333
C	-5.803762	-1.903166	-1.531656	H	-2.221944	-2.556448	-3.924893
C	-4.649547	-1.710515	-2.295781	C	-0.104279	-0.538414	1.485157
C	-3.457993	-1.409321	-1.649848	H	0.344198	-0.416775	2.466501
C	-3.420026	-1.297789	-0.251552	C	0.683538	-4.154573	-0.497946
C	-4.567999	-1.489645	0.513778	C	1.943569	-3.953738	0.055526
H	-6.668797	-1.94704	0.438936	C	-0.322264	-3.890873	1.661523
H	-6.741836	-2.139498	-2.024167	H	-1.623591	-3.290957	-2.429531
H	-4.691257	-1.796795	-3.378145	H	-2.431999	0.962458	-2.612477
H	-4.539408	-1.403937	1.596253	C	-0.453207	-4.119173	0.303315
C	-2.083923	-1.163503	-2.257868	H	-1.44155	-4.266755	-0.113522
C	-1.248365	-0.926345	-1.007586	H	0.587822	-4.335832	-1.562843
C	0.110711	-0.674643	-0.917582	N	0.925706	-3.68917	2.146121
C	0.707794	-0.49033	0.340529	C	2.060809	-3.69415	1.409944
C	-1.474018	-0.772364	1.398161	H	2.836532	-3.975479	-0.555481
C	-2.041681	-0.984333	0.146343	H	1.017651	-3.509107	3.14159
H	0.712028	-0.609494	-1.820435	C	3.344087	-3.369957	2.100033
H	-2.077628	-0.807067	2.300222	H	3.393227	-3.82653	3.091226
C	2.173903	-0.274058	0.466336	H	4.18865	-3.717915	1.505956
C	2.706526	0.489777	1.514818	H	3.431451	-2.282987	2.210897
C	3.067049	-0.854073	-0.445631	C	-1.460406	-3.887764	2.628641
C	4.082729	0.647495	1.659607	H	-1.328563	-3.125209	3.399571
H	2.041662	0.983554	2.216548	H	-2.396008	-3.694833	2.1029
C	4.442695	-0.695728	-0.304451	H	-1.533224	-4.864064	3.118108
H	2.687143	-1.457664	-1.263496	C	-2.087407	0.081948	-3.162352
C	4.958187	0.050478	0.753876	H	-1.079889	0.282342	-3.540547
H	4.470422	1.246028	2.477884	H	-2.749165	-0.071766	-4.020422
H	5.112651	-1.164759	-1.018232	C	-1.591423	-2.388241	-3.046416
H	6.030601	0.170761	0.868177				

Table S12. Cartesian coordinates of the optimized structure for INI (S_1).

Symbol	X	Y	Z	Symbol	X	Y	Z
C	-5.40202	-1.975687	0.243611	H	-0.438529	-2.188441	-3.468612

C	-5.509387	-2.175823	-1.142148	H	-2.120449	-2.491465	-3.925831
C	-4.419128	-1.974377	-1.994527	C	0.204662	-0.413128	1.492515
C	-3.2102	-1.585175	-1.445057	H	0.67859	-0.174164	2.436061
C	-3.093656	-1.402315	-0.045225	C	0.559853	-4.079742	-0.633729
C	-4.198762	-1.582444	0.80586	C	1.648586	-3.674768	0.159449
H	-6.270501	-2.130428	0.874391	C	-0.865807	-3.97991	1.320534
H	-6.462267	-2.48401	-1.559962	H	-1.408543	-3.357711	-2.554853
H	-4.52854	-2.119747	-3.064571	H	-2.357485	0.869087	-2.317593
H	-4.111841	-1.422908	1.875574	C	-0.698271	-4.244797	-0.013339
C	-1.902186	-1.268122	-2.151326	H	-1.560678	-4.580179	-0.578813
C	-0.987385	-1.030788	-0.959179	H	0.695909	-4.304148	-1.683409
C	0.352414	-0.781339	-0.938946	N	0.23039	-3.555099	2.04954
C	1.005941	-0.497468	0.301406	C	1.489773	-3.398301	1.494709
C	-1.133762	-0.688891	1.484849	H	2.634726	-3.555148	-0.274642
C	-1.743226	-1.047219	0.256878	H	0.114256	-3.380558	3.037114
H	0.91421	-0.762855	-1.865021	C	2.581132	-2.982181	2.423048
H	-1.711504	-0.649509	2.401618	H	2.758842	-3.743038	3.19139
C	2.438211	-0.254314	0.353168	H	3.509072	-2.822469	1.871915
C	3.035209	0.419537	1.443747	H	2.328458	-2.047411	2.939502
C	3.285996	-0.707827	-0.684673	C	-2.141289	-4.143642	2.07696
C	4.407116	0.616468	1.495761	H	-2.411041	-3.215345	2.595536
H	2.424336	0.822161	2.243052	H	-2.956136	-4.406116	1.401297
C	4.656411	-0.519382	-0.621049	H	-2.051802	-4.927928	2.836649
H	2.873789	-1.251644	-1.526673	C	-2.040742	0.041457	-2.958359
C	5.225901	0.142498	0.470146	H	-1.083117	0.303201	-3.418169
H	4.840795	1.144985	2.337868	H	-2.781418	-0.08645	-3.753074
H	5.28721	-0.894105	-1.419968	C	-1.437502	-2.400497	-3.076182
H	6.299546	0.291013	0.517072				

Table S13. Cartesian coordinates of the optimized structure for IM1 (S_1) of the 4-position phosphonylation.

Symbol	X	Y	Z	Symbol	X	Y	Z
C	2.870266	-3.570133	-1.202697	H	-1.755387	-4.08687	0.120411
C	3.078307	-3.980918	0.123545	H	-4.741972	-1.021549	0.779475
C	2.352614	-3.424465	1.180707	C	-3.250328	-2.592816	0.655542
C	1.406175	-2.453697	0.900254	H	-3.345121	-2.901972	1.687993

C	1.191418	-2.04257	-0.43663	N	-3.050126	-1.840356	-1.987451
C	1.931477	-2.594026	-1.496345	H	-2.989932	-1.576733	-2.959944
H	3.454878	-4.017716	-1.99891	C	-4.748329	-0.08487	-1.816403
H	3.823592	-4.741743	0.331342	H	-5.412823	-0.499435	-2.583089
H	2.537665	-3.749742	2.199622	H	-5.356438	0.432956	-1.073058
H	1.774042	-2.264816	-2.518228	H	-4.106097	0.658271	-2.306415
C	0.509737	-1.691663	1.863408	C	-1.370845	-3.525738	-2.546351
C	-0.302335	-0.853487	0.889279	H	-1.954063	-3.998318	-3.344493
C	-1.306579	0.028309	1.149205	H	-0.715315	-2.782446	-3.017124
C	-1.858942	0.808784	0.08763	H	-0.744219	-4.28501	-2.076823
C	-0.328246	-0.245809	-1.506831	P	2.714749	1.428853	-0.69383
C	0.166559	-1.045491	-0.448512	O	1.476492	2.068927	0.15255
H	-1.650538	0.174816	2.166561	O	3.506823	0.687532	0.542868
H	0.061478	-0.351763	-2.51291	O	3.764764	2.700566	-0.841519
C	-2.904736	1.785486	0.349068	C	1.664109	2.874907	1.331864
C	-3.139598	2.864698	-0.531575	H	2.249543	2.30912	2.062743
C	-3.724668	1.677739	1.494804	H	2.227658	3.772755	1.058674
C	-4.150505	3.78148	-0.281608	C	0.299384	3.229233	1.882416
H	-2.508484	3.011986	-1.400201	H	-0.234209	2.330145	2.201229
C	-4.741279	2.588239	1.733003	H	0.410481	3.890652	2.746666
H	-3.593348	0.850924	2.183261	H	-0.304849	3.741433	1.127915
C	-4.960861	3.645039	0.845781	C	4.511789	-0.287708	0.222864
H	-4.305178	4.608393	-0.966418	H	5.32613	0.196508	-0.328249
H	-5.370282	2.47372	2.609324	H	4.073467	-1.065515	-0.41416
H	-5.755541	4.358961	1.035101	C	5.025457	-0.88285	1.51645
C	1.34464	-0.762553	2.768585	H	5.785571	-1.638391	1.297057
H	0.683275	-0.161688	3.400701	H	5.474092	-0.110483	2.14713
H	1.990334	-1.358843	3.420994	H	4.213318	-1.362154	2.068567
C	-0.346417	-2.630032	2.725624	C	3.514137	3.648137	-1.889429
H	-1.071007	-2.054877	3.309605	H	2.702628	4.316879	-1.577876
H	0.291892	-3.179574	3.423798	H	3.190836	3.122661	-2.797746
C	-1.305759	0.665791	-1.230143	C	4.784425	4.430995	-2.14818
H	-1.700158	1.2668	-2.039492	H	4.609403	5.174717	-2.931075
C	-4.042614	-1.550028	0.141665	H	5.106627	4.952005	-1.242401
C	-3.937231	-1.157201	-1.169622	H	5.588587	3.765672	-2.474123
C	-2.260564	-2.883087	-1.535676	H	-0.890154	-3.352303	2.115818

C	-2.370085	-3.262759	-0.224165	H	1.971195	-0.095564	2.171995

Table S14. Cartesian coordinates of the optimized structure for IM2 (S_0) of the 4-position phosphonylation.

Symbol	X	Y	Z	Symbol	X	Y	Z
C	4.415359	-1.752565	-0.291097	H	1.621612	-3.712712	-1.369902
C	4.538903	-2.475342	0.898784	H	-2.575343	-3.693729	-0.295693
C	3.488453	-2.512938	1.822906	C	-0.420333	-3.781551	-0.628035
C	2.317557	-1.830226	1.537657	H	-0.233154	-4.547585	0.110909
C	2.191494	-1.103437	0.336605	N	-0.930838	-1.928732	-2.62404
C	3.242141	-1.0601	-0.5813	H	-1.152533	-1.4456	-3.482336
H	5.238416	-1.736499	-0.998654	C	-3.350777	-1.924831	-2.265303
H	5.458283	-3.013064	1.107174	H	-3.583758	-2.173142	-3.307692
H	3.59485	-3.076391	2.745656	H	-4.120997	-2.359729	-1.626833
H	3.146094	-0.517647	-1.516211	H	-3.405706	-0.833915	-2.163011
C	1.05703	-1.693252	2.375734	C	1.368455	-1.96358	-3.485809
C	0.139537	-0.965911	1.403366	H	1.095382	-2.271561	-4.502555
C	-1.21037	-0.69573	1.549006	H	1.450804	-0.867795	-3.498364
C	-1.91193	-0.009902	0.503594	H	2.354202	-2.372638	-3.258227
C	0.258281	0.40126	-0.686061	P	0.938638	2.061558	-0.342692
C	0.854986	-0.559538	0.284337	O	0.622884	2.327522	1.161738
H	-1.746007	-1.004391	2.439653	O	2.491489	2.146363	-0.530989
H	0.552806	0.19012	-1.723128	O	0.341376	3.17427	-1.280733
C	-3.392731	0.09416	0.574432	C	1.140883	3.463091	1.928288
C	-4.059835	1.244654	0.138167	H	2.200904	3.273695	2.104334
C	-4.15227	-0.979136	1.056135	H	1.026182	4.369848	1.329159
C	-5.450437	1.316622	0.168916	C	0.346717	3.528991	3.208958
H	-3.487631	2.097452	-0.214539	H	0.450703	2.600651	3.77552
C	-5.542024	-0.908874	1.088301	H	0.724384	4.353586	3.81901
H	-3.652882	-1.888241	1.377778	H	-0.711474	3.704735	3.00127
C	-6.196608	0.238878	0.642649	C	3.177714	2.455898	-1.788029
H	-5.950393	2.218582	-0.169936	H	2.884316	3.46483	-2.081279
H	-6.114779	-1.755162	1.45434	H	2.843677	1.746849	-2.550032
H	-7.280232	0.294118	0.667598	C	4.658895	2.341268	-1.527297
C	1.333056	-0.760987	3.577863	H	5.197511	2.570266	-2.450616
H	0.410547	-0.583297	4.13977	H	4.97269	3.049498	-0.757119

H	2.064797	-1.219635	4.249917	H	4.921816	1.32924	-1.21057
C	0.503148	-3.031158	2.873486	C	-0.983447	3.784082	-1.136442
H	-0.428448	-2.877106	3.427378	H	-1.352552	3.585072	-0.126972
H	1.217853	-3.510568	3.549667	H	-1.630074	3.291435	-1.863804
C	-1.240211	0.485341	-0.574531	C	-0.849349	5.262313	-1.408596
H	-1.773191	0.933301	-1.404995	H	-1.83756	5.724575	-1.337768
C	-1.742063	-3.32599	-0.884634	H	-0.190959	5.739263	-0.678708
C	-2.000481	-2.433371	-1.880104	H	-0.456099	5.437351	-2.412434
C	0.36491	-2.440444	-2.48592	H	0.302717	-3.704388	2.039064
C	0.60839	-3.344492	-1.490978	H	1.725265	0.203615	3.241426

Table S15. Cartesian coordinates of the optimized structure for IM3 (S_0) of the 4-position phosphonylation.

Symbol	X	Y	Z	Symbol	X	Y	Z
C	-4.291778	-1.968911	-0.212667	H	0.903523	-2.734629	-3.429079
C	-4.311399	-2.569429	-1.474966	H	-0.683872	-3.480043	-3.674113
C	-3.198491	-2.49292	-2.319929	C	1.212615	0.51472	0.62677
C	-2.075137	-1.806108	-1.889583	H	1.680432	0.889604	1.530592
C	-2.058971	-1.190711	-0.621732	P	-1.056871	1.97552	0.422825
C	-3.166208	-1.275558	0.226248	O	-0.701746	2.386176	-1.039086
H	-5.16093	-2.04628	0.432877	O	-2.615627	1.938487	0.54914
H	-5.19733	-3.105476	-1.799966	O	-0.562577	3.031368	1.475002
H	-3.220531	-2.970159	-3.295595	C	-1.291694	3.532792	-1.737806
H	-3.153764	-0.82368	1.212917	H	-2.321552	3.264071	-1.977515
C	-0.754064	-1.586957	-2.608489	H	-1.285558	4.391705	-1.062414
C	0.028049	-0.809038	-1.56036	C	-0.457292	3.776937	-2.969871
C	1.353924	-0.389752	-1.626266	H	-0.455324	2.895759	-3.615558
C	1.962055	0.218324	-0.475754	H	-0.884436	4.615308	-3.526141
C	-0.283293	0.327358	0.649441	H	0.571644	4.025623	-2.699952
C	-0.765516	-0.5632	-0.450189	C	-3.381922	2.109608	1.788322
H	1.950189	-0.566527	-2.515496	H	-3.142933	3.096048	2.188133
H	-0.624266	-0.002051	1.641082	H	-3.062094	1.343132	2.498556
C	3.427536	0.471399	-0.478264	C	-4.840123	1.972154	1.428637
C	3.954222	1.64879	0.066163	H	-5.438986	2.090619	2.335392
C	4.309879	-0.469657	-1.024049	H	-5.137018	2.74195	0.712838
C	5.327684	1.878192	0.072056	H	-5.045153	0.988119	1.000575

H	3.284769	2.40053	0.471875	C	0.70612	3.766136	1.418578
C	5.683453	-0.24176	-1.017467	H	1.11768	3.675339	0.410012
H	3.922428	-1.395878	-1.438159	H	1.372217	3.279422	2.13201
C	6.197062	0.93297	-0.469574	C	0.434394	5.203342	1.789235
H	5.717359	2.799758	0.492732	H	1.382071	5.748576	1.795641
H	6.353542	-0.985601	-1.436599	H	-0.232621	5.678994	1.066381
H	7.26754	1.111429	-0.466977	H	-0.009634	5.269658	2.784885
C	-0.950811	-0.723688	-3.8694	H	0.079333	-3.530359	-2.072147
H	0.013258	-0.508744	-4.34059	H	-1.434815	0.225285	-3.619651
C	-0.070699	-2.916225	-2.964733	H	-1.576182	-1.251938	-4.595482

Table S16. Cartesian coordinates of the optimized structure for IM4 (S_0) of the 4-position phosphonylation.

Symbol	X	Y	Z	Symbol	X	Y	Z
C	-5.082767	-1.375511	-0.660227	C	1.864654	-2.903917	2.198254
C	-5.086162	-2.038391	-1.890369	C	-0.533226	-2.887189	2.044372
C	-3.901868	-2.188867	-2.621935	C	-0.469033	-3.585884	0.852593
C	-2.724259	-1.671691	-2.108508	P	-1.170041	2.037328	0.183993
C	-2.719904	-0.995481	-0.87125	O	-0.720248	2.519942	-1.145473
C	-3.901925	-0.84771	-0.141088	O	-2.750587	2.260236	0.364722
H	-6.009082	-1.267631	-0.104676	O	-0.471437	2.725421	1.455559
H	-6.01529	-2.440538	-2.281451	C	-3.401673	2.265843	1.652217
H	-3.913234	-2.70666	-3.577073	H	-2.970365	3.064018	2.261461
H	-3.909432	-0.333169	0.813924	H	-3.216654	1.31109	2.159902
C	-1.324991	-1.707927	-2.700096	C	-4.880298	2.484047	1.42329
C	-0.519357	-1.045625	-1.590513	H	-5.401275	2.485972	2.384714
C	0.863213	-0.903359	-1.514455	H	-5.053412	3.444481	0.931638
C	1.451485	-0.374437	-0.314729	H	-5.300148	1.691074	0.798881
C	-0.833681	0.244226	0.533172	C	0.09983	4.055213	1.375261
C	-1.359548	-0.590801	-0.583968	H	0.597706	4.162771	0.408404
H	1.499251	-1.228795	-2.331154	H	0.852651	4.07856	2.164558
H	-1.34742	0.044223	1.483502	C	-0.951909	5.126296	1.581277
C	2.929062	-0.386752	-0.143064	H	-0.476295	6.110806	1.549859
C	3.574964	0.632638	0.57008	H	-1.711542	5.091492	0.79482
C	3.70706	-1.426819	-0.667226	H	-1.440496	5.012823	2.552851
C	4.950353	0.593398	0.783441	H	-0.958522	-3.776085	-2.106053

H	2.999459	1.473582	0.94495	H	-1.570358	0.172316	-3.782264
C	5.082477	-1.468199	-0.454417	H	2.90923	-3.931159	0.650105
H	3.232615	-2.224841	-1.230321	H	-1.389253	-3.827647	0.335065
C	5.708931	-0.461761	0.278835	C	0.7705	-3.97114	0.350426
H	5.431133	1.394408	1.336051	H	0.828387	-4.529449	-0.577484
H	5.66435	-2.289952	-0.859873	C	3.056461	-2.402231	2.943932
H	6.780835	-0.493146	0.445328	H	3.889221	-3.096928	2.831743
C	-1.25025	-0.854285	-3.981111	H	3.362648	-1.438553	2.520758
H	-0.224566	-0.829736	-4.362287	H	2.84741	-2.266432	4.006873
H	-1.895368	-1.278224	-4.756844	C	-1.810135	-2.491926	2.71208
C	-0.86258	-3.14245	-2.991854	H	-2.137067	-3.292097	3.383259
H	0.1823	-3.153693	-3.317623	H	-1.699391	-1.579317	3.302574
H	-1.469625	-3.583392	-3.788283	H	-2.589355	-2.340107	1.962995
C	0.65308	0.098372	0.686638	N	0.63438	-2.579109	2.656729
H	1.085478	0.456812	1.614556	H	0.583139	-2.049402	3.521598
C	1.937679	-3.642245	1.02915				

Table S17. Cartesian coordinates of the optimized structure for IM5 and IM6 (S_0) of the 4-position phosphonylation.

Symbol	X	Y	Z	Symbol	X	Y	Z
C	-4.317843	-1.645002	-0.447948	H	0.911456	-2.925972	-3.406238
C	-4.342142	-2.296226	-1.684018	H	-0.715296	-3.570362	-3.681904
C	-3.199699	-2.328905	-2.492788	C	1.320698	0.459815	0.485981
C	-2.046312	-1.700726	-2.053132	H	1.788099	0.881268	1.369208
C	-2.024577	-1.028217	-0.814369	P	-0.783326	2.132166	0.23089
C	-3.161219	-1.00722	-0.002569	O	-0.536197	2.703725	-1.117723
H	-5.208735	-1.635838	0.172561	O	-2.354706	2.156513	0.571343
H	-5.252327	-2.785242	-2.016824	O	-0.056904	2.882077	1.451964
H	-3.222787	-2.845125	-3.448824	C	-2.866395	2.05093	1.915694
H	-3.148738	-0.512039	0.961898	H	-2.464461	2.874168	2.511653
C	-0.68854	-1.614385	-2.729149	H	-2.526972	1.10933	2.363532
C	0.109548	-0.839365	-1.690079	C	-4.375909	2.108115	1.843427
C	1.468943	-0.533501	-1.726099	H	-4.792344	2.023708	2.851029
C	2.080625	0.082297	-0.583402	H	-4.702697	3.0565	1.409571
C	-0.18205	0.394011	0.476083	H	-4.7689	1.290844	1.232949
C	-0.696219	-0.479421	-0.620759	C	0.32528	4.275369	1.364363

H	2.07709	-0.795597	-2.586236	H	0.703338	4.471829	0.357809
H	-0.564637	0.08532	1.459704	H	1.145827	4.381225	2.075712
C	3.555277	0.276439	-0.558963	C	-0.829445	5.193378	1.712374
C	4.10958	1.464293	-0.065476	H	-0.490996	6.232838	1.671813
C	4.418359	-0.721241	-1.029889	H	-1.654717	5.07689	1.003681
C	5.490202	1.646014	-0.033449	H	-1.19746	4.992192	2.722192
H	3.45203	2.258469	0.276291	H	-0.025348	-3.577853	-2.045852
C	5.798984	-0.540208	-0.998861	H	-1.192257	0.172001	-3.878604
H	4.00766	-1.653651	-1.406725	H	7.415957	0.786188	-0.477512
C	6.340073	0.643985	-0.499514	C	-0.77295	-0.823965	-4.047564
H	5.901672	2.575932	0.346819	H	0.22207	-0.71051	-4.489535
H	6.45295	-1.327556	-1.360814	H	-1.407868	-1.351966	-4.766098
C	-0.092555	-3.009206	-2.978079				

Table S18. Cartesian coordinates of the optimized structure for Pro (S_0) of the 4-position phosphonylation.

Symbol	X	Y	Z	Symbol	X	Y	Z
C	-5.047442	-1.326279	-0.552966	H	-1.458975	-3.435114	-3.630848
C	-5.028325	-1.824992	-1.8571	C	0.715189	-0.115318	1.005457
C	-3.821689	-1.938167	-2.548266	H	1.18079	0.216224	1.926752
C	-2.645856	-1.551285	-1.919541	P	-1.632515	0.18416	2.429768
C	-2.660279	-1.048978	-0.607816	O	-2.678474	1.216326	2.206602
C	-3.869579	-0.933906	0.08086	O	-2.253645	-1.171195	3.002446
H	-5.991053	-1.238959	-0.023587	O	-0.539417	0.556322	3.545098
H	-5.955989	-2.124196	-2.335073	C	-1.470755	-2.377839	3.173541
H	-3.805838	-2.324816	-3.563588	H	-1.580638	-2.658991	4.22271
H	-3.910725	-0.537661	1.087193	H	-0.413578	-2.16832	2.984133
C	-1.239466	-1.599142	-2.483225	C	-1.988667	-3.4565	2.24628
C	-0.437883	-1.069185	-1.30909	H	-1.443881	-4.387288	2.429769
C	0.935822	-0.904941	-1.253496	H	-3.052888	-3.635474	2.420154
C	1.534922	-0.421143	-0.085534	H	-1.849051	-3.172163	1.199858
C	-0.676072	-0.258073	0.960031	C	-0.039002	1.909315	3.674752
C	-1.264256	-0.745599	-0.220187	H	-0.170006	2.445134	2.729529
H	1.548744	-1.139667	-2.119926	H	1.031457	1.804414	3.866447
C	3.006563	-0.242795	0.005972	C	-0.74016	2.624776	4.809502
C	3.553125	0.875585	0.648987	H	-0.310531	3.623471	4.932508

C	3.877562	-1.188969	-0.549387	H	-1.807026	2.727384	4.597001
C	4.933003	1.042503	0.73553	H	-0.614953	2.075513	5.74635
H	2.894229	1.631771	1.066364	H	-0.917049	-3.694341	-1.960251
C	5.257543	-1.021716	-0.464431	H	-1.388049	0.338502	-3.478485
H	3.473323	-2.073071	-1.033931	H	-0.060051	-0.682149	-4.069057
C	5.790442	0.094513	0.178851	H	-1.730469	-1.050114	-4.530458
H	5.338238	1.919064	1.231344	C	-0.824382	-3.03948	-2.831803
H	5.917064	-1.768881	-0.894765	H	0.213621	-3.067008	-3.178148
H	6.865888	0.224882	0.245464	C	-1.095905	-0.688595	-3.715397

Table S19. Cartesian coordinates of the optimized structure for IM1 (S_1) of the 3-position phosphonylation.

Symbol	X	Y	Z	Symbol	X	Y	Z
C	-4.699623	-2.965488	-1.186974	H	-4.550694	1.509533	0.478104
C	-5.050815	-3.192528	0.153342	H	-1.162933	4.142068	1.058246
C	-4.332264	-2.612389	1.203462	C	-2.877807	2.816485	0.97439
C	-3.262402	-1.788331	0.900109	H	-3.091106	2.865557	2.034089
C	-2.918349	-1.543388	-0.451056	N	-2.362224	2.735247	-1.72872
C	-3.629516	-2.144379	-1.503879	H	-2.180825	2.714177	-2.721829
H	-5.272934	-3.438461	-1.976758	C	-0.432092	4.237686	-1.600713
H	-5.893046	-3.838967	0.377508	H	-0.831016	4.980016	-2.301242
H	-4.613438	-2.812125	2.232761	H	0.189825	4.752017	-0.866514
H	-3.352843	-1.965637	-2.537965	H	0.212717	3.558726	-2.173251
C	-2.308588	-1.078588	1.847651	C	-4.256077	1.282475	-2.249598
C	-1.464698	-0.288028	0.859819	H	-4.749223	1.973632	-2.941946
C	-0.46004	0.598599	1.106343	H	-3.624005	0.615251	-2.848583
C	0.296658	1.138462	0.018393	H	-5.019888	0.679955	-1.756592
C	-1.031209	-0.176339	-1.570642	P	2.990904	-1.418789	-0.852061
C	-1.808319	-0.643654	-0.483647	O	4.49386	-1.296848	-1.455056
H	-0.199349	0.852187	2.127365	O	3.372828	-1.235034	0.743687
H	-1.24829	-0.491296	-2.585033	O	2.720628	-3.053428	-0.836968
C	1.395215	2.056896	0.259309	C	5.632257	-2.003477	-0.913665
C	2.418527	2.24786	-0.697237	H	5.291371	-2.796166	-0.242425
C	1.472639	2.794062	1.464082	H	6.13122	-2.467063	-1.768447
C	3.46111	3.130359	-0.459205	C	6.554321	-1.037018	-0.197449
H	2.423717	1.674992	-1.616882	H	6.044933	-0.585013	0.657026

C	2.509267	3.68353	1.690924	H	7.439802	-1.570022	0.163202
H	0.695329	2.697223	2.213389	H	6.88269	-0.242884	-0.87398
C	3.510909	3.855807	0.731537	C	2.34142	-1.491999	1.70342
H	4.243148	3.247525	-1.201606	H	2.372077	-2.55226	1.976089
H	2.538625	4.24911	2.616038	H	1.356441	-1.293804	1.260775
H	4.325568	4.548667	0.913836	C	2.561216	-0.602428	2.908502
C	-1.413763	-2.10826	2.571812	H	1.78773	-0.792053	3.659321
H	-0.681013	-1.593295	3.200704	H	3.536351	-0.799708	3.361951
H	-2.024097	-2.751578	3.211922	H	2.519094	0.452221	2.620578
C	-3.035373	-0.206854	2.879405	C	1.603693	-3.55962	-1.584541
H	-2.314943	0.371229	3.465743	H	1.901468	-4.549307	-1.93804
H	-3.601074	-0.839609	3.56935	H	1.421149	-2.929854	-2.464169
C	0.000604	0.680816	-1.312797	C	0.361753	-3.638409	-0.716335
H	0.586531	1.052186	-2.143436	H	-0.471356	-4.071949	-1.278157
C	-1.807357	3.543224	0.424751	H	0.546916	-4.254676	0.168087
C	-1.530975	3.492944	-0.919946	H	0.063501	-2.638113	-0.386798
C	-3.44161	2.02217	-1.241742	H	-0.877305	-2.740296	1.858632
C	-3.700189	2.068	0.103193	H	-3.728469	0.486259	2.401492

Table S20. Cartesian coordinates of the optimized structure for IM2 (S_1) of the 3-position phosphonylation.

Symbol	X	Y	Z	Symbol	X	Y	Z
C	-5.307443	-1.253389	-1.027648	H	-3.048624	3.292436	-0.135496
C	-5.842772	-0.939714	0.224843	H	1.178285	4.225236	-0.016081
C	-5.02287	-0.46722	1.252297	C	-0.933894	3.758589	0.167285
C	-3.665969	-0.29868	1.007848	H	-1.071225	4.268133	1.111943
C	-3.13412	-0.600544	-0.253644	N	-0.590778	2.455942	-2.244877
C	-3.945392	-1.090563	-1.274373	H	-0.496737	2.121502	-3.192274
H	-5.957087	-1.627224	-1.812627	C	1.778637	3.065474	-2.444888
H	-6.905676	-1.070739	0.401957	H	1.688727	3.6007	-3.398118
H	-5.446105	-0.237606	2.226258	H	2.586957	3.518274	-1.868409
H	-3.530718	-1.341558	-2.246634	H	2.074284	2.034565	-2.679526
C	-2.582236	0.156935	1.977143	C	-2.969417	2.02398	-2.579554
C	-1.376833	0.204659	1.060178	H	-3.063508	2.573767	-3.523555
C	-0.117758	0.719943	1.328492	H	-2.762421	0.975824	-2.827451
C	0.921262	0.620573	0.411136	H	-3.925383	2.064767	-2.055479

C	-0.730904	-0.508937	-1.169589	P	1.51066	-1.919355	-0.433657
C	-1.688792	-0.336218	-0.228751	O	2.584544	-2.116374	-1.558154
H	0.041264	1.237139	2.268367	O	2.261902	-1.9576	0.940585
H	-0.948291	-0.868905	-2.168792	O	0.501263	-3.119309	-0.344497
C	2.223209	1.259174	0.614432	C	3.662957	-3.112969	-1.485826
C	3.337516	0.958492	-0.190369	H	3.369985	-3.904076	-0.791935
C	2.407211	2.218796	1.630624	H	3.717347	-3.523509	-2.493334
C	4.569401	1.570793	0.014179	C	4.943811	-2.428771	-1.069147
H	3.268517	0.235386	-0.995355	H	4.855522	-2.010252	-0.06382
C	3.63614	2.831177	1.833682	H	5.754936	-3.162031	-1.071599
H	1.573304	2.517987	2.255009	H	5.199843	-1.628444	-1.767841
C	4.729871	2.511408	1.027939	C	1.596162	-2.164783	2.232536
H	5.405798	1.307856	-0.625941	H	1.627599	-3.238264	2.422761
H	3.738264	3.57129	2.621108	H	0.55579	-1.841251	2.152088
H	5.689667	2.99157	1.18712	C	2.346672	-1.367252	3.269164
C	-2.360574	-0.913165	3.068501	H	1.883106	-1.53373	4.245382
H	-1.525953	-0.624854	3.716359	H	3.391274	-1.682817	3.32249
H	-3.256741	-1.018166	3.688104	H	2.309057	-0.29975	3.039675
C	-2.900792	1.507124	2.634209	C	-0.215516	-3.733168	-1.464022
H	-2.07695	1.82425	3.281577	H	0.376317	-4.602069	-1.756286
H	-3.799127	1.423588	3.253633	H	-0.253999	-3.03293	-2.300473
C	0.705716	-0.277302	-0.794782	C	-1.593319	-4.110948	-0.980284
H	1.263522	0.08974	-1.662984	H	-2.134316	-4.601191	-1.794076
C	0.32017	3.746453	-0.474159	H	-1.53022	-4.805048	-0.138963
C	0.502704	3.108078	-1.670803	H	-2.153876	-3.225159	-0.672695
C	-1.877793	2.589437	-1.733983	H	-2.134547	-1.886449	2.621245
C	-2.043109	3.211758	-0.533254	H	-3.06562	2.281683	1.883708

Table S21. Cartesian coordinates of the optimized structure for IM3 (S_0) of the 3-position phosphonylation.

Symbol	X	Y	Z	Symbol	X	Y	Z
C	-5.254384	-1.369298	0.099659	H	-1.645818	2.333355	3.444675
C	-5.6257	-0.976256	1.389267	H	-3.357822	1.932759	3.647439
C	-4.699421	-0.385285	2.250697	C	0.660386	-0.120672	-0.67379
C	-3.397201	-0.190427	1.806613	H	1.001323	0.347928	-1.607432
C	-3.029478	-0.585496	0.513719	P	1.635953	-1.685628	-0.597532

C	-3.948996	-1.177854	-0.348751	O	3.039681	-1.348173	-1.185351
H	-5.988557	-1.827248	-0.555309	O	1.845426	-2.191051	0.865151
H	-6.646437	-1.132649	1.723764	O	0.924567	-2.870023	-1.347578
H	-4.999143	-0.083605	3.250387	C	4.2671	-2.120185	-0.941518
H	-3.659528	-1.483937	-1.349804	H	3.99476	-3.154983	-0.723758
C	-2.226107	0.425918	2.561859	H	4.793824	-2.081558	-1.894083
C	-1.12765	0.333286	1.519507	C	5.050197	-1.476417	0.177527
C	0.171443	0.84838	1.58136	H	4.505961	-1.533466	1.122718
C	1.060544	0.709715	0.534877	H	6.002523	-2.002406	0.289738
C	-0.802803	-0.461456	-0.760807	H	5.253119	-0.42684	-0.049095
C	-1.605799	-0.277073	0.317878	C	0.805901	-2.780471	1.718425
H	0.476819	1.424882	2.450799	H	0.794477	-3.84882	1.499689
H	-1.179125	-0.876059	-1.689605	H	-0.15887	-2.343596	1.452316
C	2.420036	1.28012	0.597746	C	1.182804	-2.481882	3.14733
C	3.034686	1.842503	-0.531909	H	0.448105	-2.942327	3.813108
C	3.131638	1.275069	1.808294	H	2.168124	-2.890721	3.382683
C	4.307935	2.396039	-0.448755	H	1.187418	-1.404175	3.327994
H	2.512437	1.872426	-1.483012	C	0.668922	-2.887094	-2.790657
C	4.405911	1.826127	1.889969	H	1.561667	-3.303782	-3.25949
H	2.693924	0.810201	2.686311	H	0.526121	-1.863141	-3.146778
C	4.999553	2.390698	0.762016	C	-0.555594	-3.735835	-3.028393
H	4.759621	2.835508	-1.332277	H	-0.750663	-3.783847	-4.102806
H	4.941146	1.802675	2.833787	H	-0.399315	-4.751495	-2.658226
H	5.995054	2.818059	0.824142	H	-1.431395	-3.307782	-2.534148
C	-1.874996	-0.396339	3.815871	H	-1.691055	-1.443912	3.559897
H	-0.979564	0.00617	4.299935	H	-2.760195	2.485729	2.069753
H	-2.697289	-0.359194	4.537099	C	-2.514768	1.886814	2.951296

Table S22. Cartesian coordinates of the optimized structure for IM4 (S_0) of the 3-position phosphonylation.

Symbol	X	Y	Z	Symbol	X	Y	Z
C	-5.009928	-0.692845	0.077803	C	-1.762499	3.065438	-1.133229
C	-5.330228	-0.075958	1.289773	C	-3.494273	1.836147	-2.259263
C	-4.327498	0.440744	2.114384	C	-4.448776	2.411475	-1.435288
C	-3.00397	0.354839	1.701512	P	1.731051	-2.083594	-0.466454

C	-2.690316	-0.228081	0.465739	O	3.22739	-1.892838	-1.001763
C	-3.683309	-0.776183	-0.34158	O	1.580102	-2.658423	0.894315
H	-5.799158	-1.108348	-0.540878	O	0.994418	-3.018273	-1.549685
H	-6.368049	-0.012545	1.601614	C	4.326002	-2.709616	-0.526481
H	-4.585348	0.893215	3.067961	H	3.940293	-3.66053	-0.14944
H	-3.43227	-1.264546	-1.278756	H	4.935829	-2.906853	-1.410213
C	-1.746133	0.765801	2.458843	C	5.105466	-1.966177	0.536446
C	-0.679144	0.4943	1.414796	H	4.485914	-1.791761	1.419535
C	0.670023	0.853743	1.434913	H	5.976829	-2.558585	0.83179
C	1.524452	0.519765	0.400017	H	5.448418	-0.999524	0.157573
C	-0.467622	-0.506874	-0.795821	C	1.187026	-2.842413	-2.968683
C	-1.236197	-0.146274	0.263746	H	2.235679	-3.040528	-3.207194
H	1.042816	1.473978	2.245604	H	0.960341	-1.805378	-3.243066
H	-0.895725	-0.968853	-1.680345	C	0.261637	-3.799047	-3.686601
C	2.9172	1.003233	0.383413	H	0.39233	-3.691702	-4.766991
C	3.560829	1.348526	-0.815986	H	0.486564	-4.832308	-3.410895
C	3.634786	1.152642	1.582122	H	-0.78198	-3.588211	-3.43865
C	4.863056	1.83738	-0.815572	H	-1.519954	-1.212896	3.366491
H	3.04026	1.251941	-1.763626	H	-1.89367	2.909129	2.085735
C	4.937591	1.638735	1.582506	C	-3.801841	0.883456	-3.367406
H	3.180026	0.851467	2.520637	H	-4.726864	0.347225	-3.154566
C	5.559311	1.985283	0.383463	H	-3.933637	1.437223	-4.302283
H	5.335224	2.104107	-1.755972	H	-2.998129	0.157413	-3.511375
H	5.47439	1.731658	2.521375	C	-0.293629	3.322055	-1.069759
H	6.57785	2.359875	0.382515	H	-0.054981	3.947898	-0.210554
C	-1.542492	-0.164099	3.675726	H	0.256079	2.381298	-0.970406
H	-0.59719	0.069798	4.17535	H	0.046331	3.828314	-1.978029
H	-2.356265	-0.030722	4.395799	H	-2.391145	4.381775	0.421736
C	-1.773635	2.226027	2.928515	H	-5.488027	2.135277	-1.557071
H	-0.845332	2.480289	3.44937	C	-4.052903	3.321315	-0.463707
H	-2.602044	2.388149	3.625168	H	-4.79388	3.772947	0.186204
C	1.027707	-0.38176	-0.712154	N	-2.204218	2.185625	-2.064127
H	1.43679	-0.069344	-1.681878	H	-1.505588	1.746552	-2.656303
C	-2.708557	3.65954	-0.319233				

Table S23. Cartesian coordinates of the optimized structure for IM5 and IM6 (S_0) of the 3-position phosphonylation.

Symbol	X	Y	Z	Symbol	X	Y	Z
C	-5.320777	-1.218789	0.432732	H	-1.380485	2.396725	3.498703
C	-5.595072	-0.795086	1.737225	H	-3.089126	2.049559	3.798705
C	-4.599088	-0.22359	2.529891	C	0.572995	-0.167045	-0.638332
C	-3.323935	-0.080969	1.998201	H	0.953863	0.174484	-1.607606
C	-3.053895	-0.507109	0.692904	P	1.393426	-1.88444	-0.484842
C	-4.043131	-1.079415	-0.103097	O	2.854205	-1.540891	-0.98821
H	-6.110335	-1.660944	-0.165606	O	1.227698	-2.486314	0.854104
H	-6.596536	-0.913315	2.138037	O	0.69224	-2.731497	-1.631051
H	-4.823515	0.100832	3.541599	C	4.020722	-2.323557	-0.581512
H	-3.829898	-1.409353	-1.114983	H	3.696394	-3.323664	-0.2853
C	-2.0875	0.505172	2.663562	H	4.622202	-2.400863	-1.487677
C	-1.062244	0.374441	1.562349	C	4.757062	-1.617158	0.531556
C	0.247922	0.829281	1.592398	H	4.13744	-1.549155	1.42902
C	1.076049	0.642545	0.493661	H	5.661074	-2.182116	0.776641
C	-0.868389	-0.464644	-0.68475	H	5.048619	-0.61001	0.223914
C	-1.639367	-0.243663	0.409204	C	0.979379	-2.565796	-3.046969
H	0.598808	1.39169	2.449727	H	2.051462	-2.705303	-3.199927
H	-1.266692	-0.930909	-1.579575	H	0.70842	-1.54716	-3.344028
C	2.429665	1.19936	0.471448	C	0.16165	-3.586999	-3.801522
C	3.025158	1.615445	-0.731615	H	0.361549	-3.484276	-4.871396
C	3.146533	1.352429	1.671852	H	0.42846	-4.600526	-3.493384
C	4.292825	2.181926	-0.729677	H	-0.906451	-3.432298	-3.631406
H	2.49206	1.534185	-1.672308	H	-1.474006	-1.369043	3.615999
C	4.418713	1.906552	1.66728	H	-2.570886	2.58614	2.189626
H	2.727105	0.997077	2.606695	C	-1.647691	-0.324955	3.888465
C	4.9923	2.327836	0.467737	H	-0.731933	0.084681	4.323768
H	4.734057	2.513443	-1.66311	H	-2.434447	-0.289955	4.64686
H	4.966934	2.001918	2.597994	C	-2.290005	1.981592	3.055962
H	5.984611	2.766336	0.465579				

Table S24. Cartesian coordinates of the optimized structure for Pro (S_0) of the 3-position phosphonylation.

Symbol	X	Y	Z	Symbol	X	Y	Z
C	-4.983278	-1.964651	0.279524	H	-1.954506	2.812197	2.699126

C	-5.480112	-1.309589	1.409809	H	-3.635564	2.307782	2.945574
C	-4.674448	-0.427111	2.134159	C	0.723773	-0.218586	-0.571896
C	-3.369266	-0.209165	1.713208	P	1.85024	-0.675339	-1.910019
C	-2.874345	-0.86725	0.577629	O	2.384157	0.752755	-2.399874
C	-3.672899	-1.748397	-0.14693	O	2.881284	-1.70328	-1.606245
H	-5.622907	-2.647853	-0.27037	O	0.906462	-1.206747	-3.103416
H	-6.502333	-1.489255	1.728106	C	3.510327	0.854751	-3.303373
H	-5.069318	0.077336	3.011881	H	3.600249	-0.065347	-3.889344
H	-3.288116	-2.259707	-1.024709	H	3.255432	1.670946	-3.982513
C	-2.310994	0.695689	2.330225	C	4.779995	1.147039	-2.533177
C	-1.141962	0.463746	1.38562	H	5.036708	0.310787	-1.879291
C	0.129333	1.011369	1.431039	H	5.603273	1.313621	-3.234666
C	1.085487	0.668849	0.464543	H	4.655266	2.040616	-1.915793
C	-0.559306	-0.781962	-0.602982	C	0.04962	-0.309541	-3.843578
C	-1.482416	-0.443074	0.375388	H	0.667372	0.239393	-4.560044
H	0.410203	1.697531	2.225706	H	-0.408319	0.412283	-3.158843
H	-0.829444	-1.471688	-1.396109	C	-1.006431	-1.13638	-4.542675
C	2.450596	1.252027	0.610861	H	-1.650018	-0.480257	-5.135331
C	2.630047	2.639131	0.569807	H	-0.545919	-1.867942	-5.211531
C	3.553975	0.43268	0.866939	H	-1.629603	-1.66742	-3.817558
C	3.890578	3.195686	0.769688	H	-1.642301	-0.787377	3.786772
H	1.777521	3.282087	0.37011	H	-2.999236	2.493189	1.298615
C	4.81381	0.990101	1.074378	H	5.969269	2.804781	1.182987
H	3.423894	-0.64387	0.907364	C	-1.963547	0.257949	3.763781
C	4.986615	2.371553	1.024653	H	-1.157359	0.87788	4.168379
H	4.016772	4.273118	0.726631	H	-2.836002	0.366743	4.415546
H	5.661179	0.341661	1.274088	C	-2.752498	2.169553	2.314017

Table S25. Cartesian coordinates of the optimized structure for Lut-H^+.

Symbol	X	Y	Z	Symbol	X	Y	Z
C	-1.202287	0.224633	-0.00002201	H	2.442622	1.72372	0.8863
C	1.202272	0.224658	-0.00003997	H	3.319746	0.464308	-0.000919
C	1.208039	-1.159186	-0.00002807	H	2.441764	1.725253	-0.885139
C	1.02E-05	-1.849197	0.00001208	C	-2.42413	1.083571	7.57E-06
C	-1.208038	-1.159199	0.00002205	H	-2.442248	1.724324	0.885846

H	2.153978	-1.684771	-0.00004376	H	-2.442109	1.724659	-0.885588
H	1.57E-05	-2.933177	0.00002759	H	-3.319755	0.464332	-0.000172
H	-2.153967	-1.684804	0.00005083	N	-2.41E-06	0.84588	-2.98E-05
C	2.424131	1.083568	0.00003058	H	-7.69E-06	1.861909	-4.77E-05

Table S26. Cartesian coordinates of the optimized structure for Lut-H•.

Symbol	X	Y	Z	Symbol	X	Y	Z
C	1.22466	-0.213031	0.001372	H	-2.635757	-1.478364	1.019814
C	-1.220867	-0.215272	-0.020898	H	-3.326553	-0.487788	-0.277591
C	-1.213309	1.146813	-0.010242	H	-2.367956	-1.918794	-0.671921
C	0.000464	1.882541	0.004144	C	2.44556	-1.074036	0.002704
C	1.215706	1.148765	0.008985	H	2.467462	-1.738659	0.877161
H	-2.168483	1.661479	-0.012761	H	2.490262	-1.710741	-0.891511
H	-0.000298	2.963819	0.009956	H	3.344997	-0.455484	0.024404
H	2.169498	1.665452	0.018586	N	0.002034	-0.891245	-0.014752
C	-2.45069	-1.069968	0.017892	H	0.003445	-1.897075	-0.016615

Table S27. Cartesian coordinates of the optimized structure for Lut.

Symbol	X	Y	Z	Symbol	X	Y	Z
C	1.154652	0.265678	1.71E-05	H	-2.443162	1.739305	-0.880474
C	-1.154587	0.265745	-1.71E-05	H	-3.307567	0.462725	-0.0006
C	-1.196948	-1.129726	-3.41E-05	H	-2.443818	1.7383	0.881213
C	-5.27E-05	-1.834707	4.08E-06	C	2.414331	1.090831	-1.64E-05
C	1.19692	-1.129705	3.28E-05	H	2.443724	1.738306	-0.881204
H	-2.15038	-1.646965	-8.77E-05	H	2.443229	1.739179	0.880534
H	1.55E-05	-2.920199	2.3E-07	H	3.307622	0.462738	0.000514
H	2.150273	-1.647096	5.4E-05	N	4.76E-06	0.9457	1.81E-05
C	-2.414309	1.090852	9.5E-07				

Table S28. Cartesian coordinates of the optimized structure for $P(OEt)_3$.

Symbol	X	Y	Z	Symbol	X	Y	Z
P	-0.409231	0.335575	0.229791	C	0.468798	-3.467058	0.430247
O	0.307106	1.004475	1.549282	H	1.562138	-3.464044	0.446529
O	0.458722	1.124517	-0.921927	H	0.116637	-4.311486	1.02997
O	0.426302	-1.080089	0.212596	H	0.133914	-3.609398	-0.600891

C	0.085034	0.941106	-2.296762	C	0.604141	2.115389	-3.099859
H	0.517665	0.000679	-2.655102	H	1.691554	2.190829	-3.013244
H	-1.007291	0.868384	-2.378983	H	0.349953	1.982944	-4.155599
C	-0.076866	-2.173146	0.997163	H	0.158854	3.050708	-2.749821
H	0.24315	-2.035633	2.035742	C	0.183873	2.37961	3.508268
H	-1.17444	-2.172941	0.974774	H	-0.186914	3.328448	3.906939
C	-0.220723	2.240841	2.055538	H	-0.233653	1.564561	4.105756
H	-1.313875	2.247493	1.955372	H	1.272675	2.364909	3.607793
H	0.181815	3.065408	1.457177				

Table S29. Cartesian coordinates of the optimized structure for C_2H_2.

Symbol	X	Y	Z	Symbol	X	Y	Z
C	0	0.664094	0	C	0	-0.664094	0
H	0.924558	1.234617	0	H	-0.924558	-1.234617	0
H	-0.924518	1.234659	0	H	0.924518	-1.234659	0

Table S30. Cartesian coordinates of the optimized structure for H_2.

Symbol	X	Y	Z	Symbol	X	Y	Z
H	0	0	0.37141257	H	0	0	-0.371413

14. NMR spectra

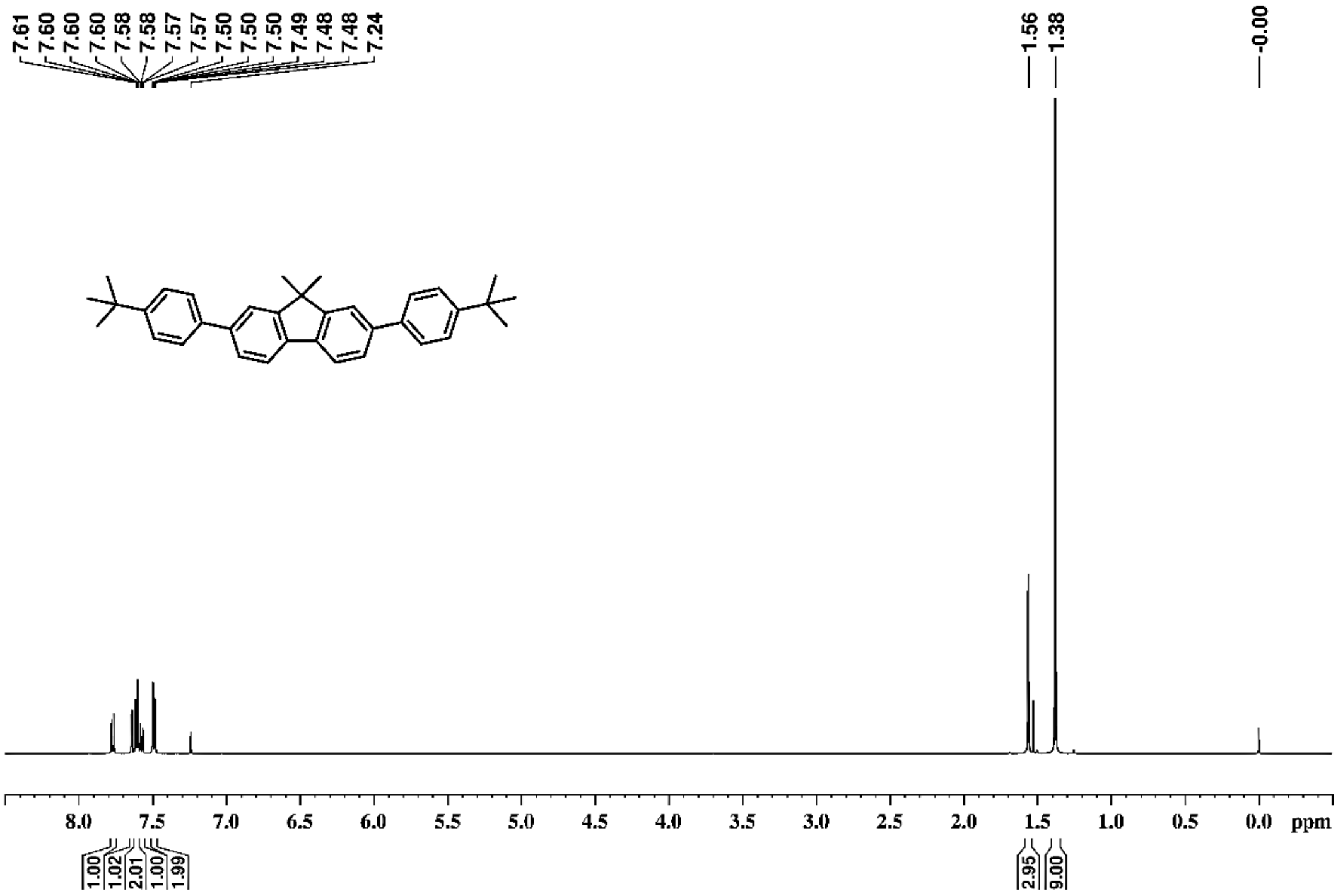

Figure S14. ^{1}H NMR spectrum (500.13 MHz, $CDCl_3$) of BPF.

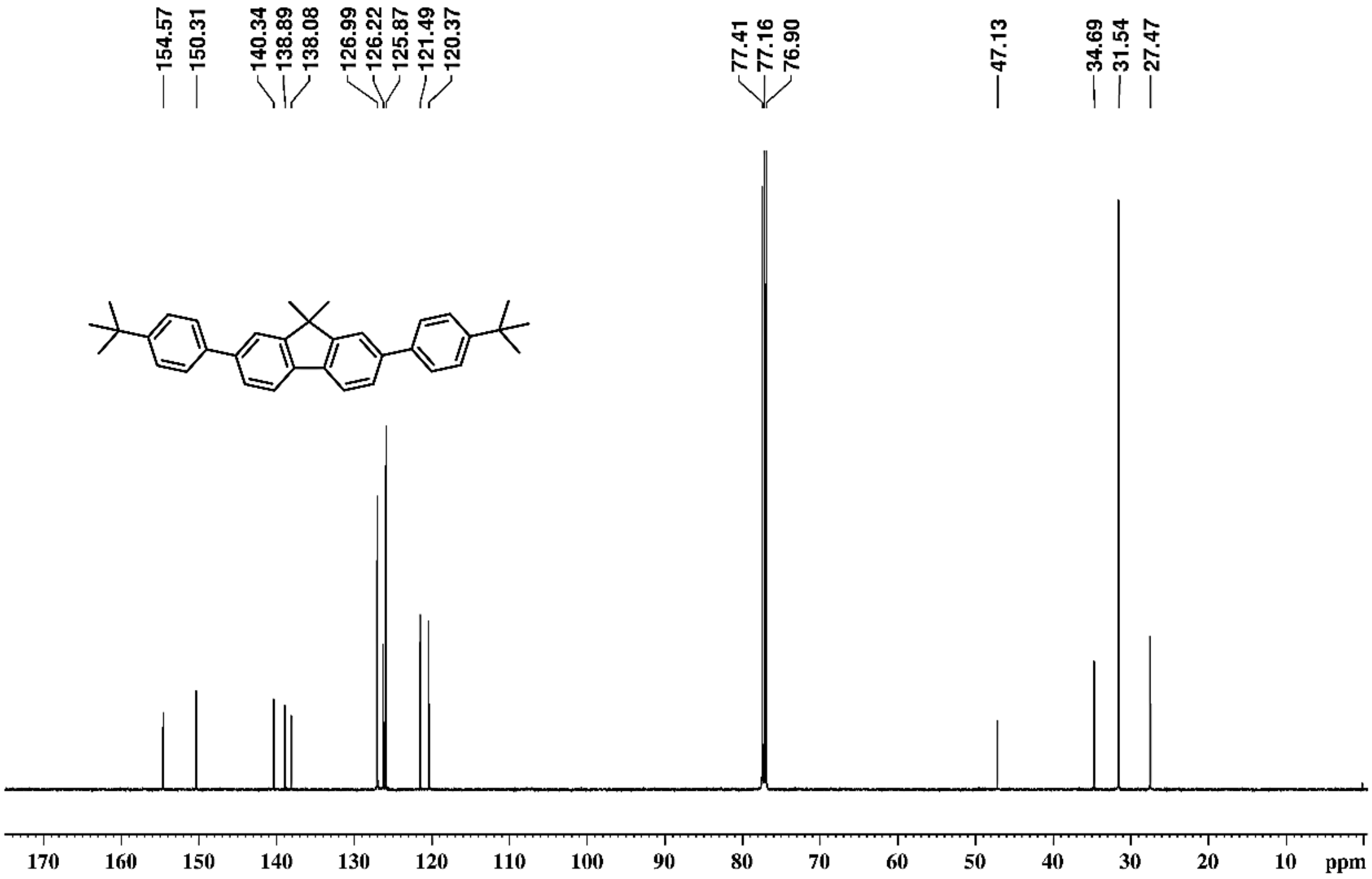

Figure S15. ^{13}C NMR spectrum (125.76 MHz, $CDCl_3$) of BPF.

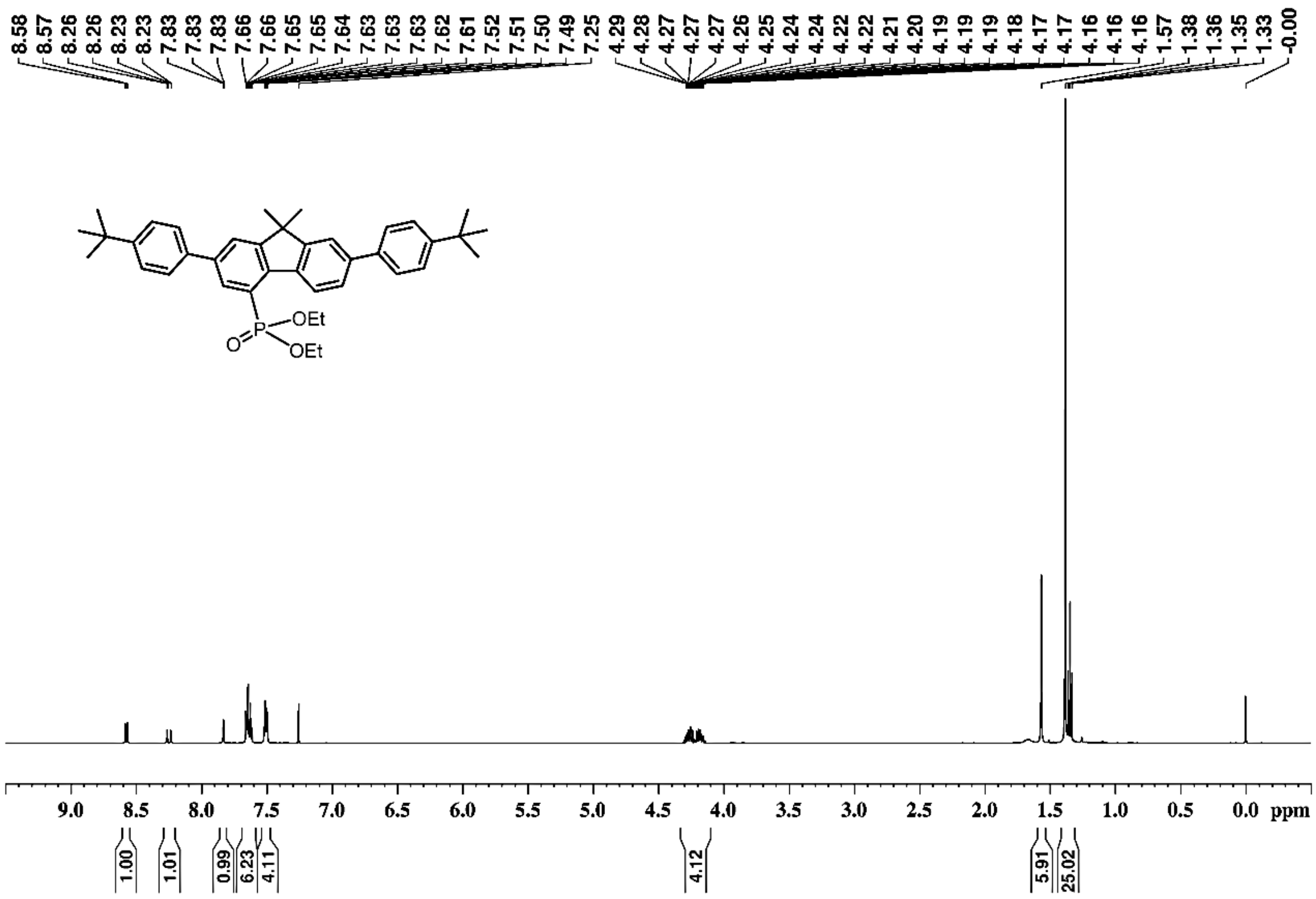

Figure S16. ^{1}H NMR spectrum (500.13 MHz, $CDCl_3$) of BPF-Phos(Et) at the 4-position.

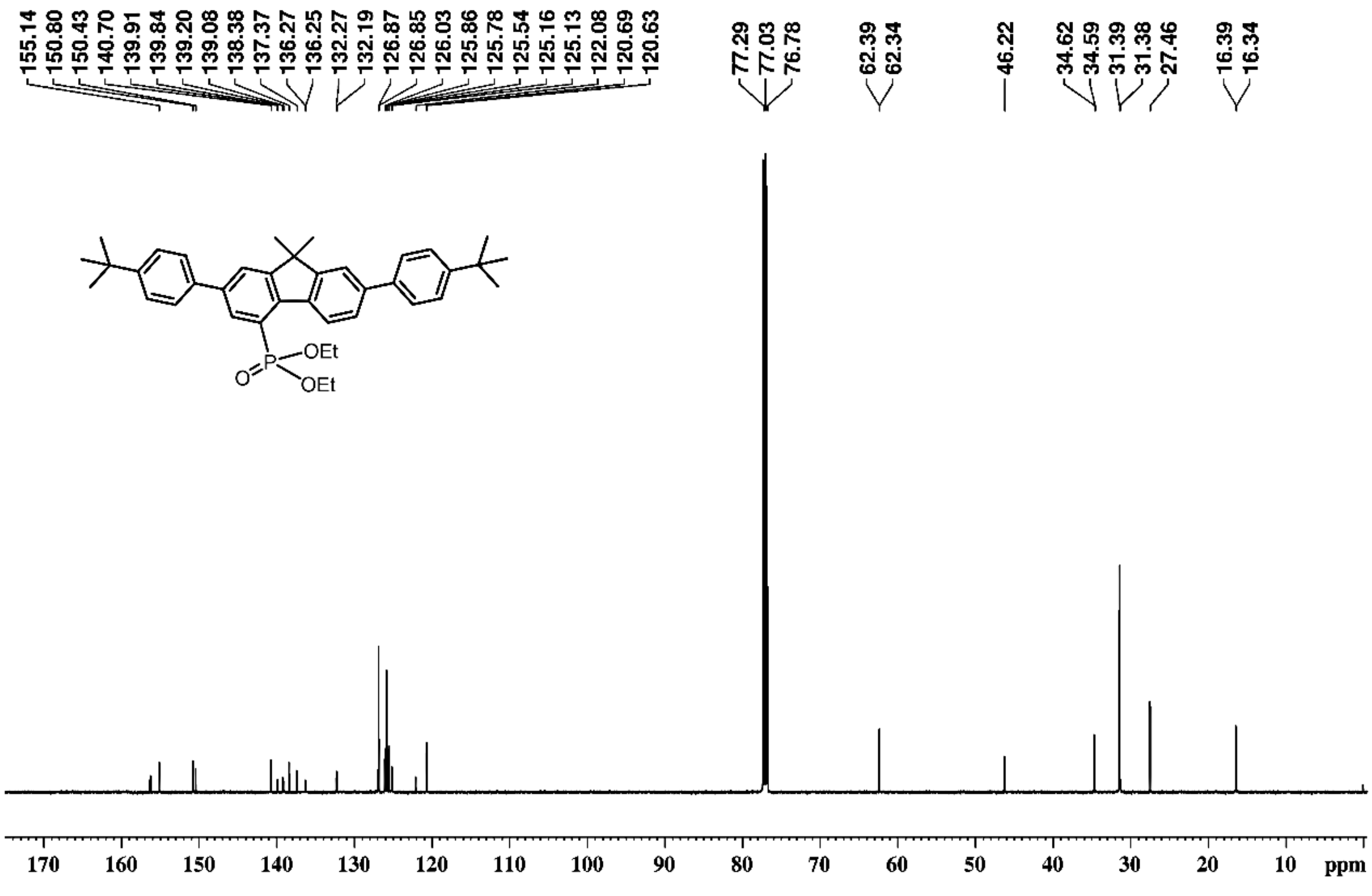

Figure S17. ^{13}C NMR spectrum (125.76 MHz, $CDCl_3$) of BPF-Phos(Et) at the 4-position.

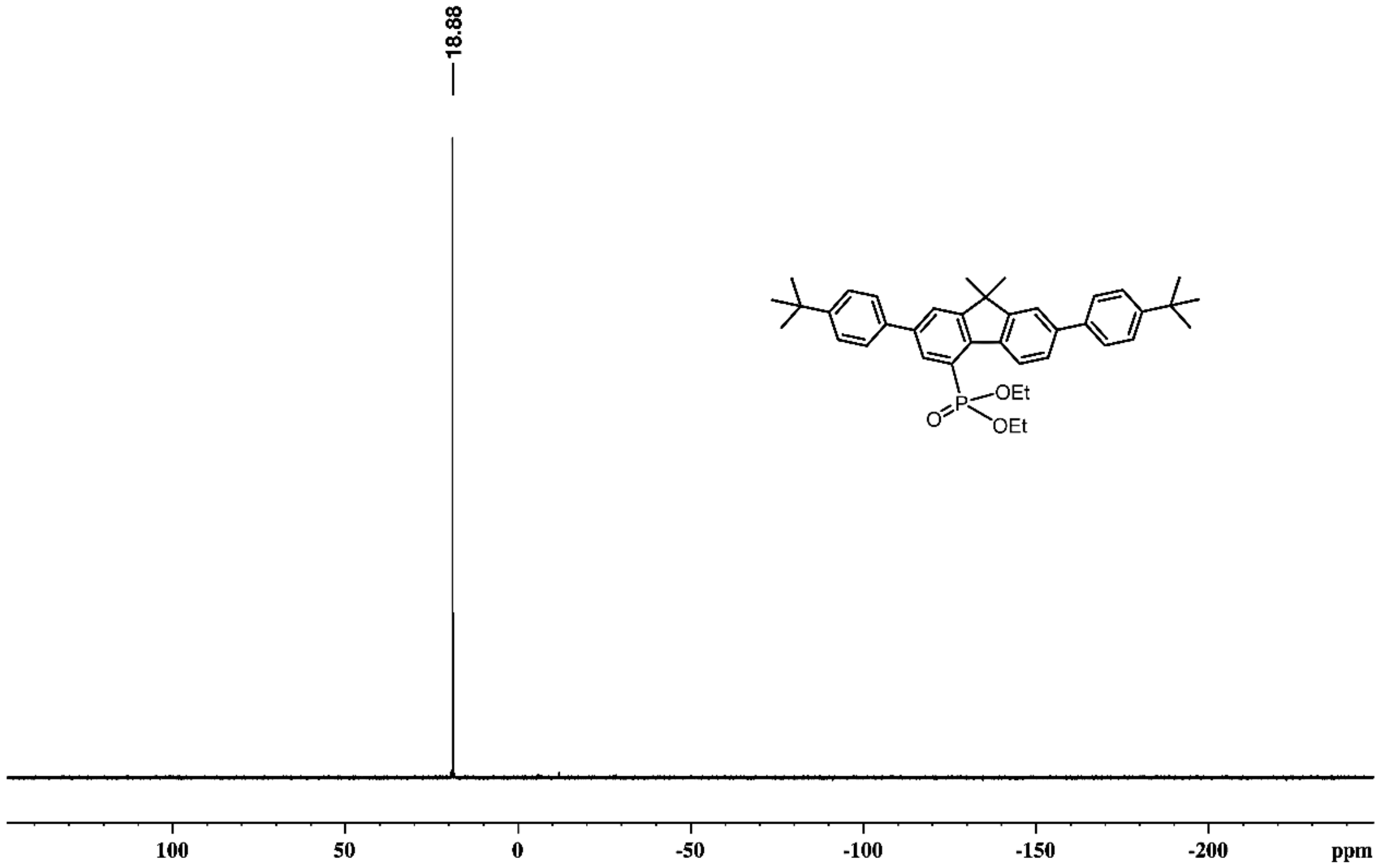

Figure S18. ^{31}P NMR spectrum (200.44 MHz, $CDCl_3$) of BPF-Phos(Et) at the 4-position.

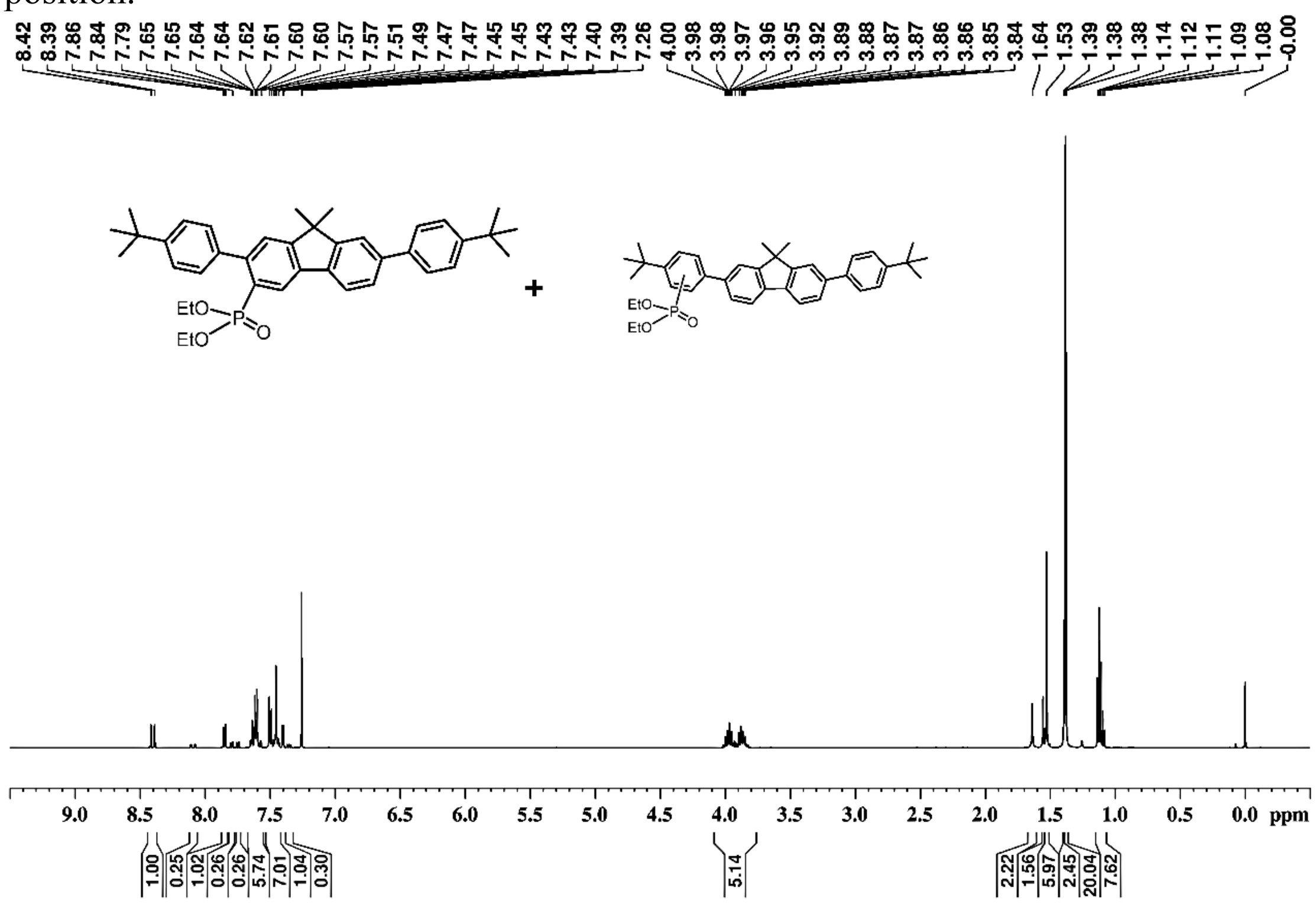

Figure S19. ^{1}H NMR spectrum (500.13 MHz, $CDCl_3$) of BPF-Phos(Et)) at the 3-and phenyl position.

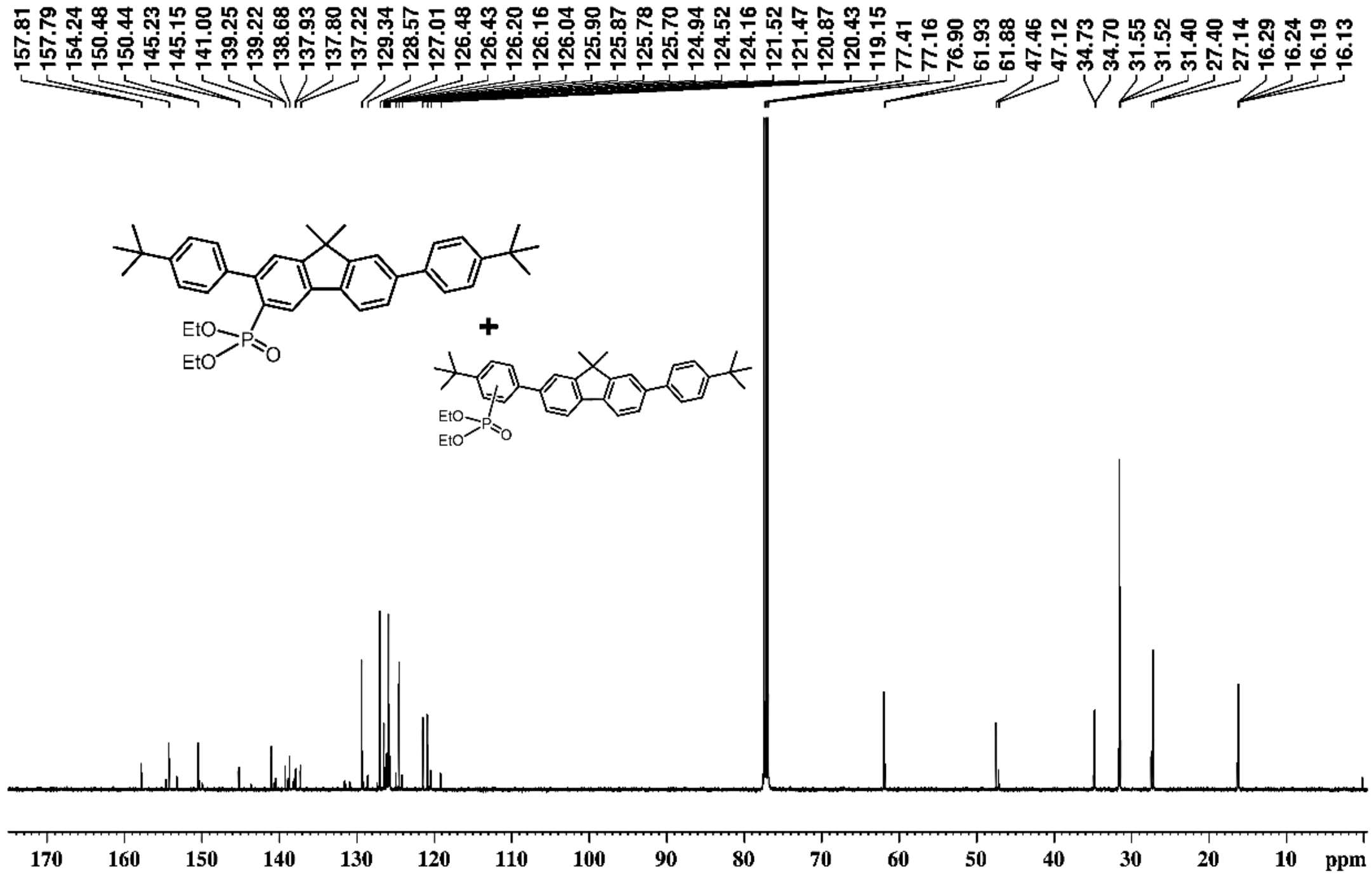

Figure S20. ^{13}C NMR spectrum (125.76 MHz, $CDCl_3$) of BPF-Phos(Et) at the 3-and phenyl position..

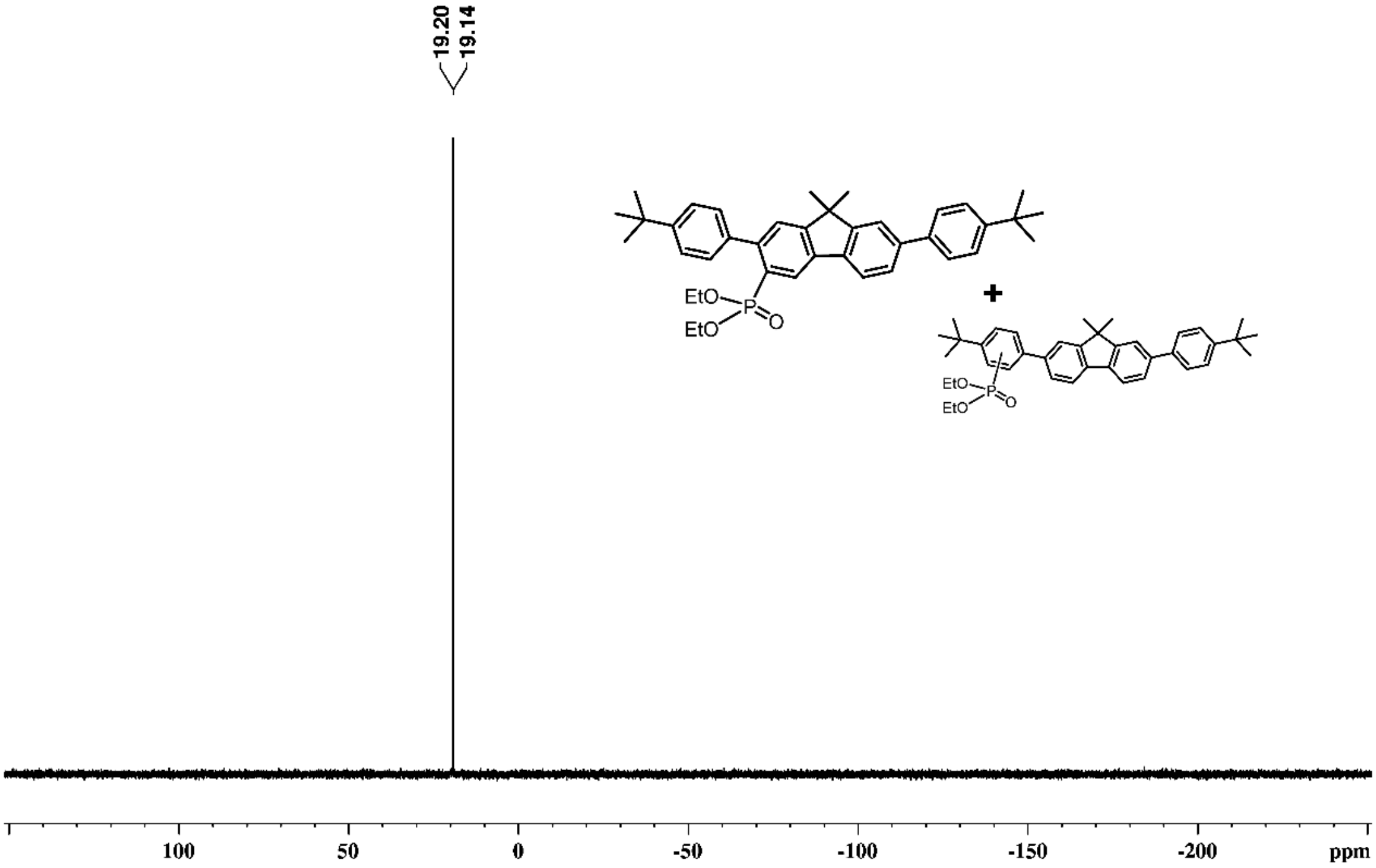

Figure S21. ^{31}P NMR spectrum (200.44 MHz, $CDCl_3$) of BPF-Phos(Et)) at the 3-and phenyl position.

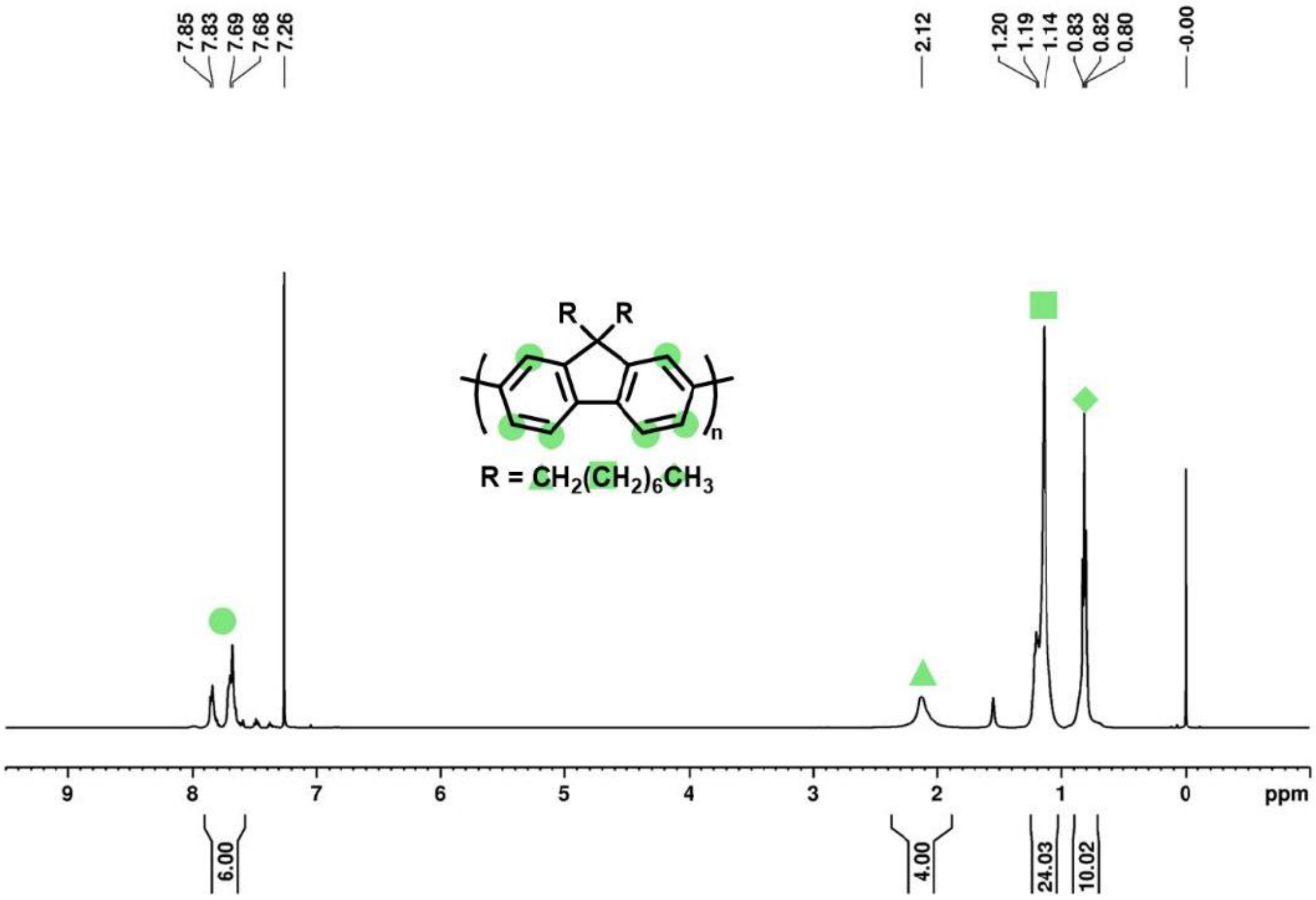

Figure S22. ^{1}H NMR spectrum (500.13 MHz, $CDCl_3$) of PFO.

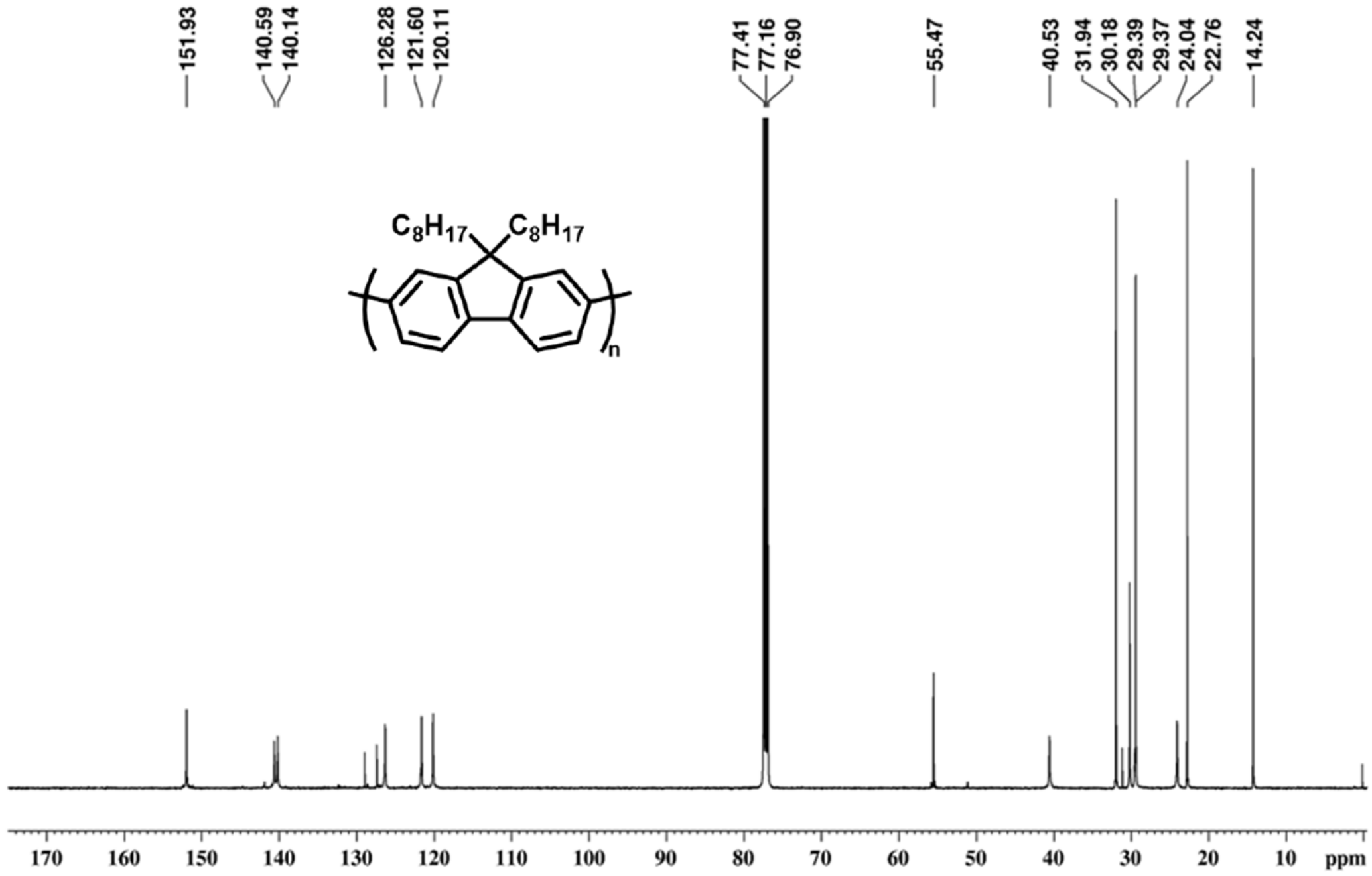

Figure S23. ^{13}C NMR spectrum (125.76 MHz, $CDCl_3$) of PFO.

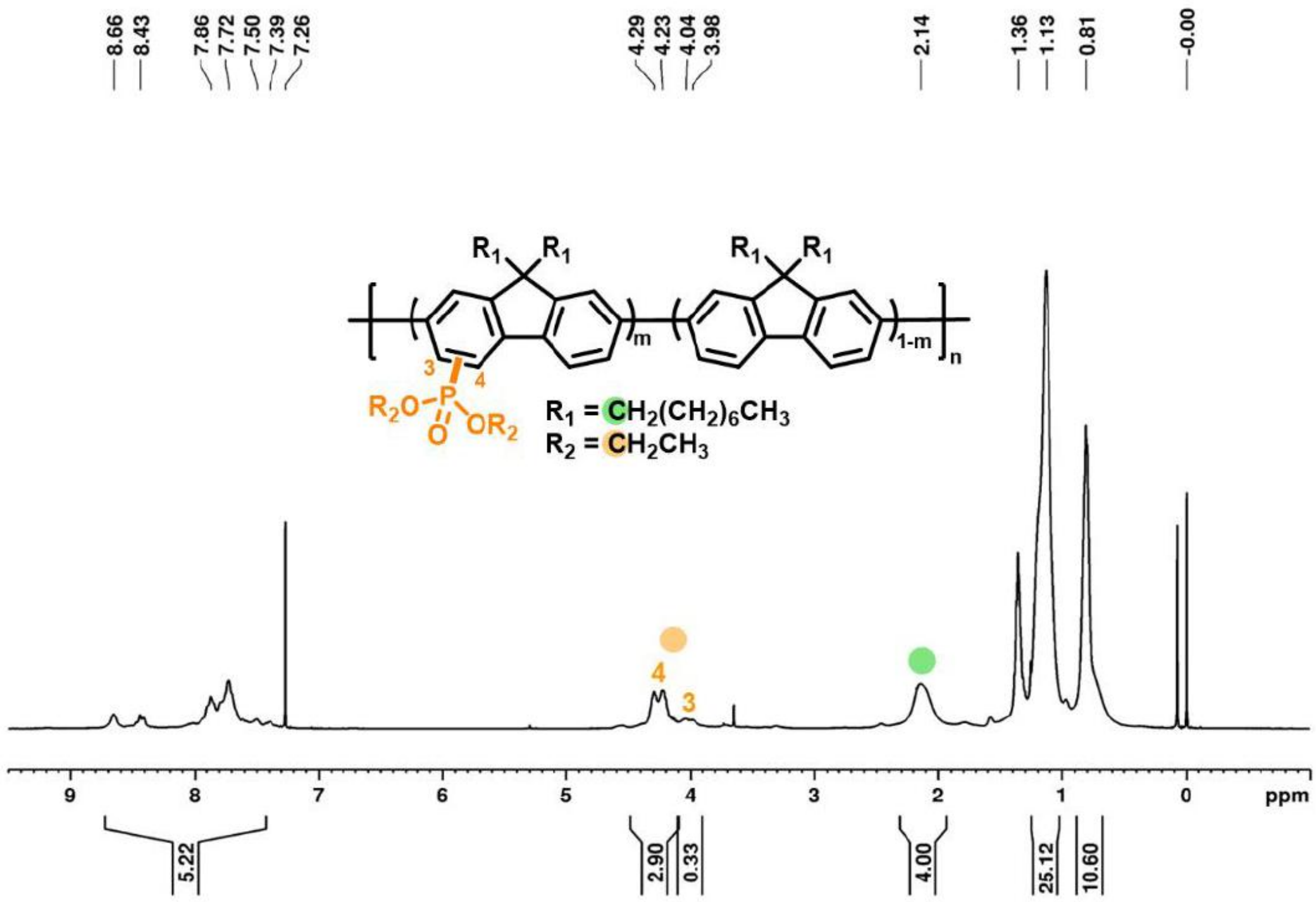

Figure S24. ^{1}H NMR spectrum (500.13 MHz, $CDCl_3$) of PFO-Phos(Et) (**P1**, DOF: 1.00).

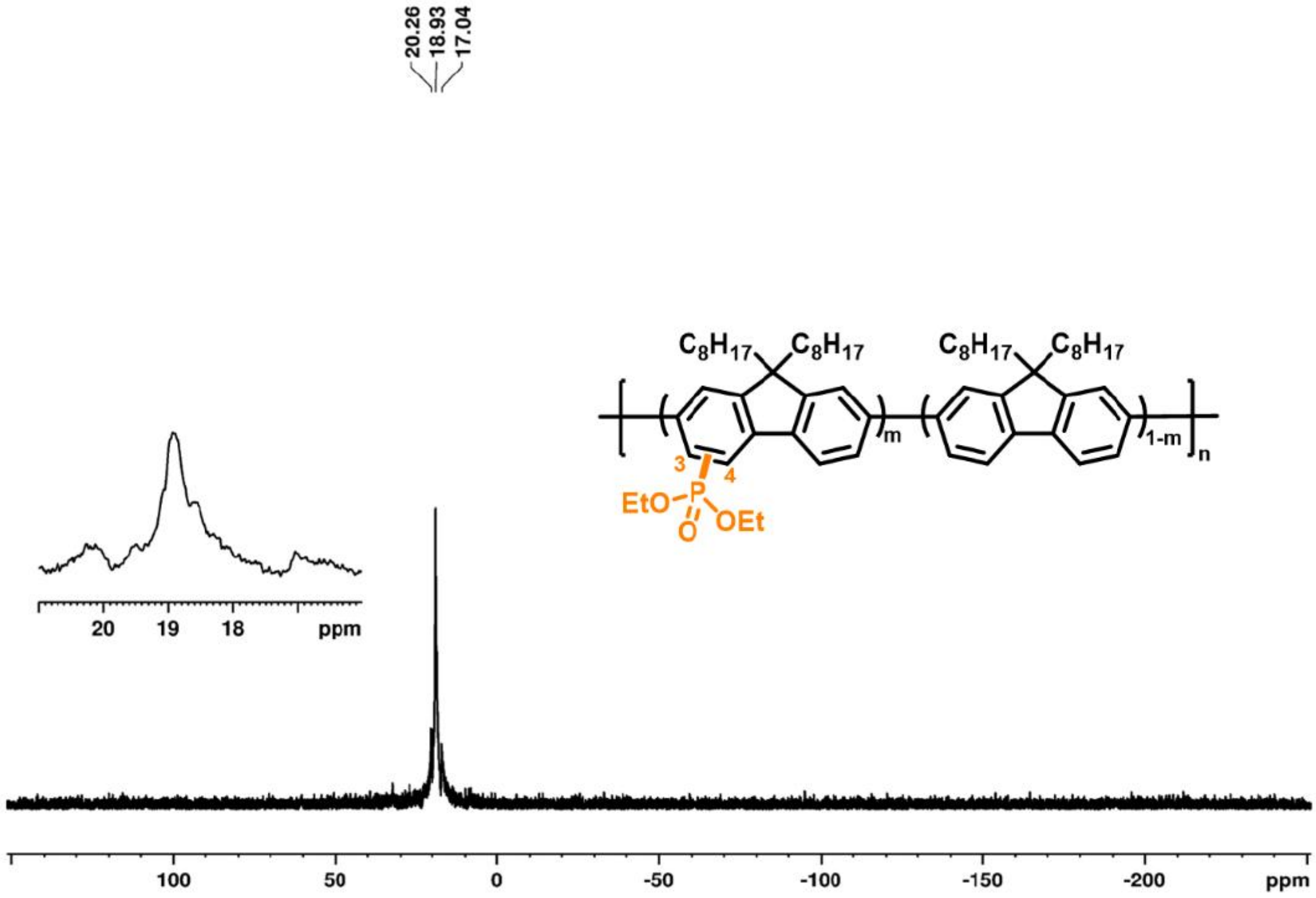

Figure S25. ^{31}P NMR spectrum (200.44 MHz, $CDCl_3$) of PFO-Phos(Et) (**P1**, DOF: 1.00).

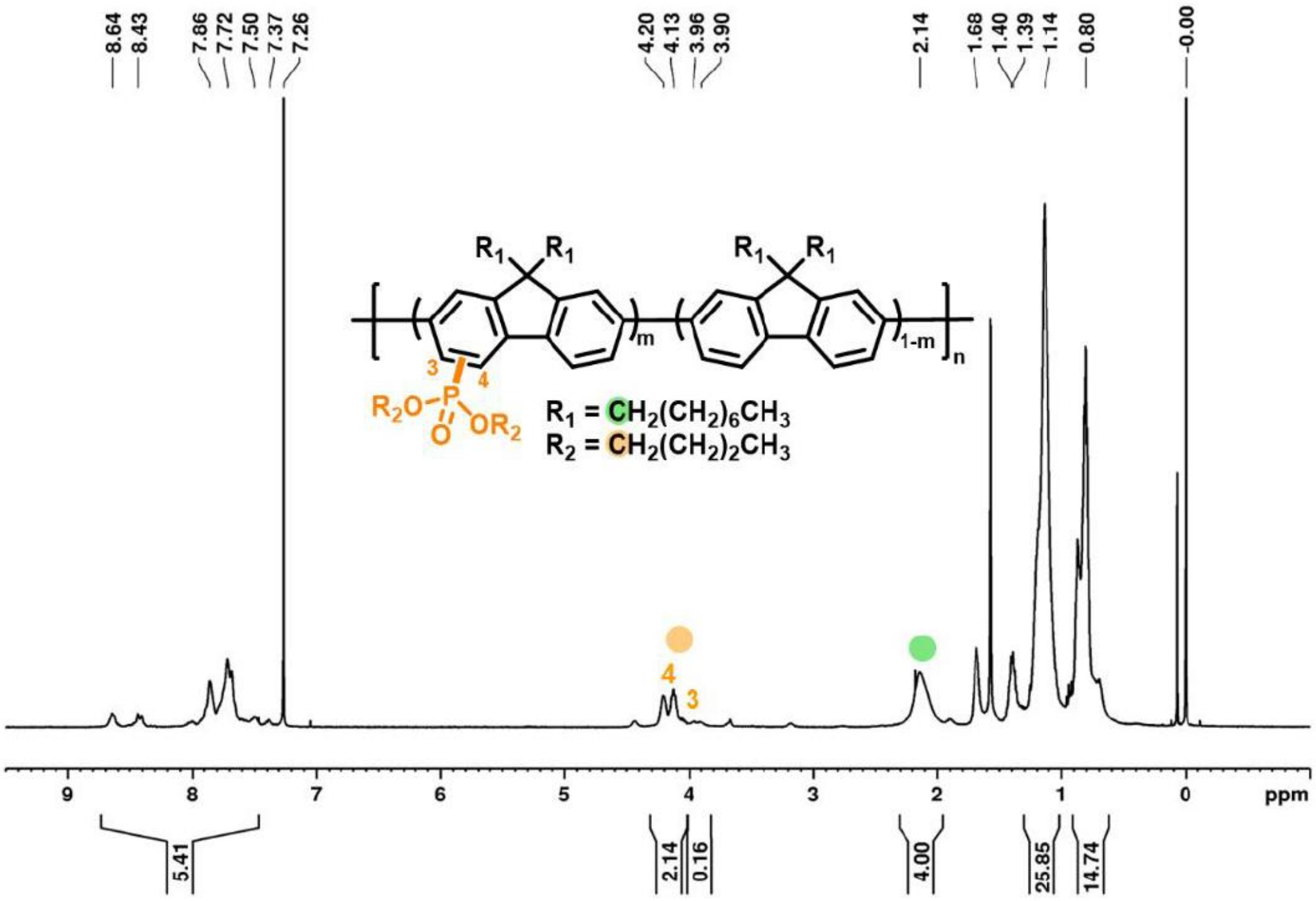

Figure S26. ^{1}H NMR spectrum (500.13 MHz, $CDCl_3$) of PFO-Phos(^{n}Bu) (**P2**, DOF: 0.81).

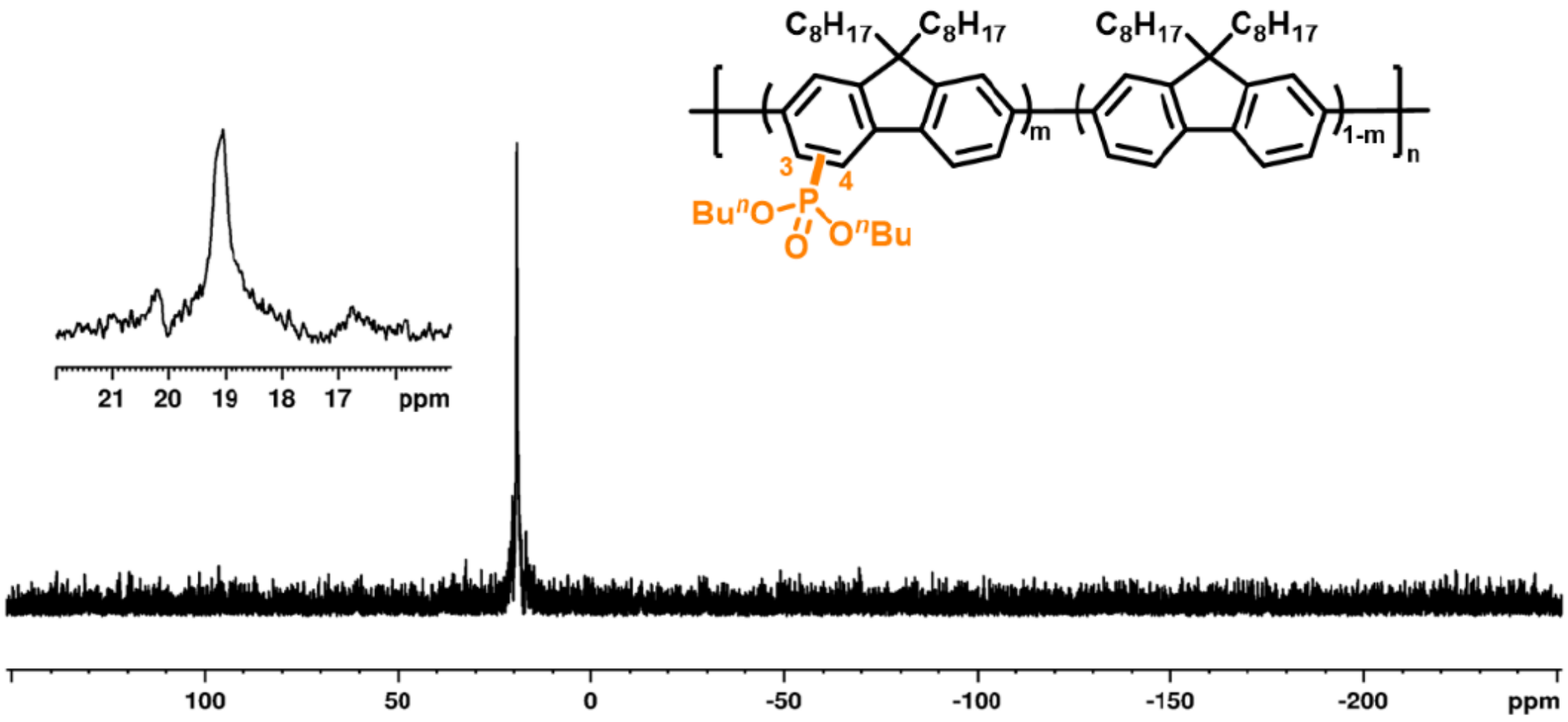

Figure S27. ^{31}P NMR spectrum (200.44 MHz, $CDCl_3$) of PFO-Phos(^{n}Bu) (**P2**, DOF: 0.81).

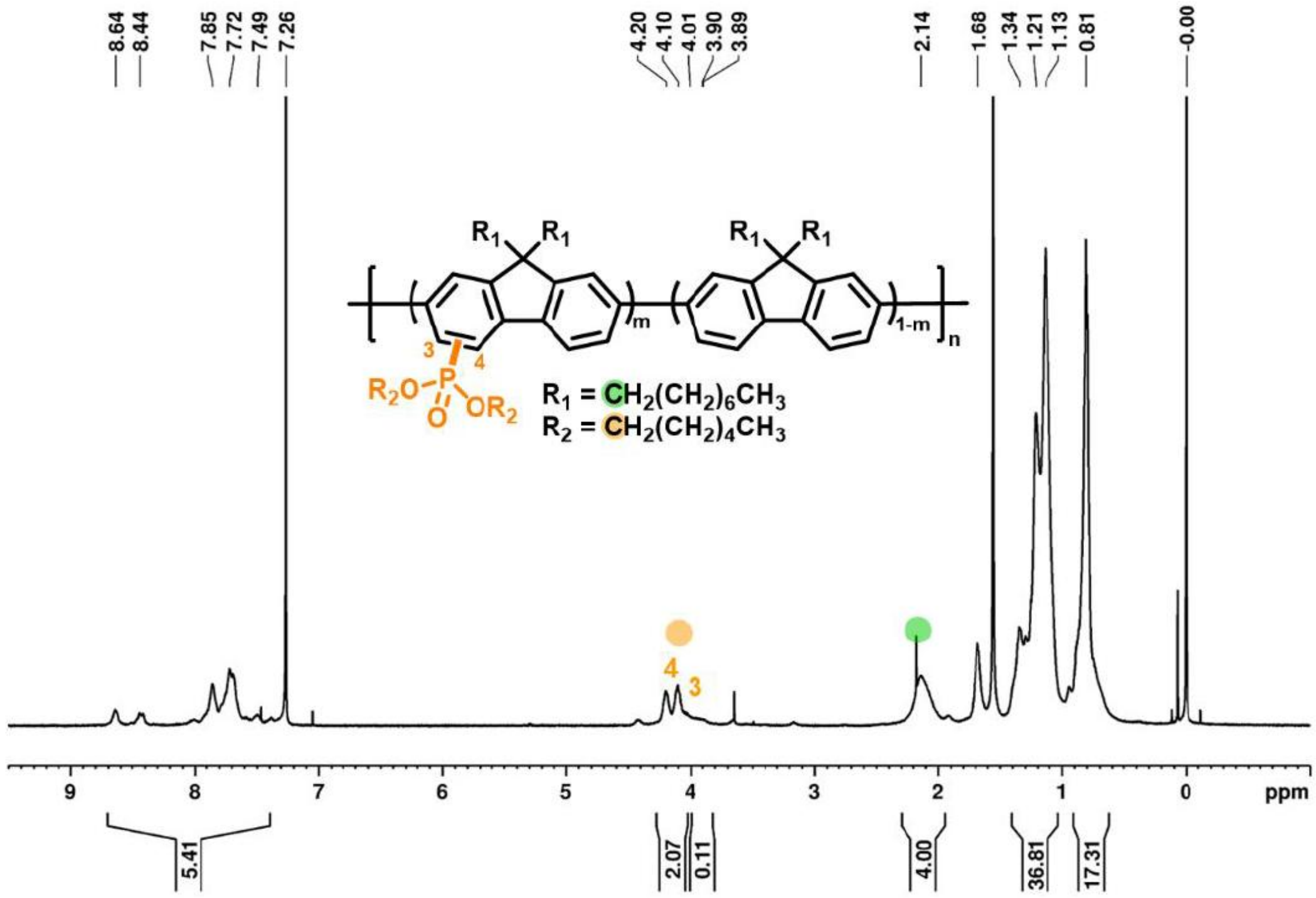

Figure S28. ^{1}H NMR spectrum (500.13 MHz, $CDCl_3$) of PFO-Phos(^{n}Hex) (**P3**, DOF: 0.74).

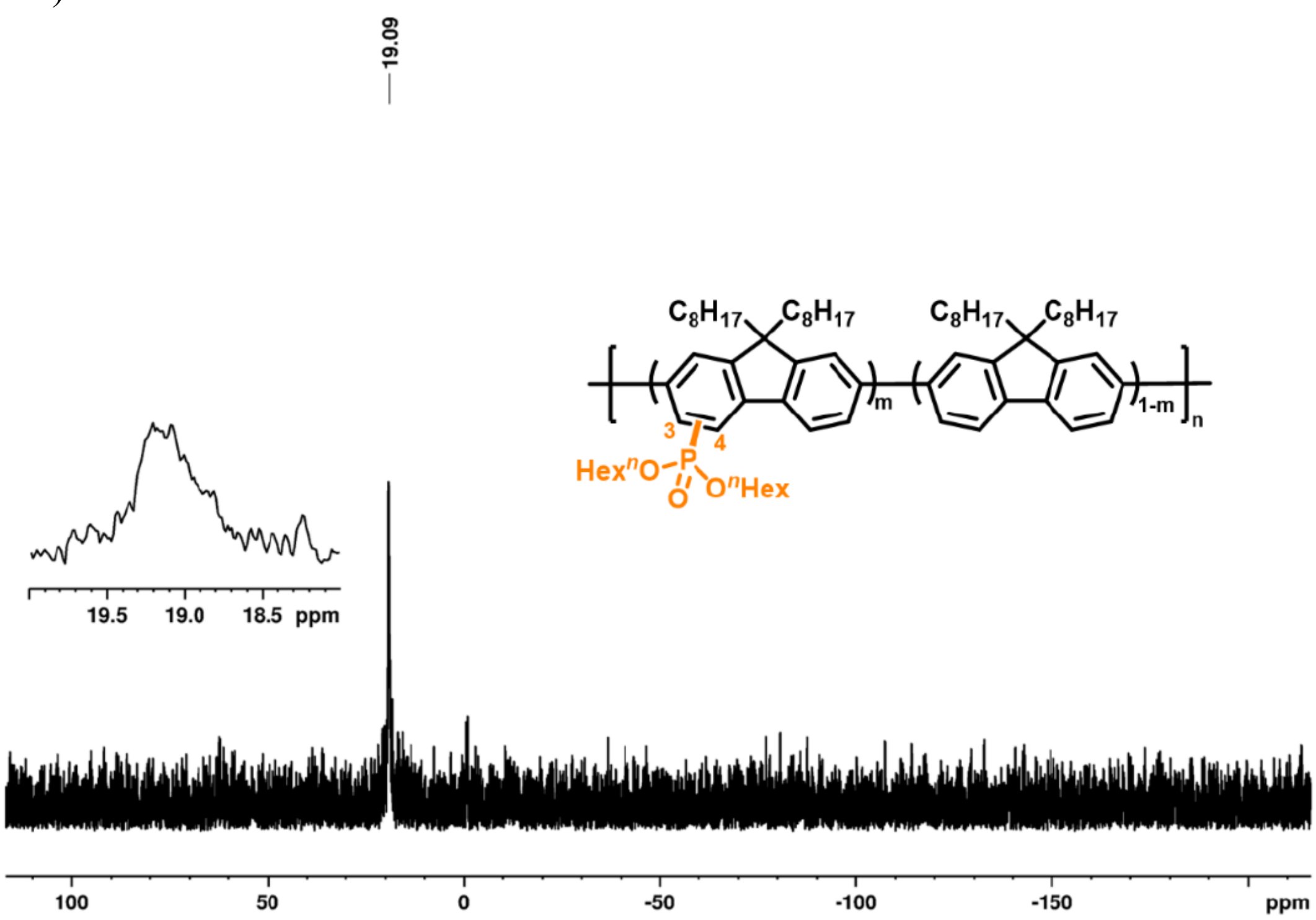

Figure S29. ^{31}P NMR spectrum (200.44 MHz, $CDCl_3$) of PFO-Phos(^{n}Hex) (**P3**, DOF: 0.74).

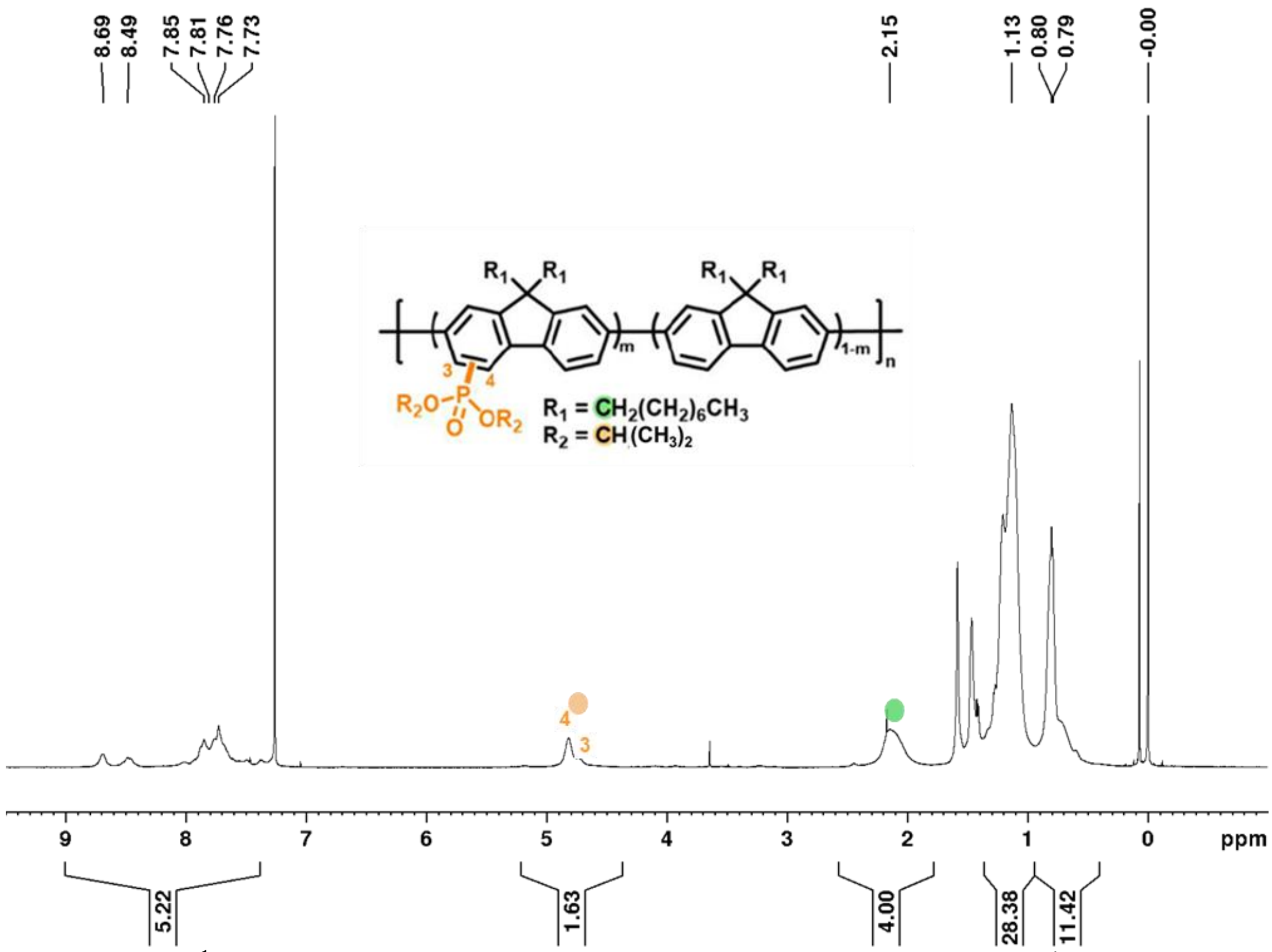

Figure S30. ^{1}H NMR spectrum (500.13 MHz, $CDCl_3$) of PFO-Phos(^{i}Pr) (**P4**, DOF: 1.00).

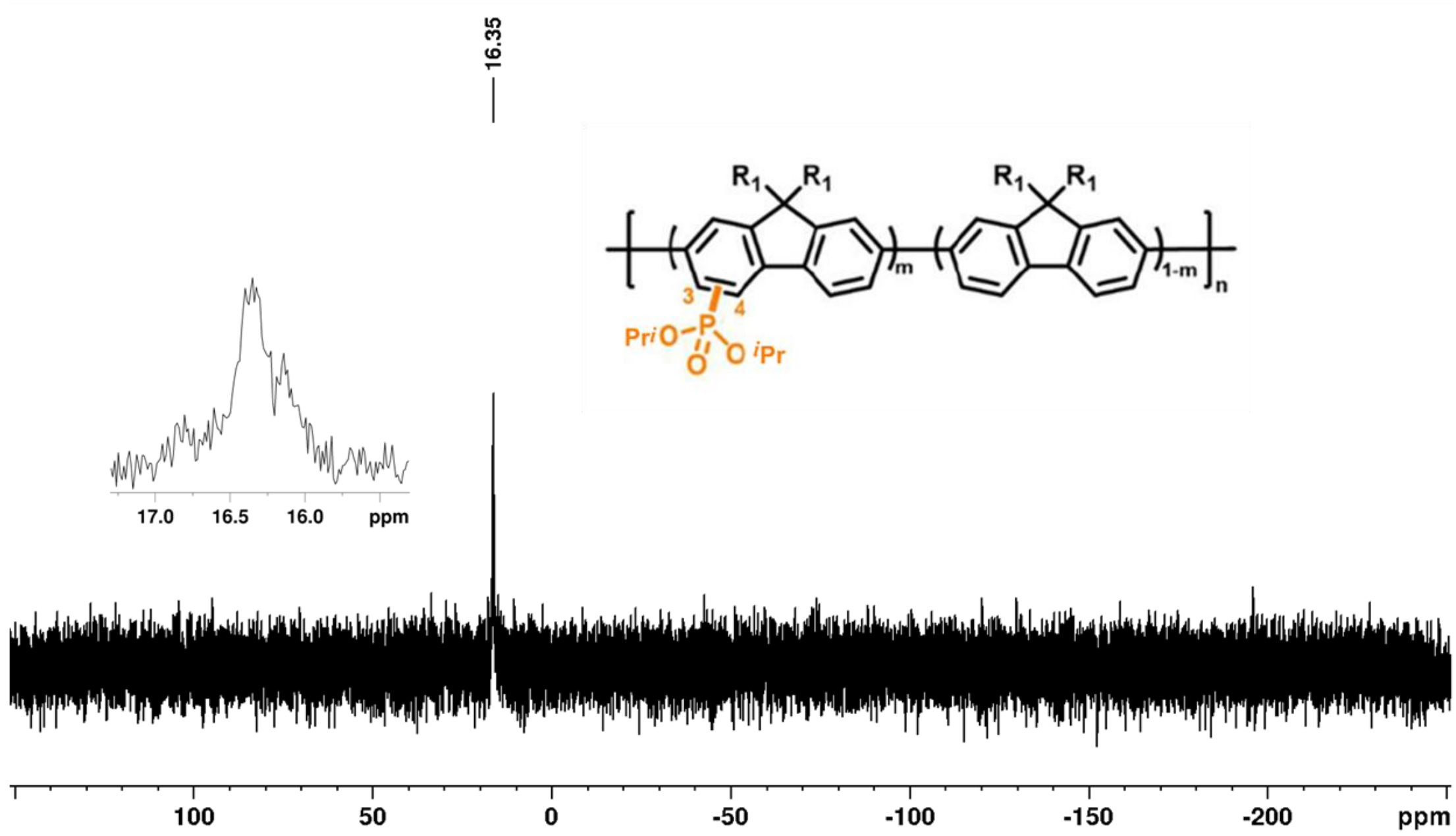

Figure S31. ^{31}P NMR spectrum (200.44 MHz, $CDCl_3$) of PFO-Phos(^{i}Pr) (**P4**, DOF: 1.00).

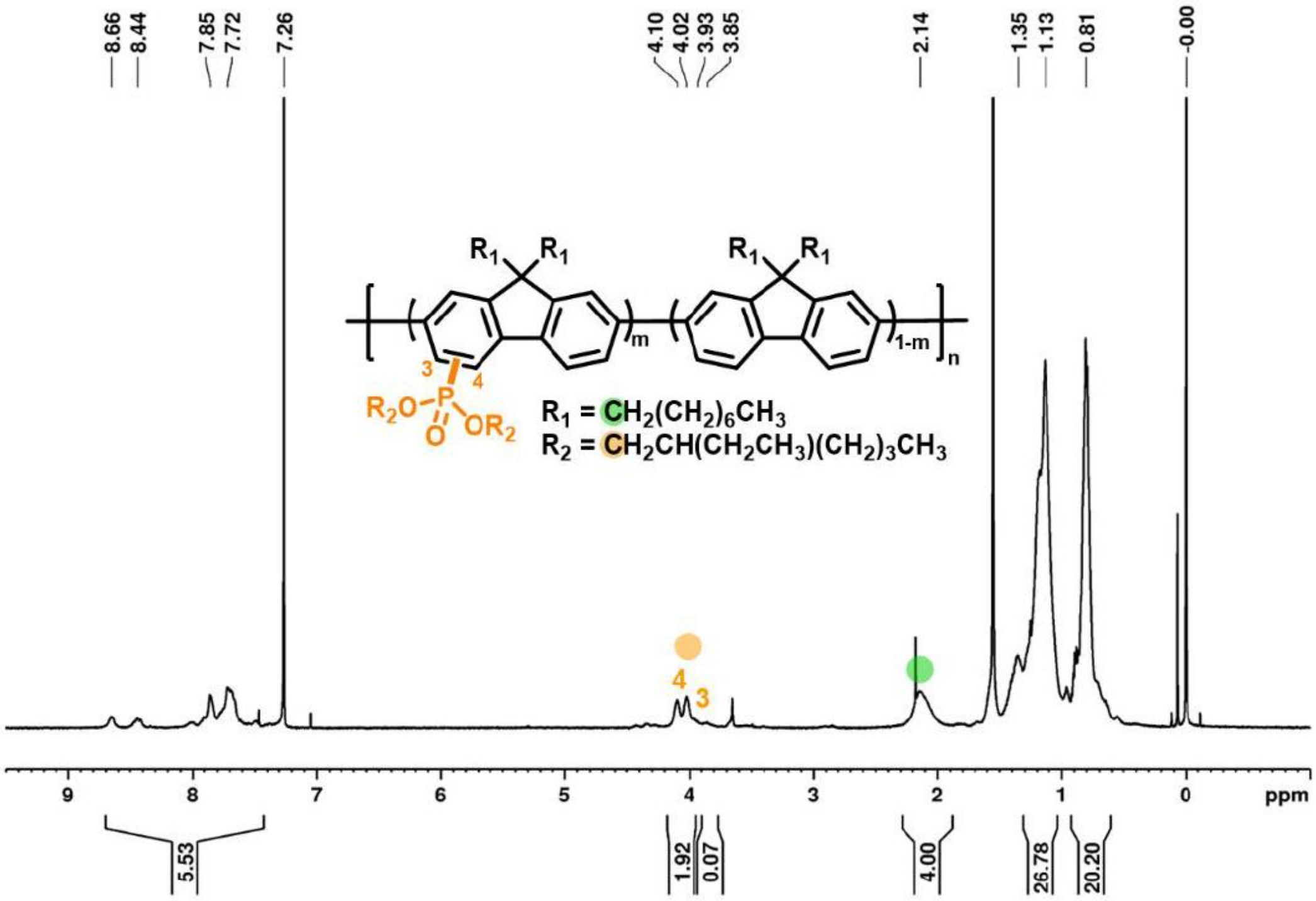

Figure S32. ^{1}H NMR spectrum (500.13 MHz, $CDCl_3$) of PFO-Phos(EH) (**P5**, DOF: 0.69). EH represents 2-ethyl-hexyl group.

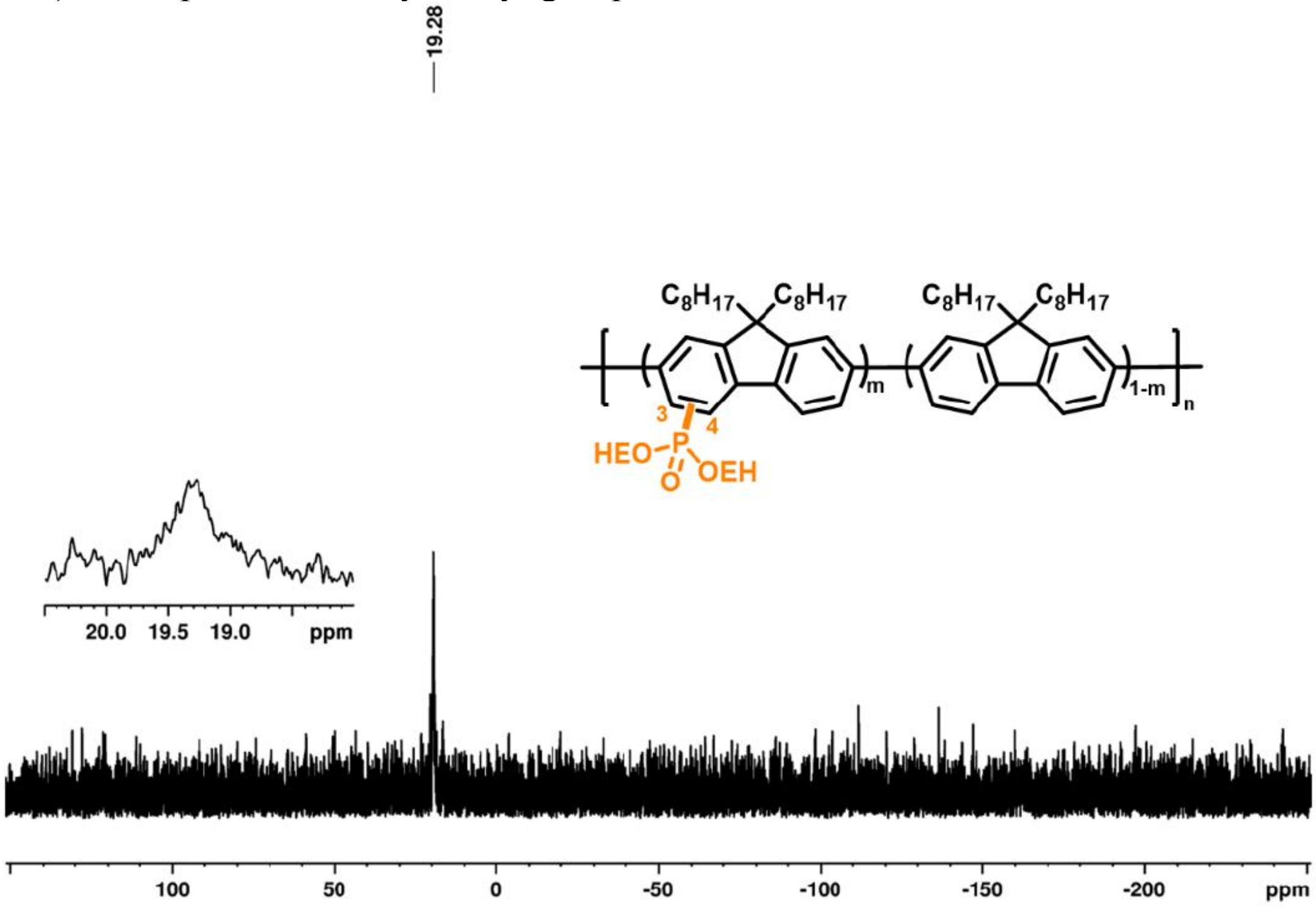

Figure S33. ^{31}P NMR spectrum (200.44 MHz, $CDCl_3$) of PFO-Phos(EH) (**P5**, DOF: 0.69). EH represents 2-ethyl-hexyl group.

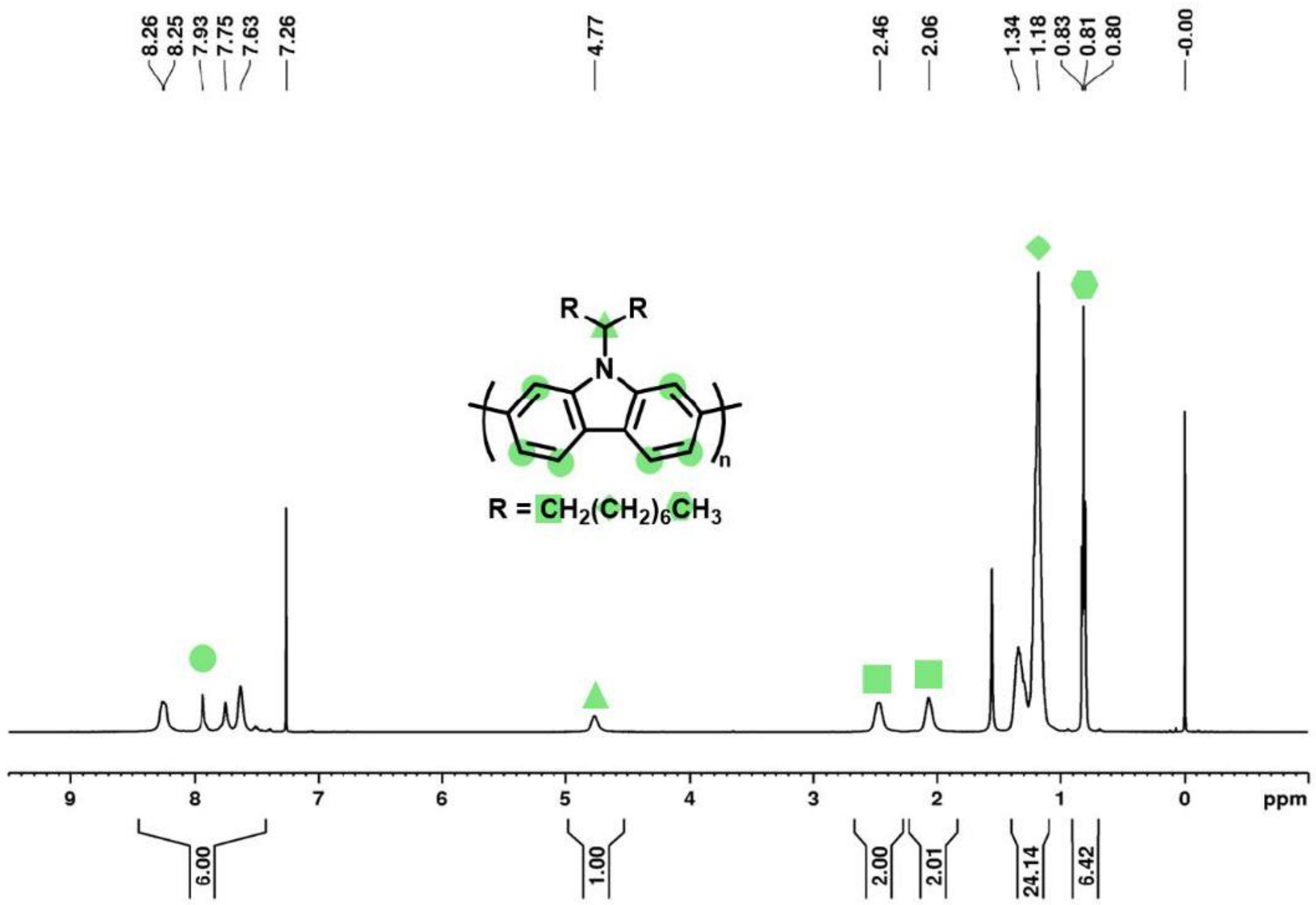

Figure S34. ^{1}H NMR spectrum (500.13 MHz, $CDCl_3$) of PCD (**Pre6**).

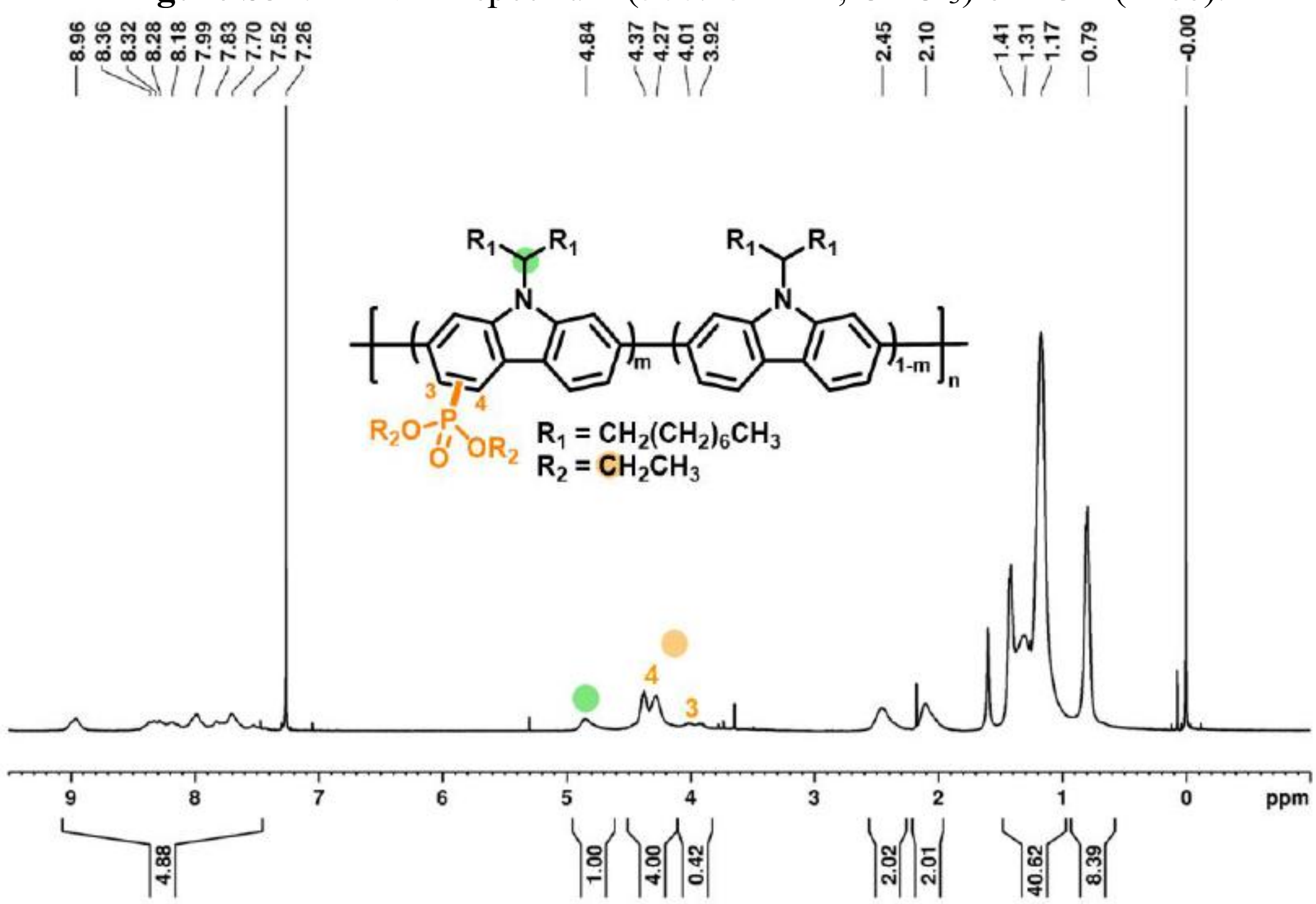

Figure S35. ^{1}H NMR spectrum (500.13 MHz, $CDCl_3$) of PCD-Phos(Et) (**P6**, DOF: 1.25).

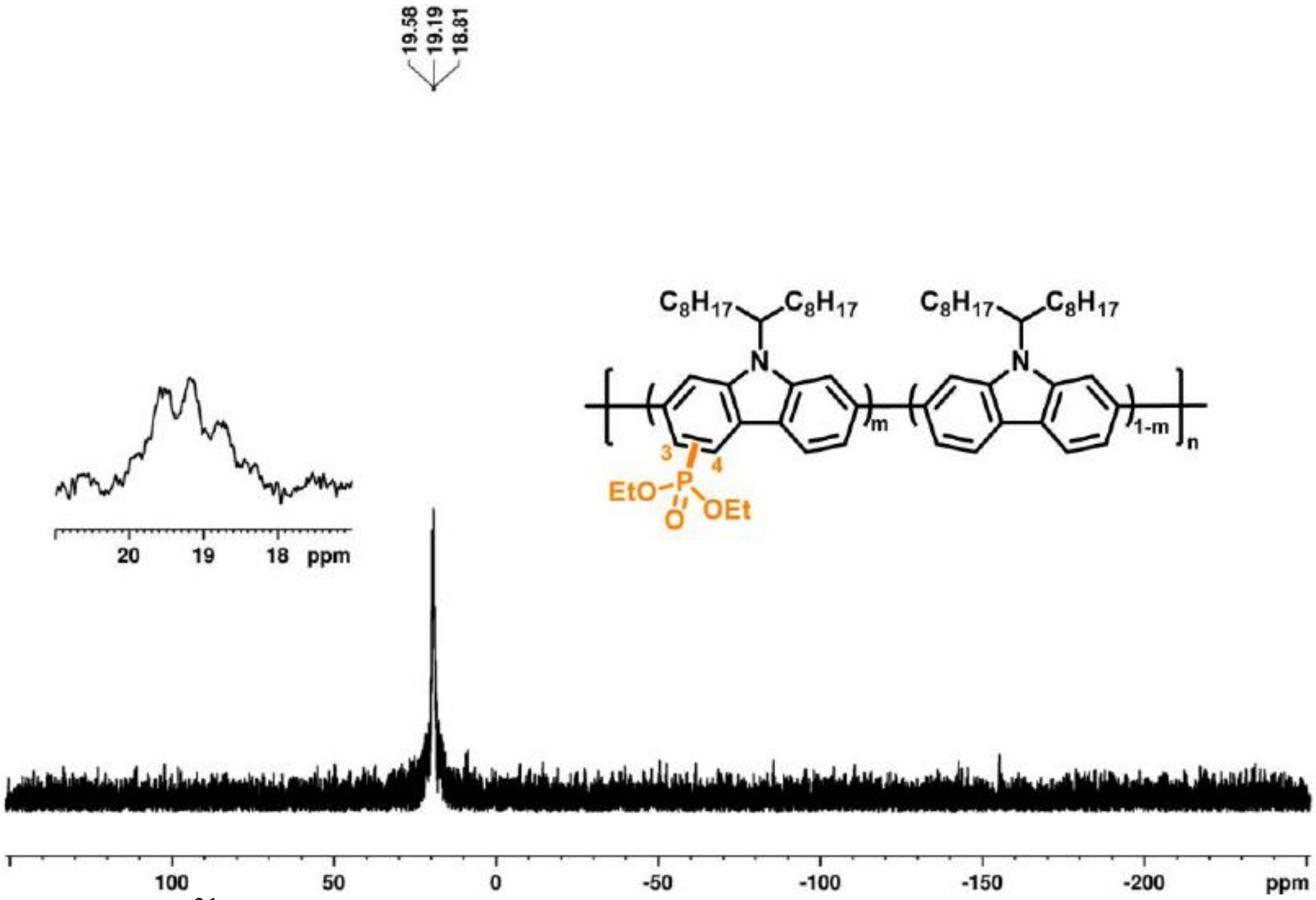

Figure S36. ^{31}P NMR spectrum (200.44 MHz, $CDCl_3$) of PCD-Phos(Et) (**P6**, DOF: 1.25).

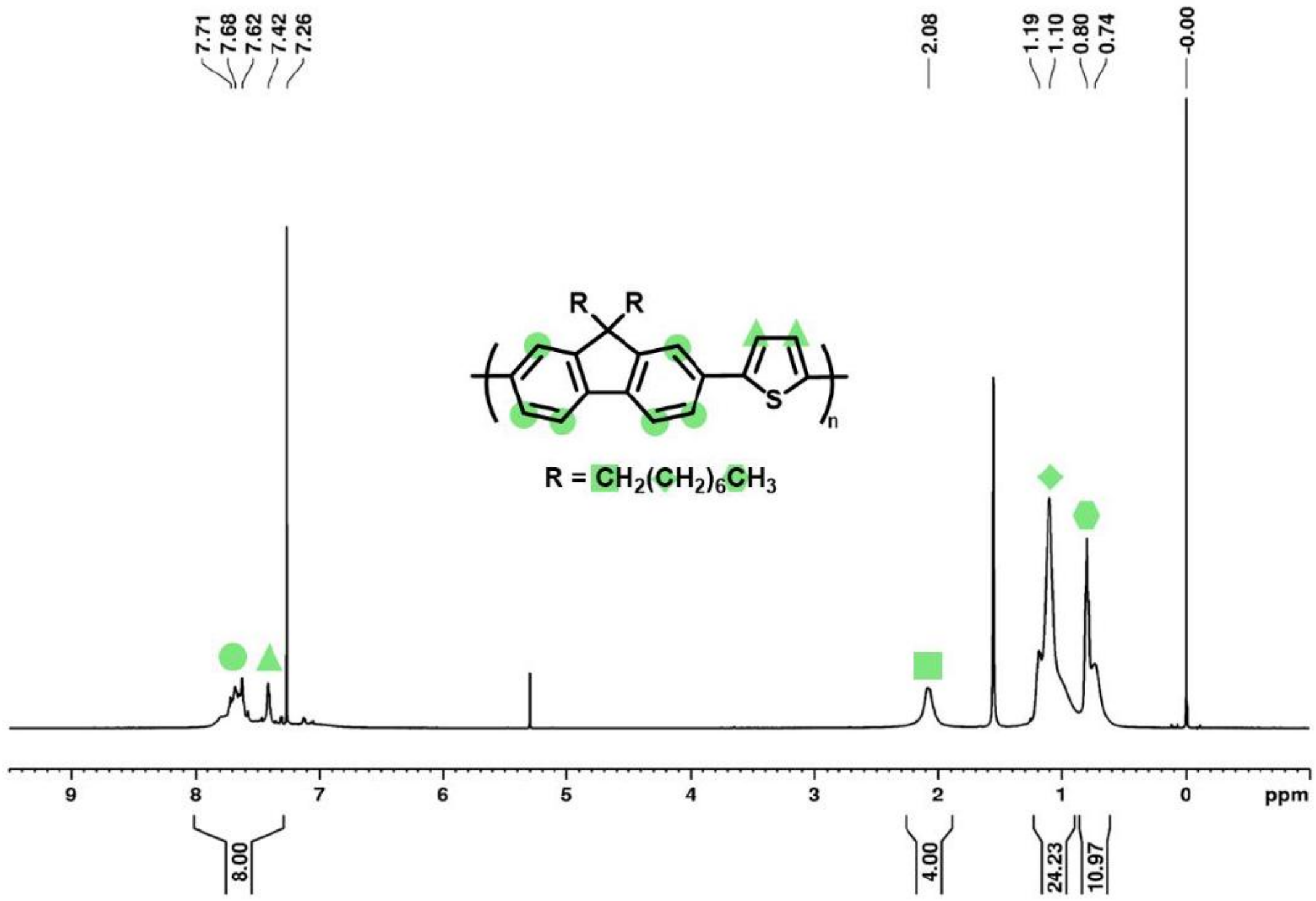

Figure S37. ^{1}H NMR spectrum (500.13 MHz, $CDCl_3$) of P(Fl-Th) (**Pre7**).

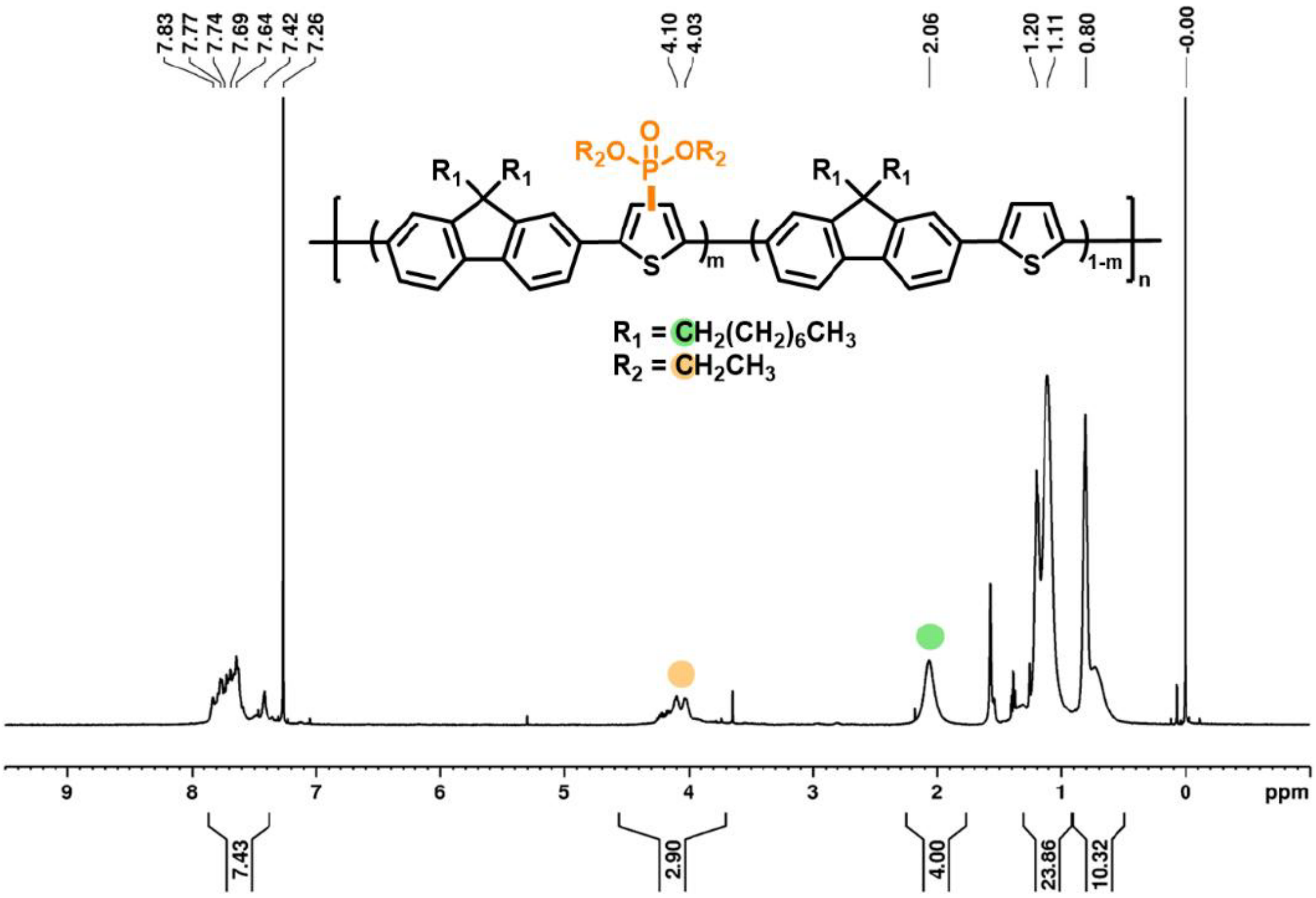

Figure S38. ^{1}H NMR spectrum (500.13 MHz, $CDCl_3$) of P(Fl-Th-Phos(Et)) (**P7**, 3 h) (DOF: 0.78).

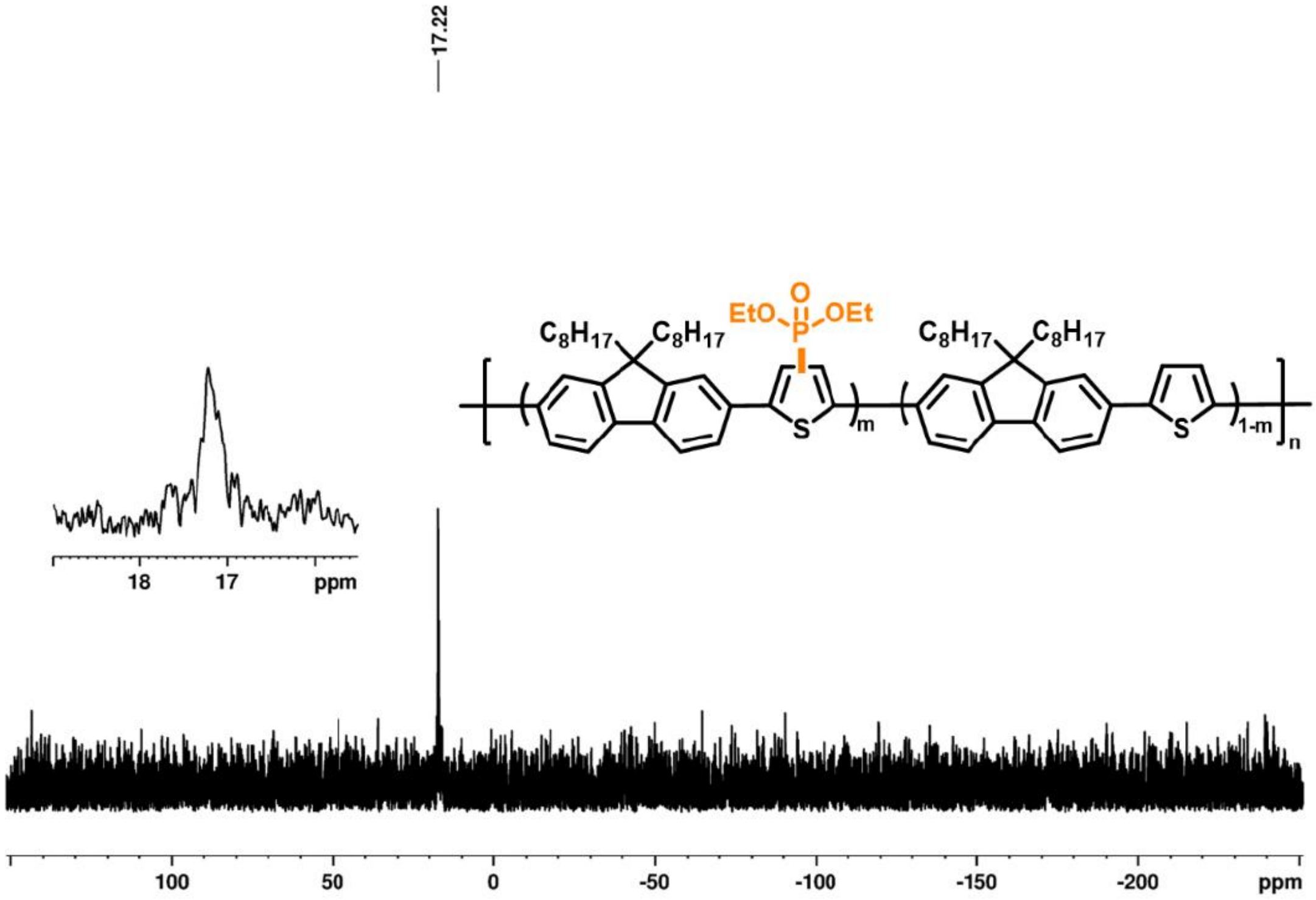

Figure S39. ^{31}P NMR spectrum (200.44 MHz, $CDCl_3$) of P(Fl-Th-Phos(Et)) (**P7**, 3 h) (DOF: 0.78).

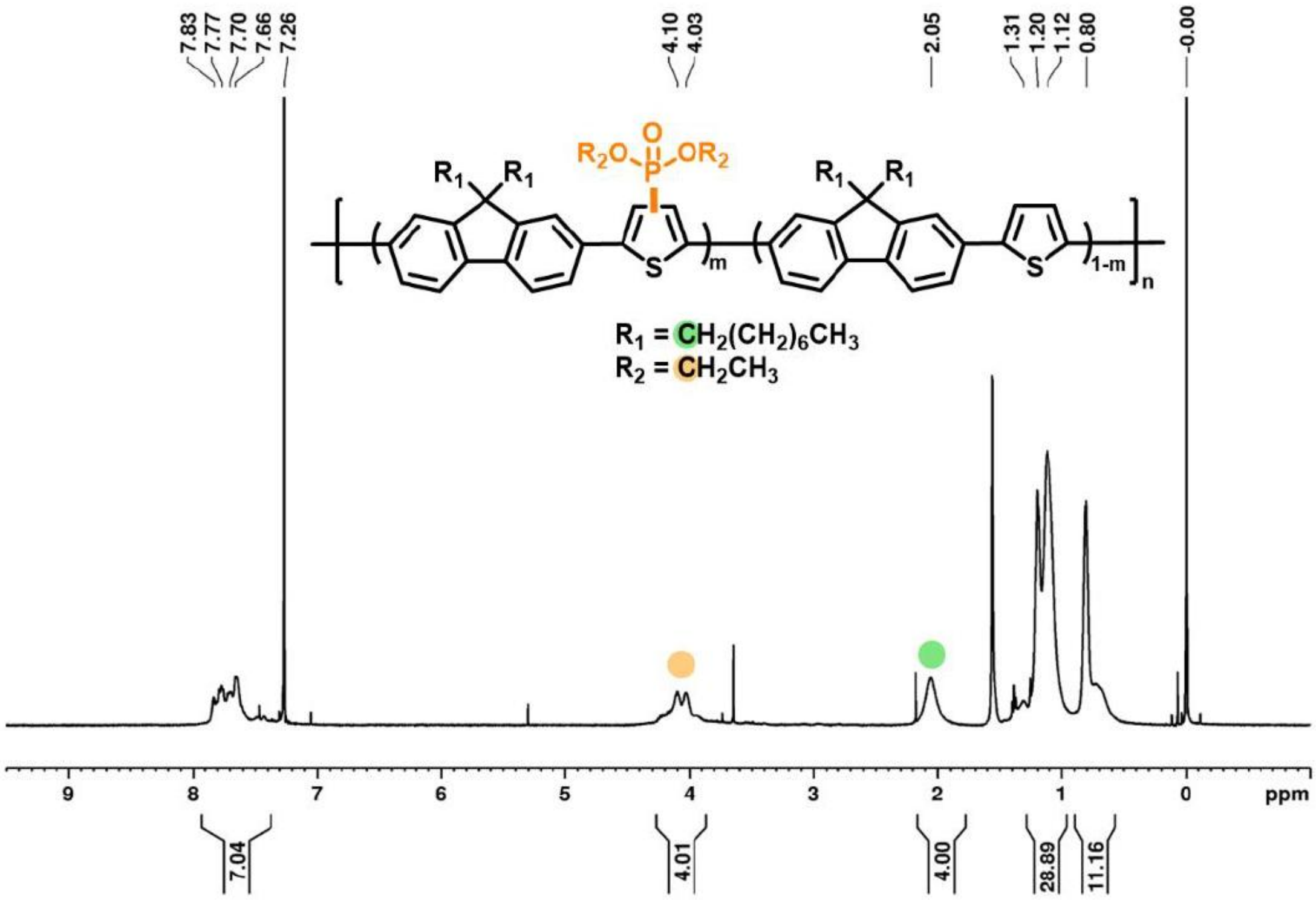

Figure S40. ^{1}H NMR spectrum (500.13 MHz, $CDCl_3$) of P(Fl-Th-Phos(Et)) (**P7**, 5 h) (DOF: 1.00).

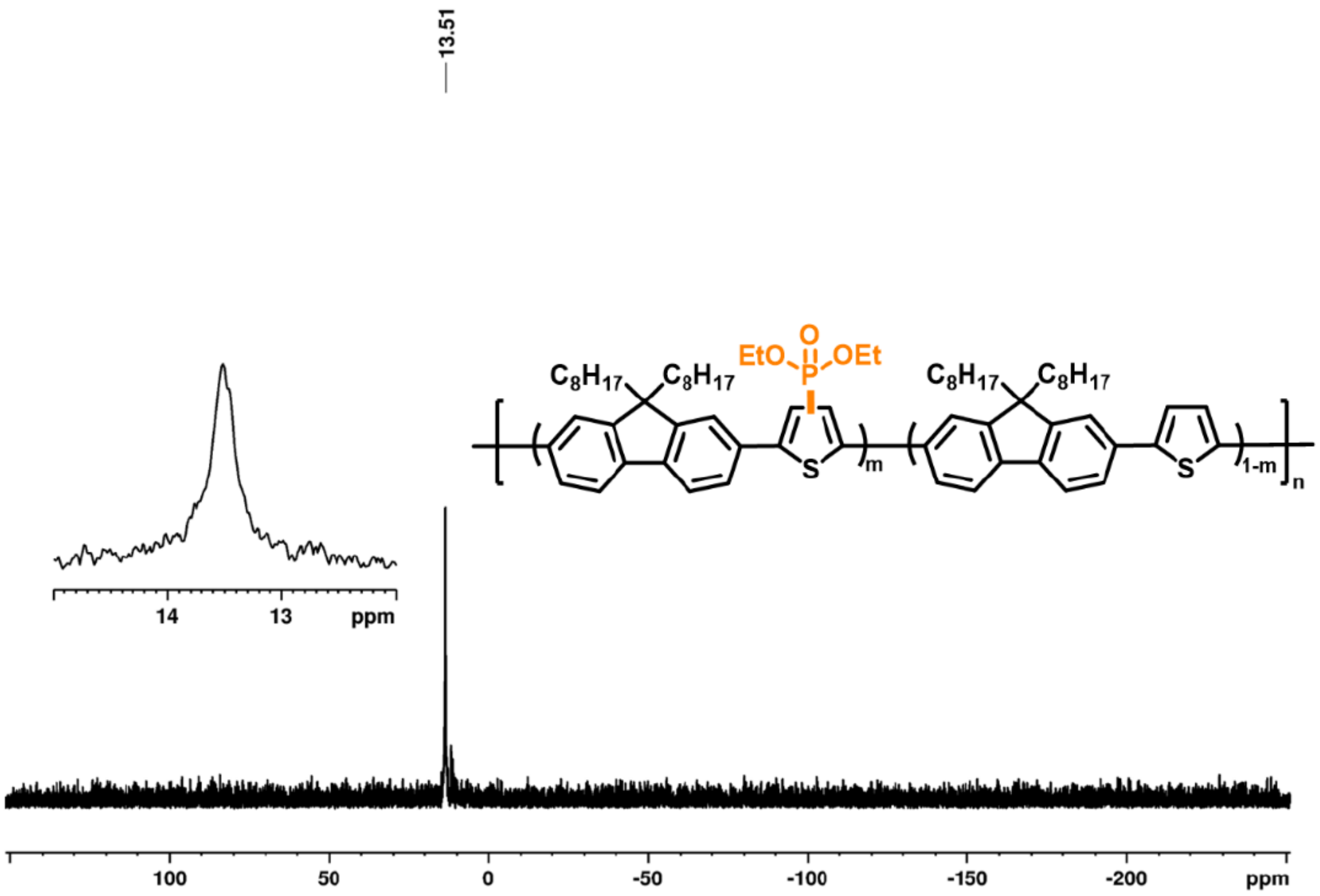

Figure S41. ^{31}P NMR spectrum (200.44 MHz, $CDCl_3$) of P(Fl-Th-Phos(Et)) (**P7**, 5 h) (DOF: 1.00).

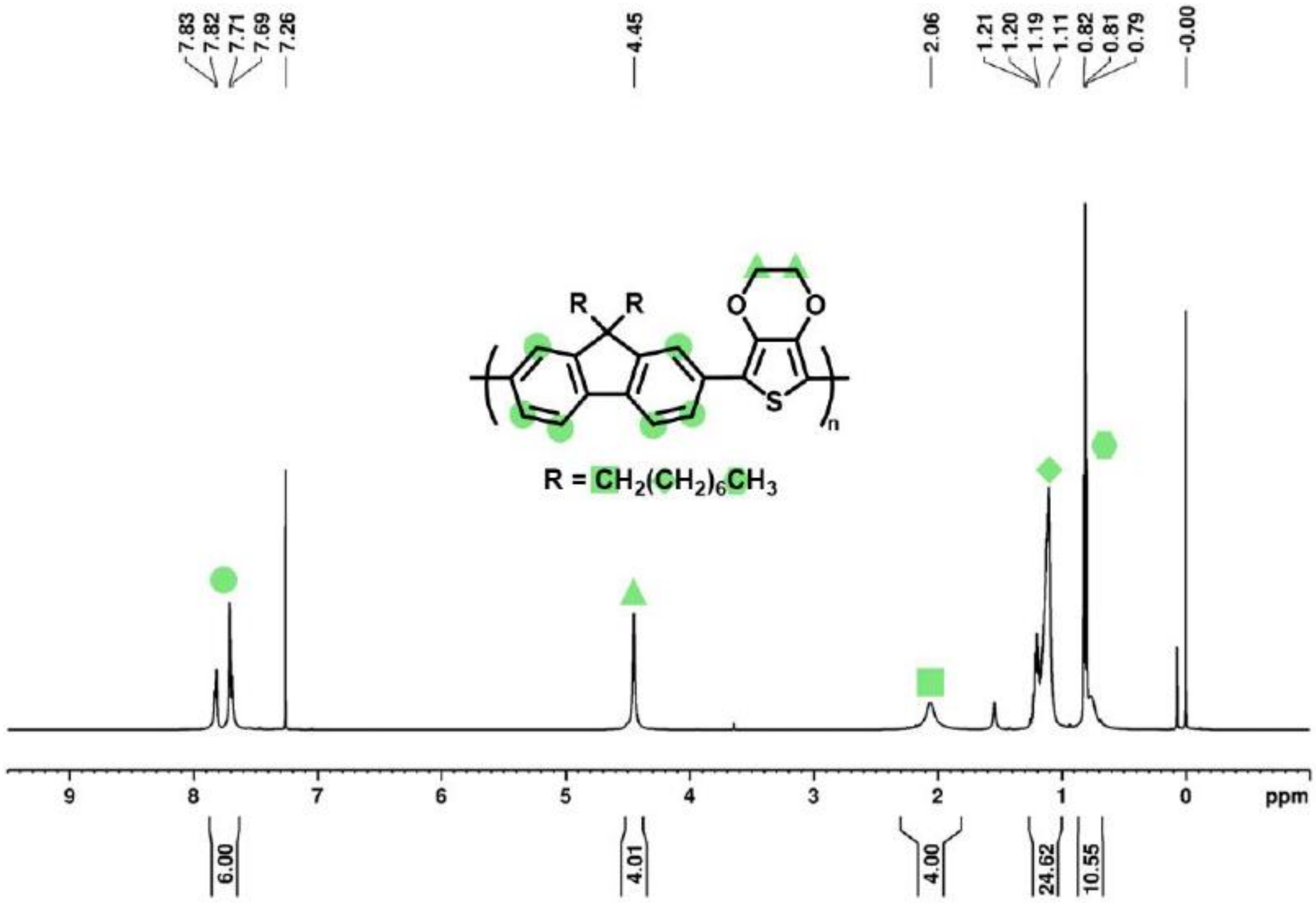

Figure S42. ^{1}H NMR spectrum (500.13 MHz, $CDCl_3$) of P(Fl-EDOT) (**Pre8**).

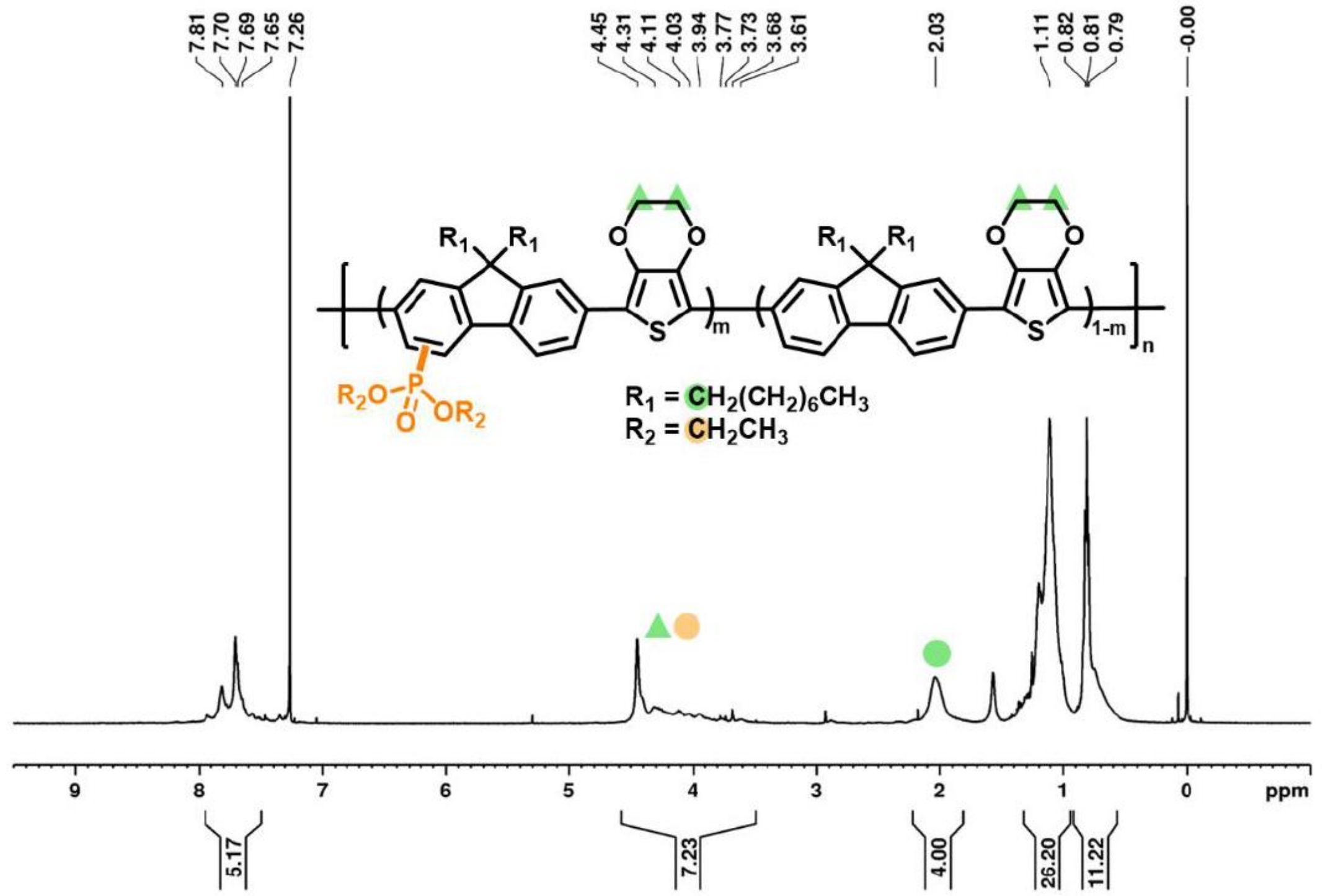

Figure S43. ^{1}H NMR spectrum (500.13 MHz, $CDCl_3$) of P(Fl-EDOT-Phos(Et)) (3 h) (**P8**, DOF : 0.71).

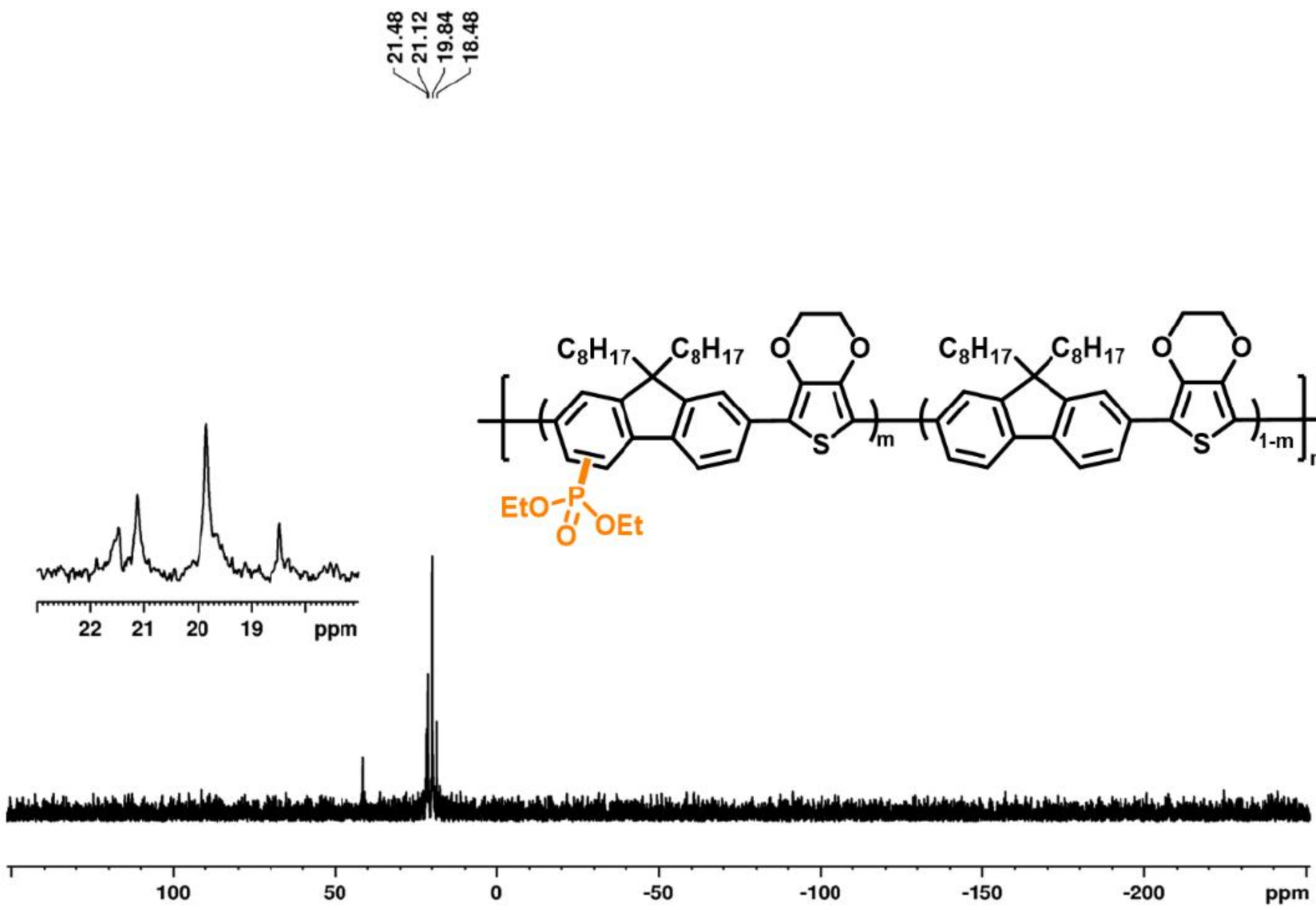

Figure S44. ^{31}P NMR spectrum (200.44 MHz, $CDCl_3$) of P(Fl-EDOT-Phos(Et)) (3 h) (**P8**, DOF: 0.71).

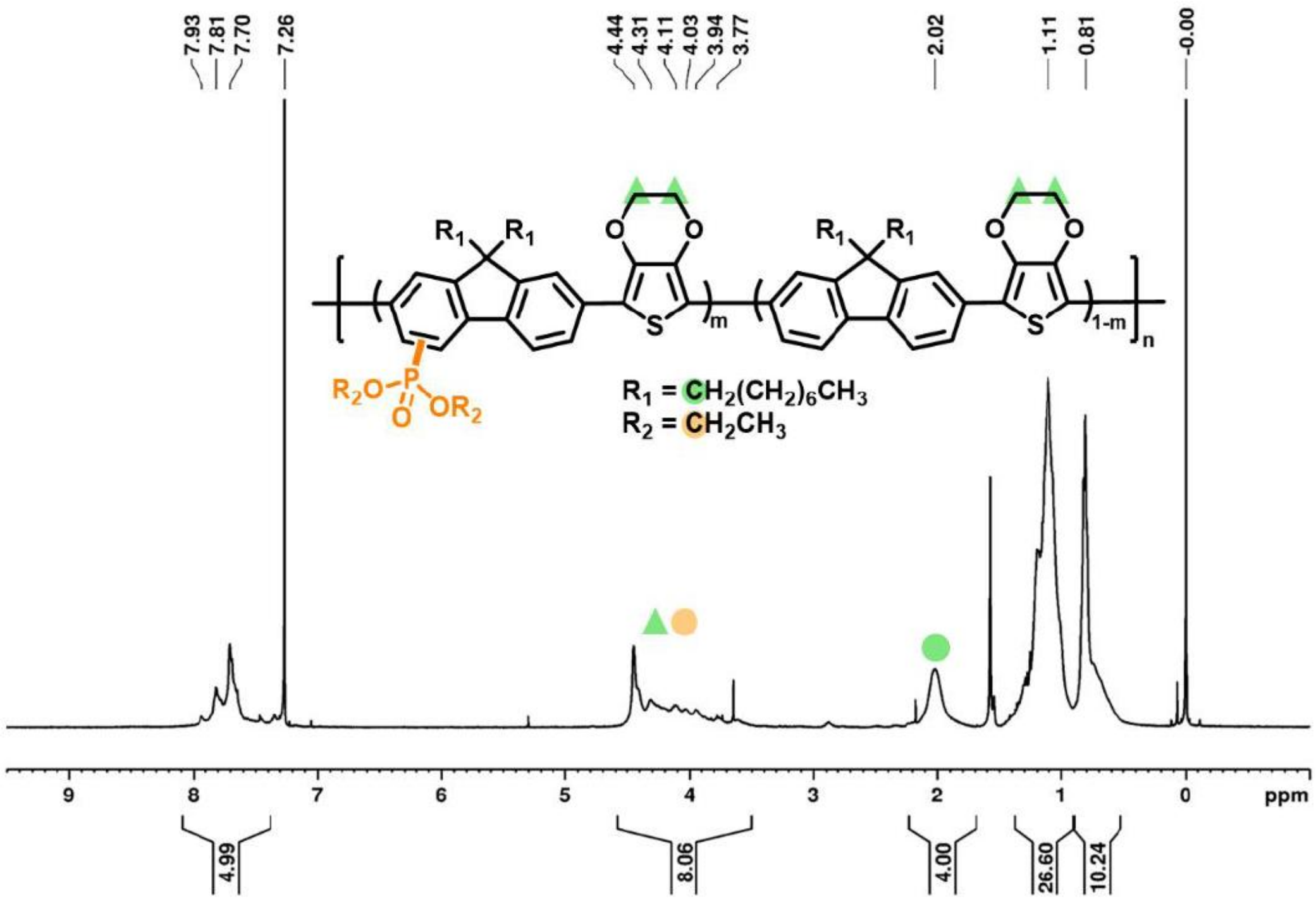

Figure S45. ^{1}H NMR spectrum (500.13 MHz, $CDCl_3$) of P(Fl-EDOT-Phos(Et)) (5 h) (**P8**, DOF : 1.00).

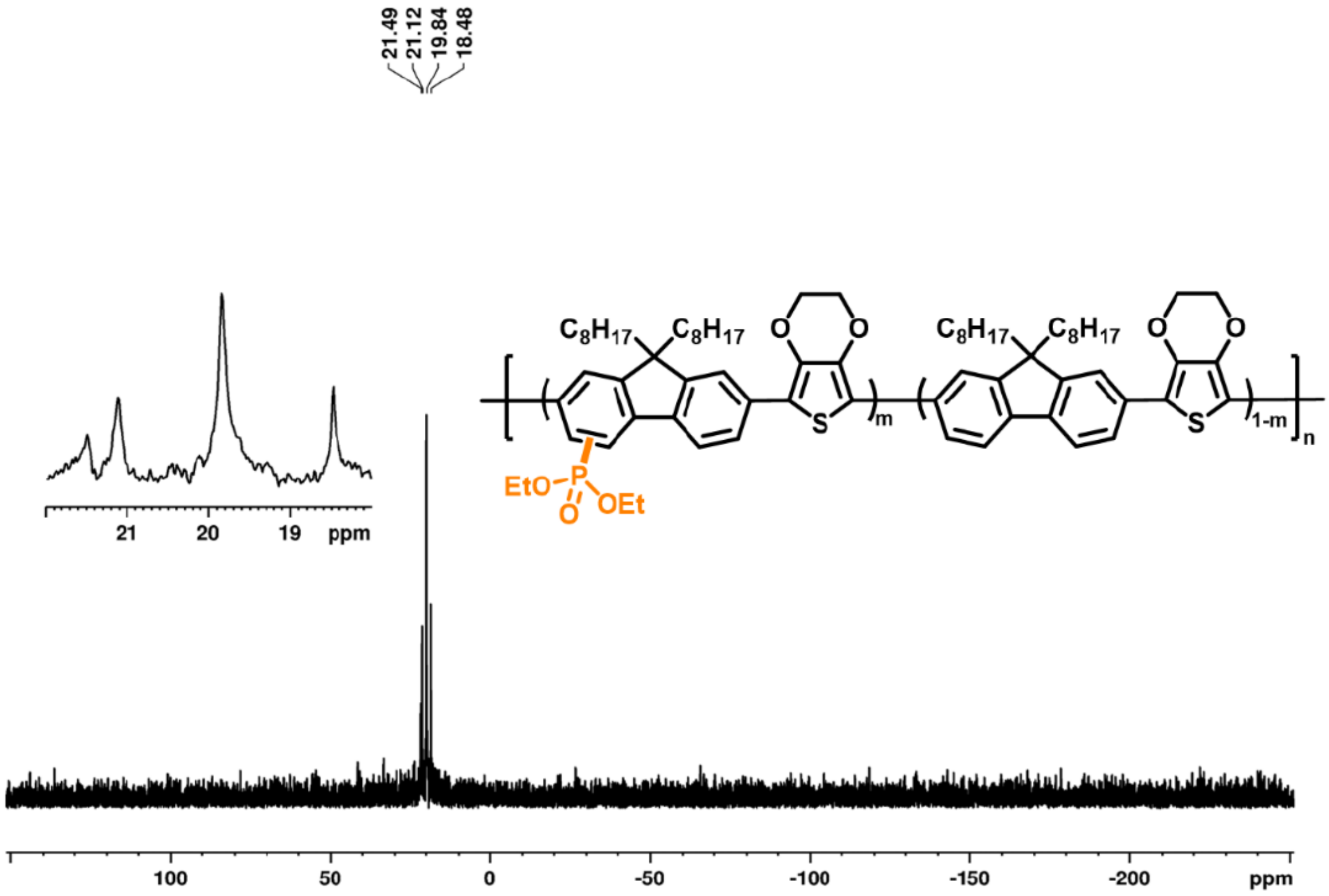

Figure S46. ^{31}P NMR spectrum (200.44 MHz, $CDCl_3$) of P(Fl-EDOT-Phos(Et)) (5 h) (**P8**, DOF: 1.00).

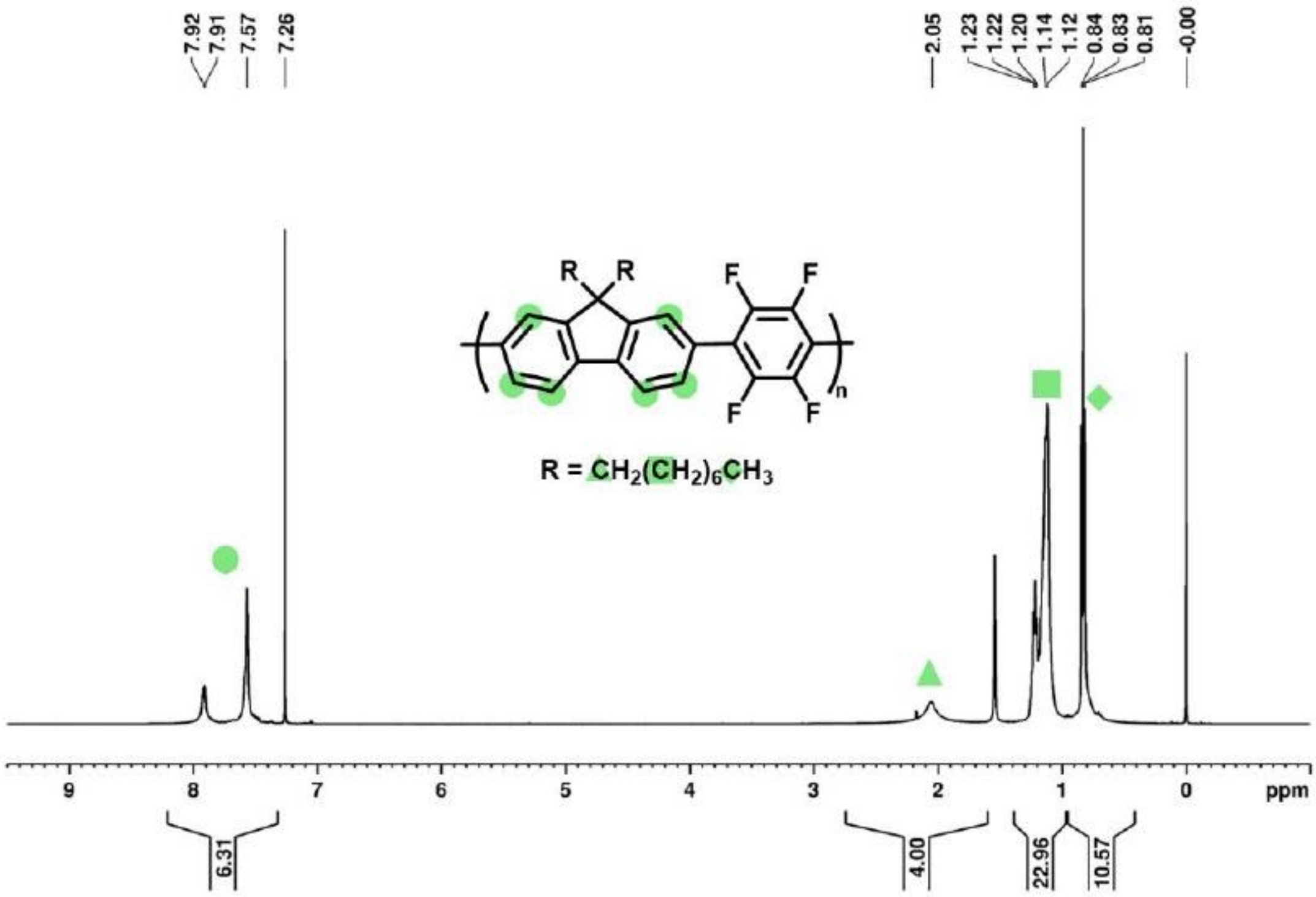

Figure S47. ^{1}H NMR spectrum (500.13 MHz, $CDCl_3$) of P(Fl-TFB) (**Pre9**).

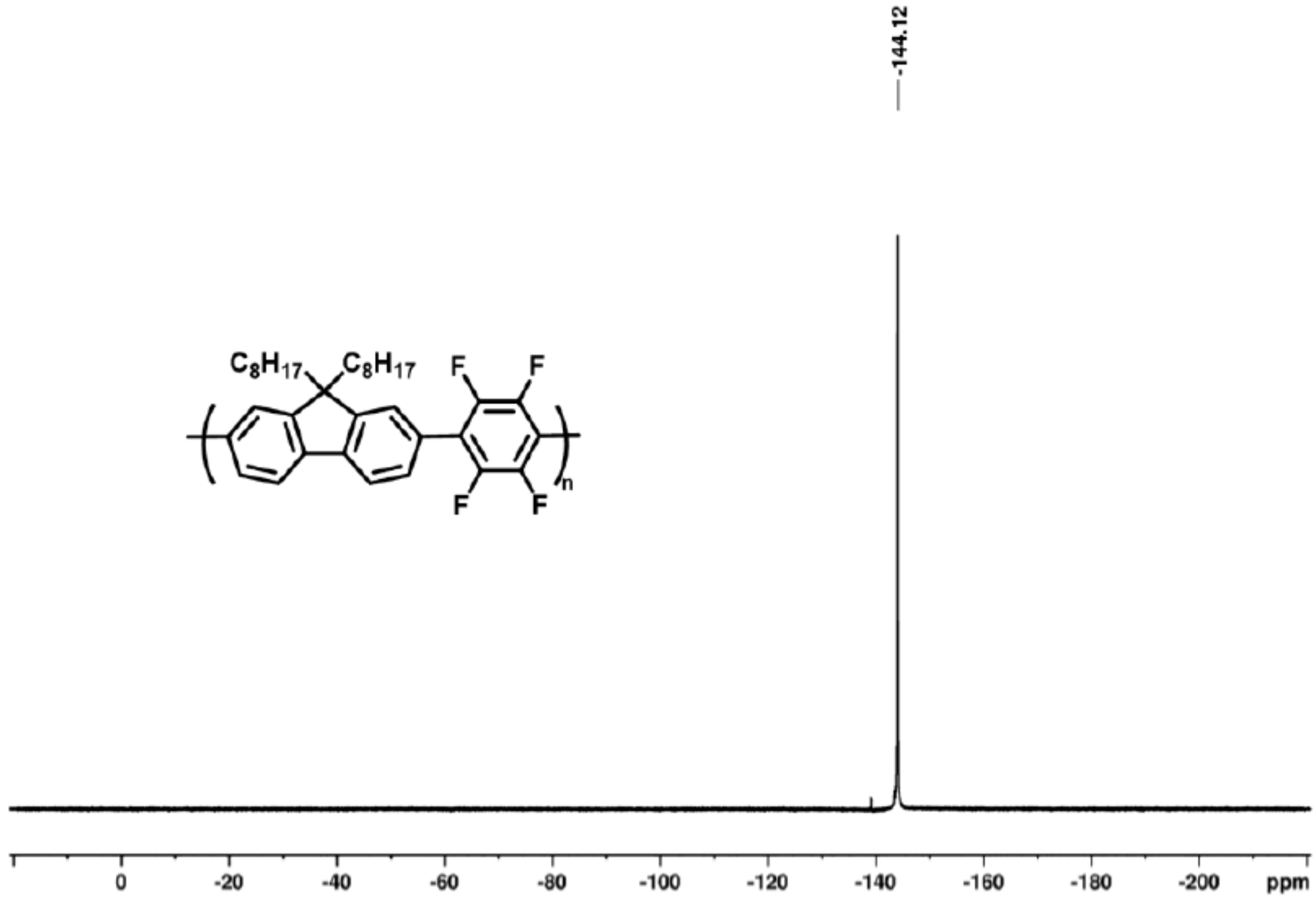

Figure S48. ^{19}F NMR spectrum (470.59 MHz, $CDCl_3$) of P(Fl-TFB) (**Pre9**).

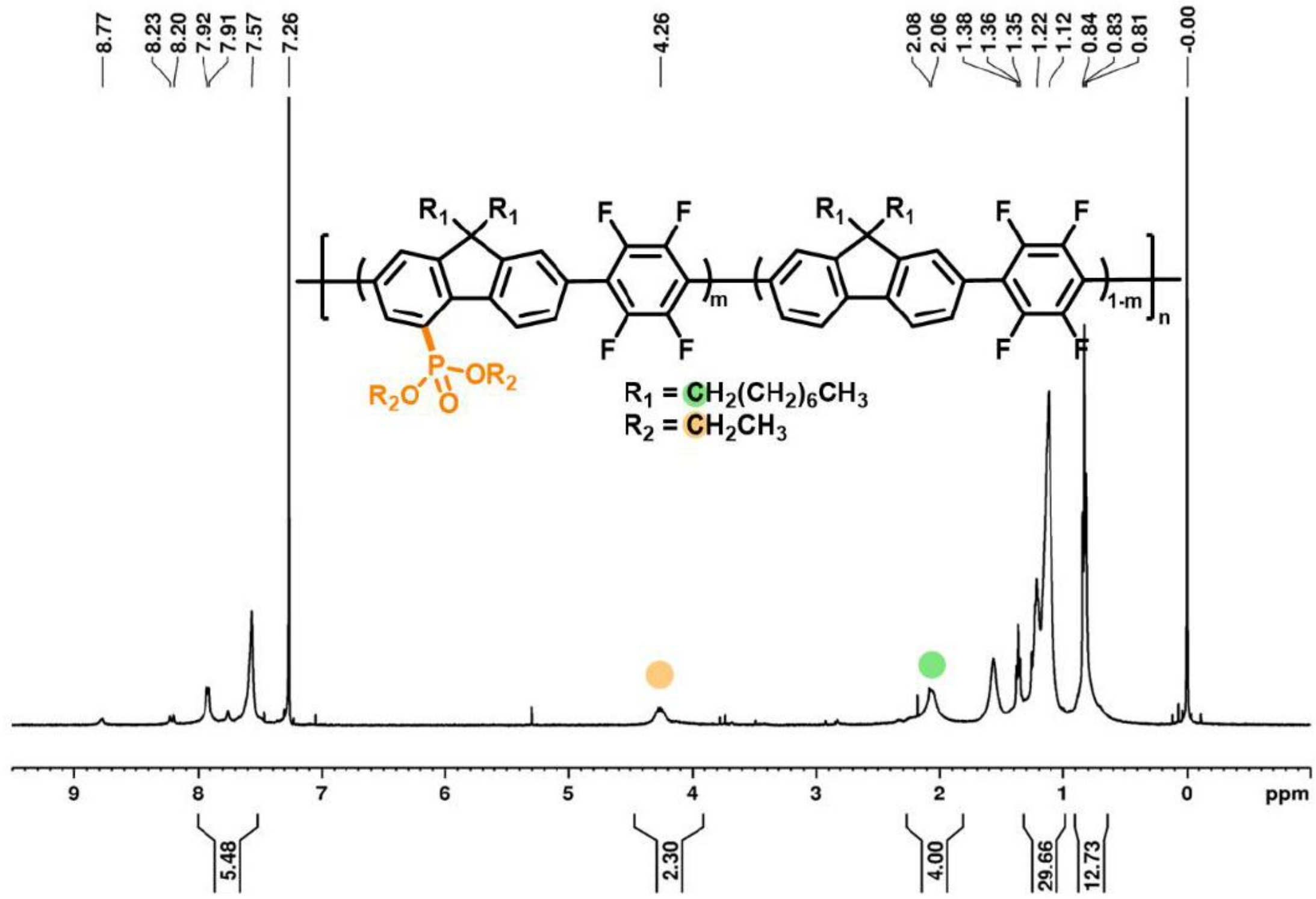

Figure S49. ^{1}H NMR spectrum (500.13 MHz, $CDCl_3$) of P(Fl-TFB-Phos(Et)) (3 h) (**P9**, DOF : 0.44).

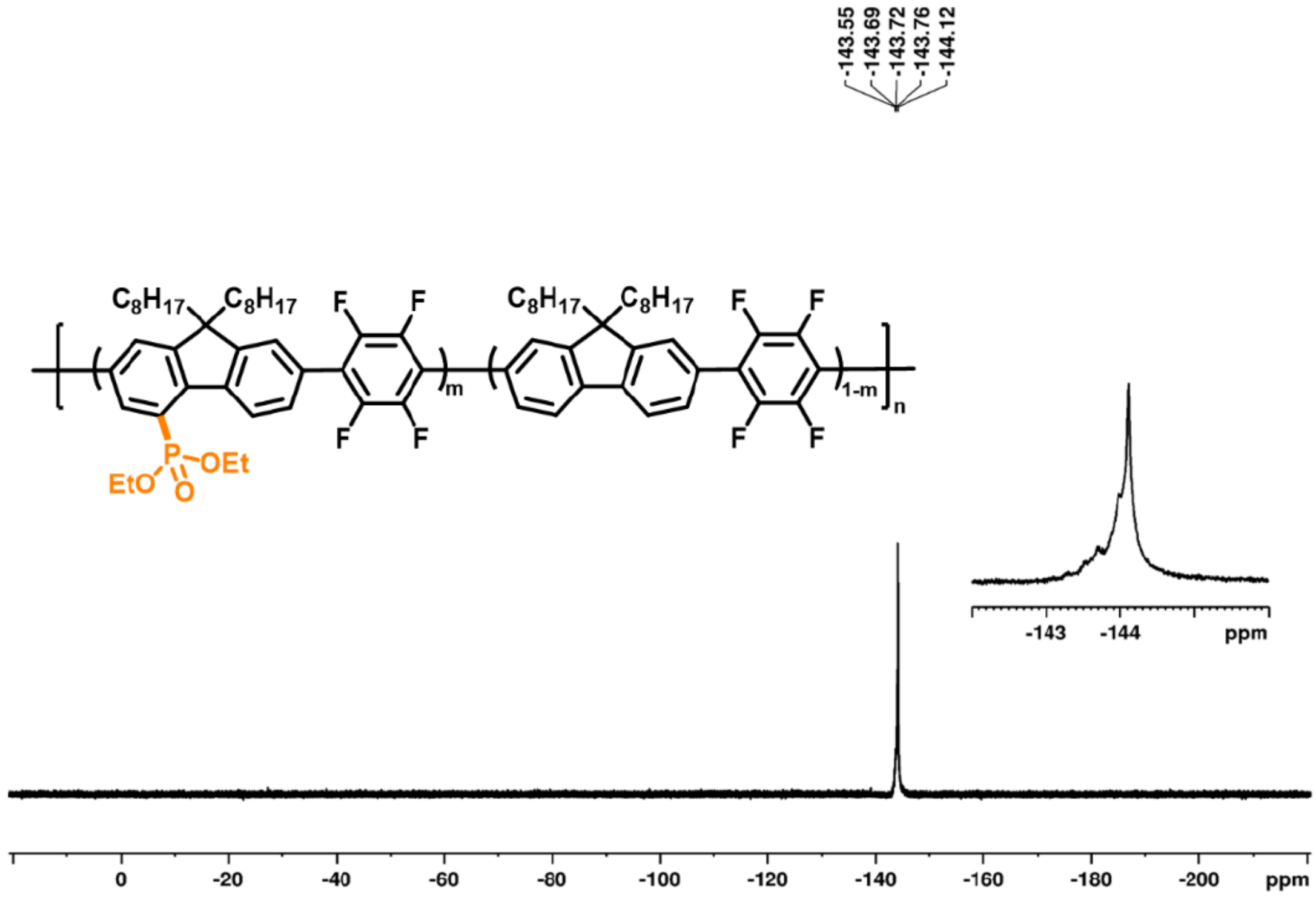

Figure S50. ^{19}F NMR spectrum (470.59 MHz, $CDCl_3$) of P(Fl-TFB-Phos(Et)) (3 h) (**P9**, DOF: 0.44).

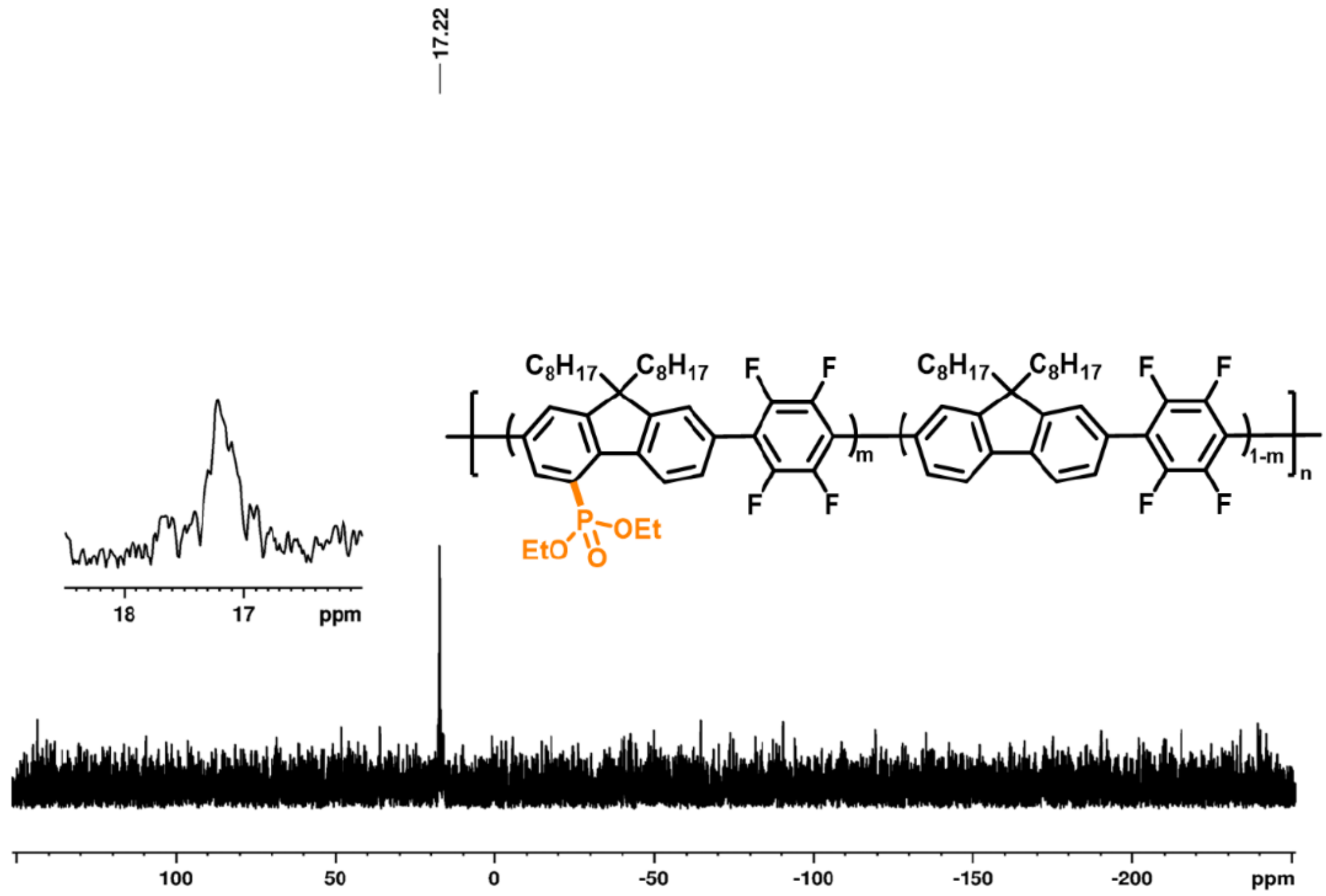

Figure S51. ^{31}P NMR spectrum (200.44 MHz, $CDCl_3$) of P(Fl-TFB-Phos(Et)) (3 h) (**P9**, DOF: 0.44).

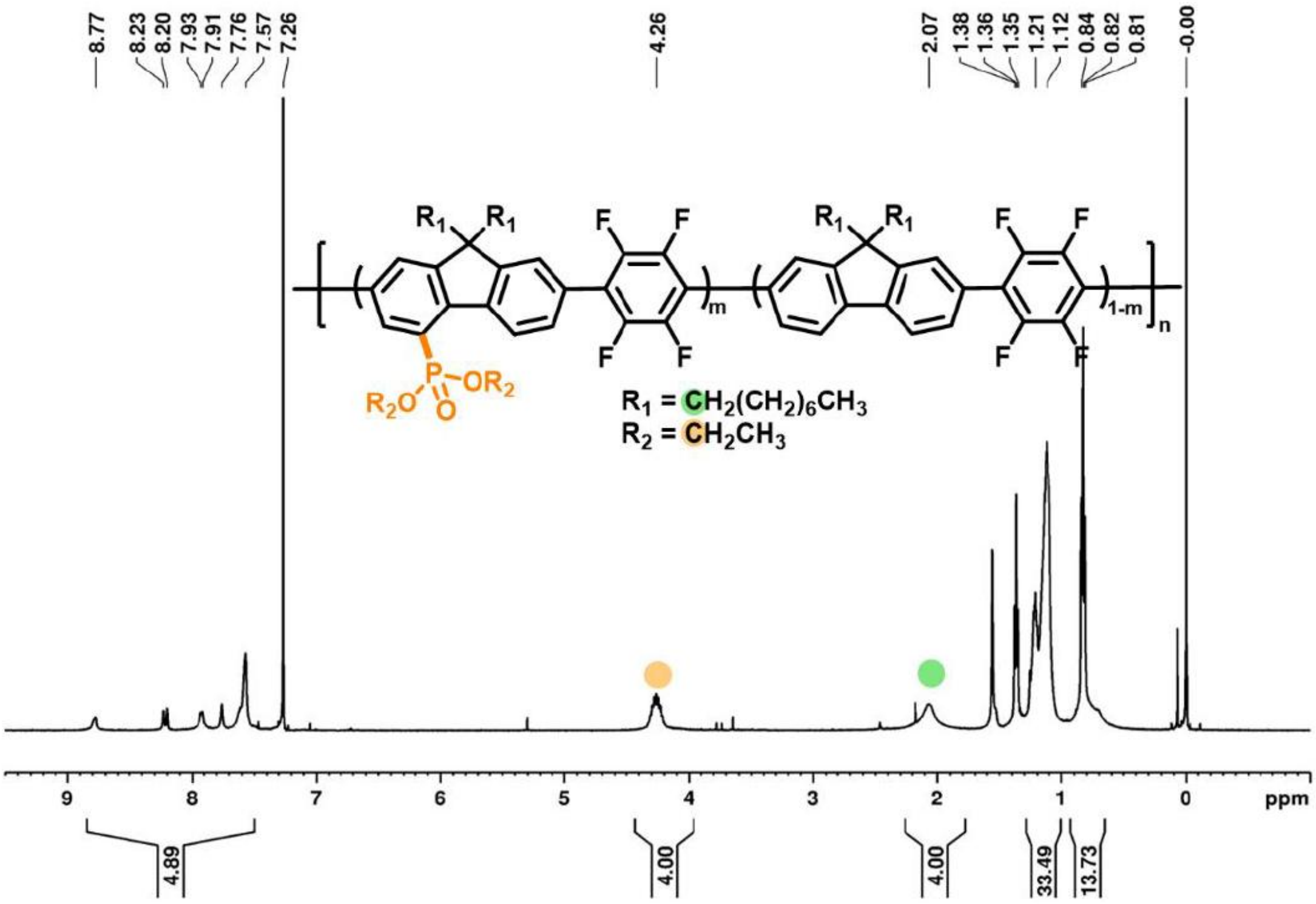

Figure S52. ^{1}H NMR spectrum (500.13 MHz, $CDCl_3$) of P(Fl-TFB-Phos(Et)) (5 h) (**P9**, DOF : 0.78).

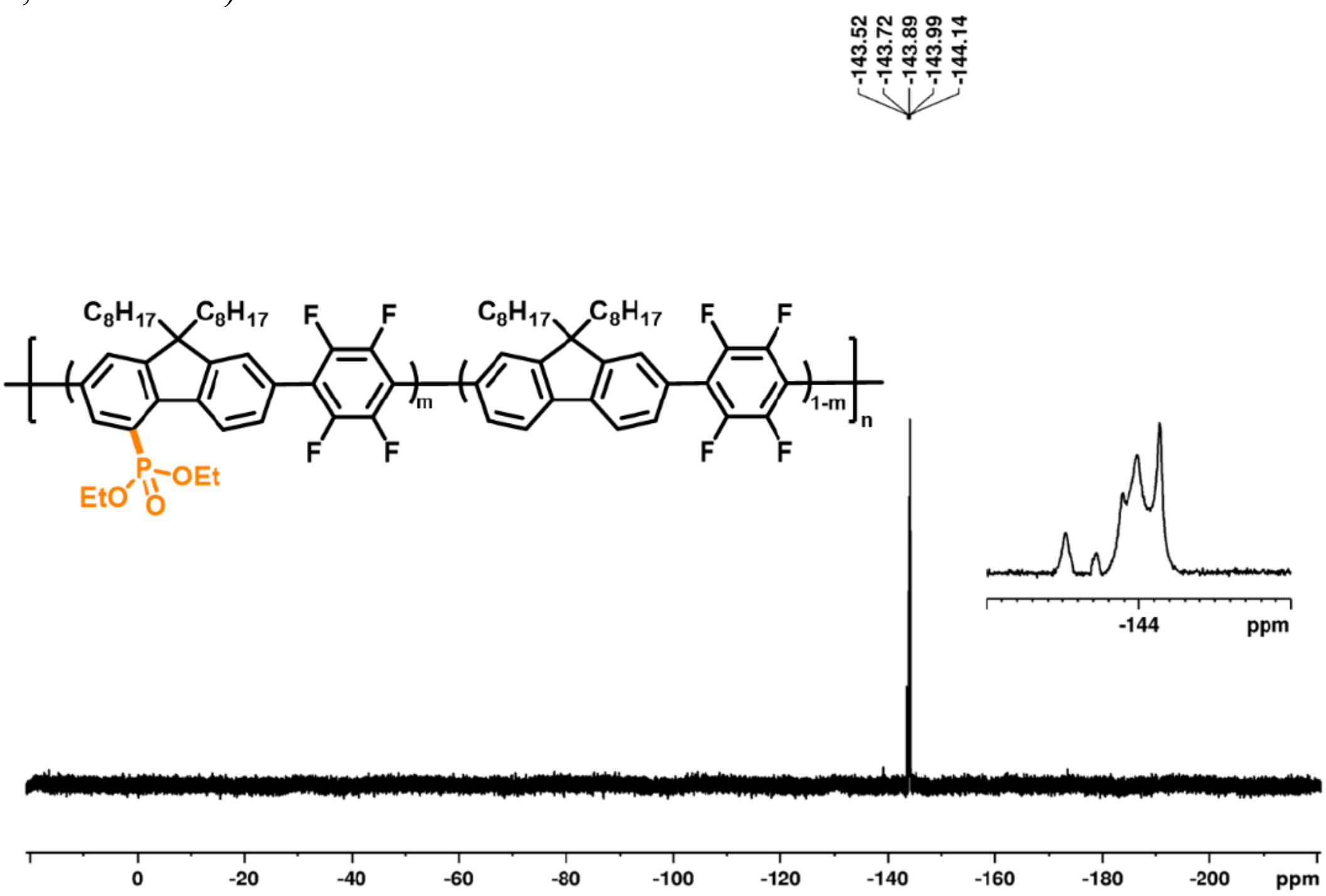

Figure S53. ^{19}F NMR spectrum (470.59 MHz, $CDCl_3$) of P(Fl-TFB-Phos(Et)) (5 h) (**P9**, DOF: 0.78).

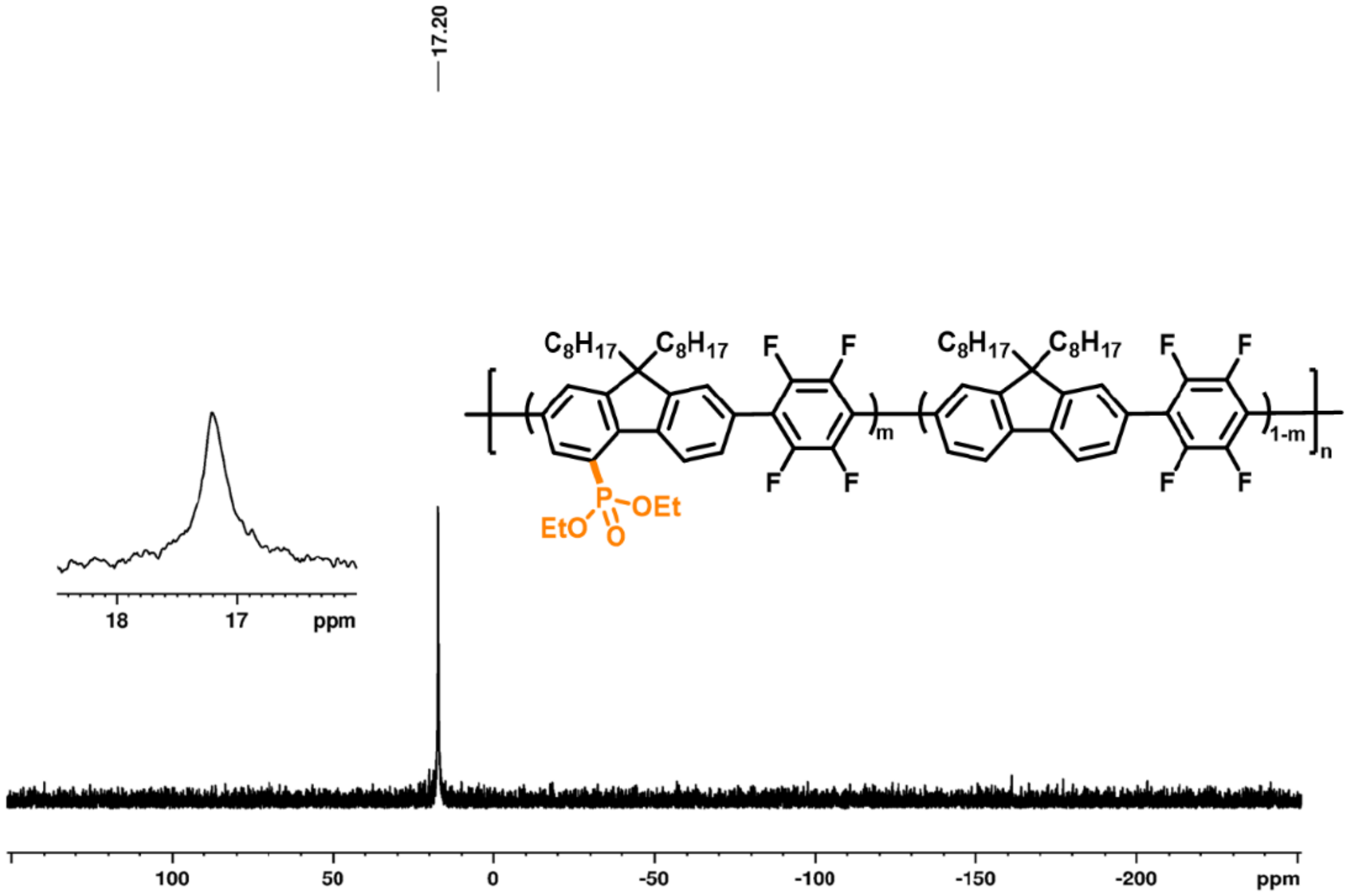

Figure S54. ^{31}P NMR spectrum (200.44 MHz, $CDCl_3$) of P(Fl-TFB-Phos(Et)) (5 h) (**P9**, DOF: 0.78).
